# Molecular orbital node engineering in pyrene: linking chemical reactivity with room-temperature phosphorescence

**DOI:** 10.1093/nsr/nwag394

**Published:** 2026-06-26

**Authors:** Hongping Liu, Dingcheng Zhou, Wei Zhang, Xiaolong Zhang, Chengze Yang, Baicheng Zhang, Aoyuan Cheng, Hao Su, Yang Zhang, Meng Zhou, Xuepeng Zhang, Tao Wang, Guoqing Zhang

**Affiliations:** Hefei National Research Center for Physical Sciences at the Microscale, University of Science and Technology of China, Hefei 230026, China; Hefei National Research Center for Physical Sciences at the Microscale, University of Science and Technology of China, Hefei 230026, China; Hefei National Research Center for Physical Sciences at the Microscale, University of Science and Technology of China, Hefei 230026, China; Hefei National Laboratory, University of Science and Technology of China, Hefei 230026, China; Hefei National Research Center for Physical Sciences at the Microscale, University of Science and Technology of China, Hefei 230026, China; Hefei National Research Center for Physical Sciences at the Microscale, University of Science and Technology of China, Hefei 230026, China; Hefei National Research Center for Physical Sciences at the Microscale, University of Science and Technology of China, Hefei 230026, China; Hefei National Laboratory, University of Science and Technology of China, Hefei 230026, China; Hefei National Laboratory, University of Science and Technology of China, Hefei 230026, China; Hefei National Research Center for Physical Sciences at the Microscale, University of Science and Technology of China, Hefei 230026, China; Hefei National Research Center for Physical Sciences at the Microscale, University of Science and Technology of China, Hefei 230026, China; Hefei National Research Center for Physical Sciences at the Microscale, University of Science and Technology of China, Hefei 230026, China; Beijing Key Laboratory of Construction Tailorable Advanced Functional Materials and Green Applications, School of Materials Science and Engineering, Beijing Institute of Technology, Beijing 100081, China; Hefei National Research Center for Physical Sciences at the Microscale, University of Science and Technology of China, Hefei 230026, China; Hefei National Laboratory, University of Science and Technology of China, Hefei 230026, China

**Keywords:** phosphorescence, chemical reactivity, charge transfer, orbital engineering, pyrene

## Abstract

Substitutions at the nodal and anti-nodal positions of conjugated aromatics exert profound effects on their electronic properties, yet a systematic investigation is lacking on how these underlying quantum mechanical rules are manifested experimentally. Here, using polycyclic aromatic hydrocarbon derivatives as a model system, we systematically elucidate how nodal and anti-nodal substitutions dictate their chemical reactivity and physical properties. It is found that Sonogashira C–C coupling at the nodal position has a noticeably lower product yield than the anti-nodal position under identical reflux conditions due to inhibited molecular orbital amplitude. Comprehensive spectroscopic characterization of the resulting substituted products reveals two striking photophysical differences: (1) Frontier orbital symmetry is largely conserved in node-substituted molecules, whereas the anti-nodal substitution induces strong symmetry-breaking, leading to accelerated fluorescence emission in anti-node-substituted pyrenes; (2) Nodal substitution induces the formation of charge-transfer states due to a twisted geometry, which activates room-temperature phosphorescence via improved singlet–triplet intersystem crossing. This study establishes an effective structure–property relationship linking molecular orbital symmetry to macroscopic cross-coupling reactivity and molecular photophysics within these conjugated aromatics.

## Teaser

This work uncovers how altering specific substitution sites in organic molecules simultaneously controls chemical reactivity and unlocks long-lasting afterglow at room temperature.

## INTRODUCTION

Molecular orbital (MO) theory stands as the bedrock of modern organic chemistry, providing a unified quantum mechanical framework for understanding molecular structure, reactivity and function [1]. Within this framework, frontier MO (FMO) analysis serves as the key element—an indispensable tool for deciphering organic reaction mechanisms, interpreting photophysical phenomena, and guiding the rational design of catalysts [2,3]. By systematically analyzing the distributions of the highest occupied MO (HOMO) and the lowest unoccupied MO (LUMO), MO theory enables chemists to predict and control chemical behavior with remarkable precision [4]. For example, Tkatchenko *et al*. revealed that in the partial hydrogenation of α,β-unsaturated carbonyl compounds, the broader spatial delocalization of the C=C bond’s HOMO–1 relative to the C=O bond’s HOMO accounts for selective reduction [5]. Zhang *et al*. introduced the aromatic electronic directing rule for predicting luminescence in disubstituted benzenes based on FMO-derived electron-density distributions [6]. Lu *et al*. showed that deliberate FMO engineering in single-atom catalysts leads to highly active and stable hydrogenation systems [7]. Beyond these applications, MO phase information is also crucial in understanding pericyclic reactions, where the Woodward–Hoffmann rules use the symmetry and phase relationships of interacting orbitals to predict whether reactions are thermally or photochemically allowed or forbidden [8]. In the Diels–Alder reaction, the constructive overlap between the diene’s HOMO and the dienophile’s LUMO requires proper phase alignment, directly determining the feasibility and stereochemistry of the cycloaddition [9].

**Figure 1.**
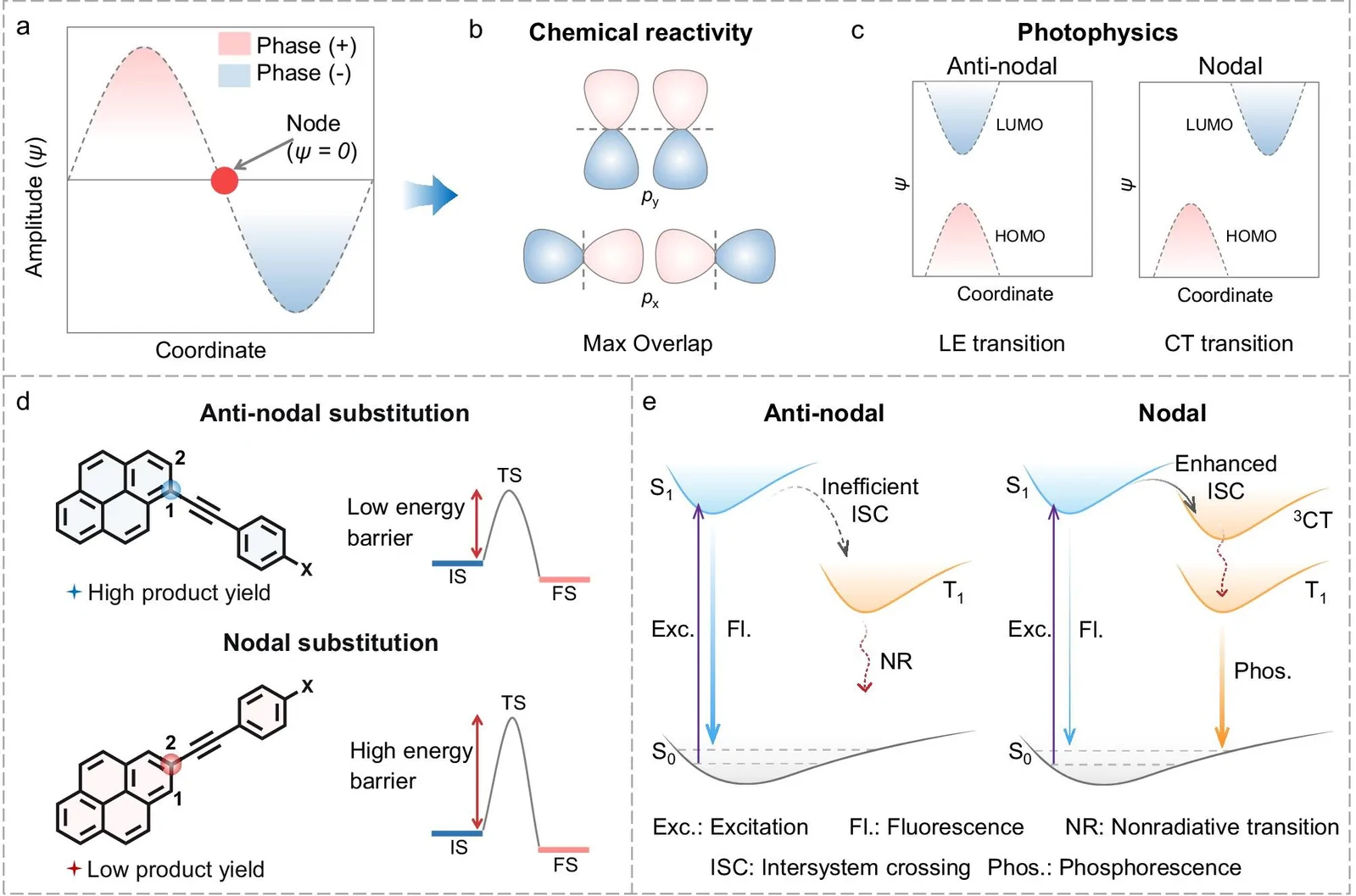
Design principle and schematic illustration of the nodal effect on chemical reactivity and photophysical processes. (a) Schematic of orbital phases and nodes (left). (b) Schematic of maximum orbital overlap that facilitates bonding using *p*-orbitals as an example. (c) Schematic of chemical substitution at the anti-nodal and nodal positions, leading to separated or overlapped FMOs and thus locally excited (LE) or charge-transfer (CT) transitions. (d) Correlation between nodal substitution in pyrene derivatives and product yield (IS, initial state; TS, transition state; FS, final state). (e) Simplified Jablonski diagrams illustrating how nodal substitution influences phosphorescence.

Nodes, regions where the wave function or MO has zero amplitude (Fig. 1a), not only define orbital symmetry and phase but also govern where and how molecules interact with reagents or absorb light [10]. Therefore, the spatial distribution of nodes within MOs exerts a profound influence on both the chemical reactivity and the photophysical properties of organic molecules (Fig. 1b and c) [11]. The presence of nodes at specific atomic sites can inhibit or enhance orbital overlap during bond formation, thus controlling regioselectivity and chemoselectivity in reactions such as electrophilic aromatic substitution or cycloadditions [12]. In addition to determining chemical reactivity, nodal patterns in the FMOs can theoretically govern the allowance and intensity of electronic transitions by influencing transition dipole moments (TDMs) and selection rules [13], thereby contributing to distinct exciton dynamics.

Organic molecular phosphorescence is a type of luminescence that arises from the radiative decay of triplet excitons, where the spin-forbidden T₁→S₀ transition typically leads to long-lived emission [14–16], differing from fluorescence from a spin-allowed S₁→S₀ transition. Traditionally, efficient room-temperature phosphorescence (RTP) in organic materials has been achieved by incorporating heavy atoms to enhance spin–orbit coupling (SOC) [17–21], promote intersystem crossing (ISC), and thus enable RTP with lifetimes exceeding microseconds [22–26]. Driven by versatile applications in information encryption [27–30], imaging [31–34], and optoelectronics [35–38], heavy-atom-free RTP materials have been developed via judicious molecular design that intrinsically manipulates orbital interactions based on MO theory [39–41]. While some studies have demonstrated that altering substitution patterns on the pyrene core could modulate singlet photophysics [42–46], how MO nodes and anti‑nodes modulate triplet-related dynamics, such as ISC and triplet radiative decay, has not been systematically clarified.

To elucidate the role of MO nodes in regulating molecular excited states, one may expect that by introducing electron-donating or electron-withdrawing groups at anti-nodal sites of an aromatic molecule, where the orbital amplitude is maximal, the π-conjugation length is extended. This usually increases the TDM of the substituted compound according to Equation (1):


(1) \begin{eqnarray*}
{\boldsymbol{\mathop \mu \limits^{\rightarrow} }_{j \leftarrow i}} = \left\langle {j\left| {\hat{\boldsymbol\mu }} \right|i} \right\rangle \ = \ \int \it {\Psi} _j^*\left( \boldsymbol{\mathop r\limits^{\rightarrow} } \right)\,
{\hat{\boldsymbol\mu }}\,{{\it {\Psi} }_i}\left( \boldsymbol{\mathop r\limits^{\rightarrow} } \right)\ {\mathrm{d}}\!\boldsymbol{\mathop r\limits^{\rightarrow}},
\end{eqnarray*}

where ${{\it \Psi }_i}( \boldsymbol{\mathop r\limits^{\rightarrow} } )$ and $\it \Psi _j^*( \boldsymbol{\mathop r\limits^{\rightarrow} } )$ are the wave functions of the initial and final states, respectively, and $\hat{\boldsymbol\mu }\ $is the electric-dipole operator. From the equation, as effective conjugation or average *\boldsymbol r * increases, and wave-function symmetry is reduced, TDM is expected to increase sharply [47,48]. The effect is particularly dramatic for highly symmetric cores, as anti-nodal substitution breaks symmetry during electronic transitions, leading to a TDM enhancement by orders of magnitude [49,50]. As a result, the influence of anti-nodal substitution is 2-fold: (1) an increase in the TDM inevitably reduces ISC efficiency, and (2) the extended π-conjugation enriches molecular motion modes (e.g., vibration and translation) and lowers the triplet-excited-state energy, thereby promoting triplet nonradiative transitions. The combined effect is expectedly a reduction in the RTP quantum yield [51–53]. Conversely, substitution at nodal positions minimally perturbs the electronic structure of the aromatic core, resulting in negligible ground-state electronic conjugation and substantial rotational freedom of the substituent at room temperature. In this scenario, RTP is enhanced when the substituent and the core exhibit some degree of charge-transfer (CT) character, which mixes singlet and triplet spin multiplicities.

Because MO nodes affect both chemical reactivity and photophysics, establishing their connection helps define structure–property relationships for materials design and versatile applications. Here, we introduce a series of pyrene derivatives substituted at the 1-position (anti-node) and the 2-position (node) (Fig. 1d, left). These compounds advance the understanding of substitution effects by explicitly establishing the relationship between the MO nodal phase and triplet excited-state dynamics. It is demonstrated that nodal substitution affords a lower product yield than anti-nodal substitution under the same reaction conditions, due to vanished MO amplitude at the node that leads to a higher reaction energy barrier (Fig. 1d, right). For excited-state dynamics, 1-substituted pyrenes exhibit accelerated fluorescence decay with concomitant competition from the nonradiative decay of triplet excitons due to symmetry breaking (Fig. 1e, left), whereas 2-substituted pyrenes convey the symmetry-induced slow fluorescence decay and CT-enhanced ISC, thereby leading to improved RTP (Fig. 1e, right).

## RESULTS

### Chemical reactivity investigations

To investigate how nodes regulate chemical reactivity, a series of pyrene derivatives with substituents at 1- and 2-positions were designed (Fig. 2a), with phenanthrene derivatives as supporting examples. These model compounds were synthesized via Pd-catalyzed Sonogashira coupling reactions (details in Schemes S1–S13). Their structures were confirmed by ^1^H and ^13^C nuclear magnetic resonance (NMR; Figs S1–S26), high-resolution mass spectrometry (Figs S27–S39), and single-crystal X-ray diffraction (SCXRD; Figs S40–S43), with corresponding purities exceeding 99% (Figs S44 and S45). We found that the regioselectivity and yield are significantly influenced by the distribution of MOs, particularly the LUMO of brominated pyrene, which is subsequently involved in the oxidative addition with active Pd(0) [54]. Specifically, the yields of anti-node-substituted derivatives are nearly twice those of their node-substituted isomers (**1-H-Py:** 76.8%, **1-OME-Py:** 85.4% and **1-CN-Py:** 62.8%, **2-H-Py:** 36.5%, **2-OME-Py:** 44.8% and **2-CN-Py:** 34.3%; Fig. 2a). In addition, the reaction yields decrease as the electron-withdrawing strength of the substituents increases, consistent with the principle of the Sonogashira reaction, i.e., electron-deficient alkynes favor the coupling process [55].

**Figure 2.**
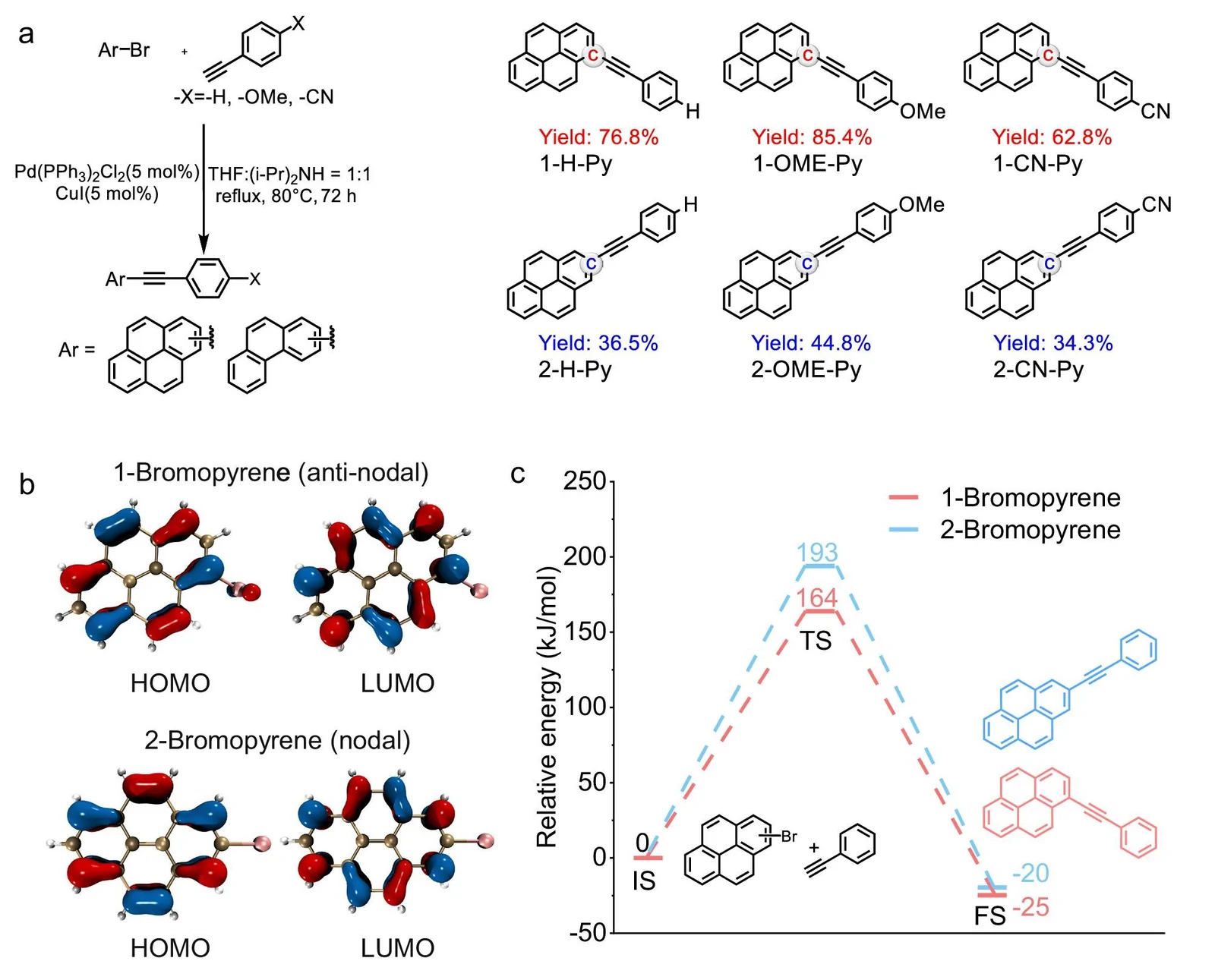
(a) Schematic of the synthetic route and structures of the targeted pyrene derivatives. The Pd(II) precatalyst is reduced *in situ* by diisopropylamine to generate the active Pd(0) species. (b) Calculated HOMO and LUMO distributions of 1-bromopyrene and 2-bromopyrene. (c) Calculated energy differences between reactants and products based on 1-bromopyrene and 2-bromopyrene (IS: initial state, TS: transition state, FS: final state).

To rationalize this disparity, HOMO and LUMO distributions of starting materials 1- and 2-bromopyrene were calculated at the B3LYP [56]/6-31G(d) [57] level based on density functional theory (DFT). As expected, 1-bromopyrene has a larger carbon-centered LUMO distribution at the 1-position (Fig. 2b), indicative of a greater anti-bonding orbital σ* coefficient, while 2-bromopyrene exhibits negligible LUMO amplitude at the 2-position. Consequently, 1-bromopyrene is more capable of accepting the symmetry-matched Pd(0) *d*-orbital for oxidative addition to form the intermediate state, the rate-determining step in Sonogashira coupling. We also approximately calculated the reaction energy barriers using 1- and 2-bromopyrene as reactants. It is important to note that while the experimental Sonogashira coupling proceeds via catalytic cycles involving Pd and Cu rather than the direct collision modeled, the results could qualitatively indicate a higher energy barrier for 2-bromopyrene (Fig. 2c), leading to a lower product yield.

### Theoretical calculations

**Figure 3.**
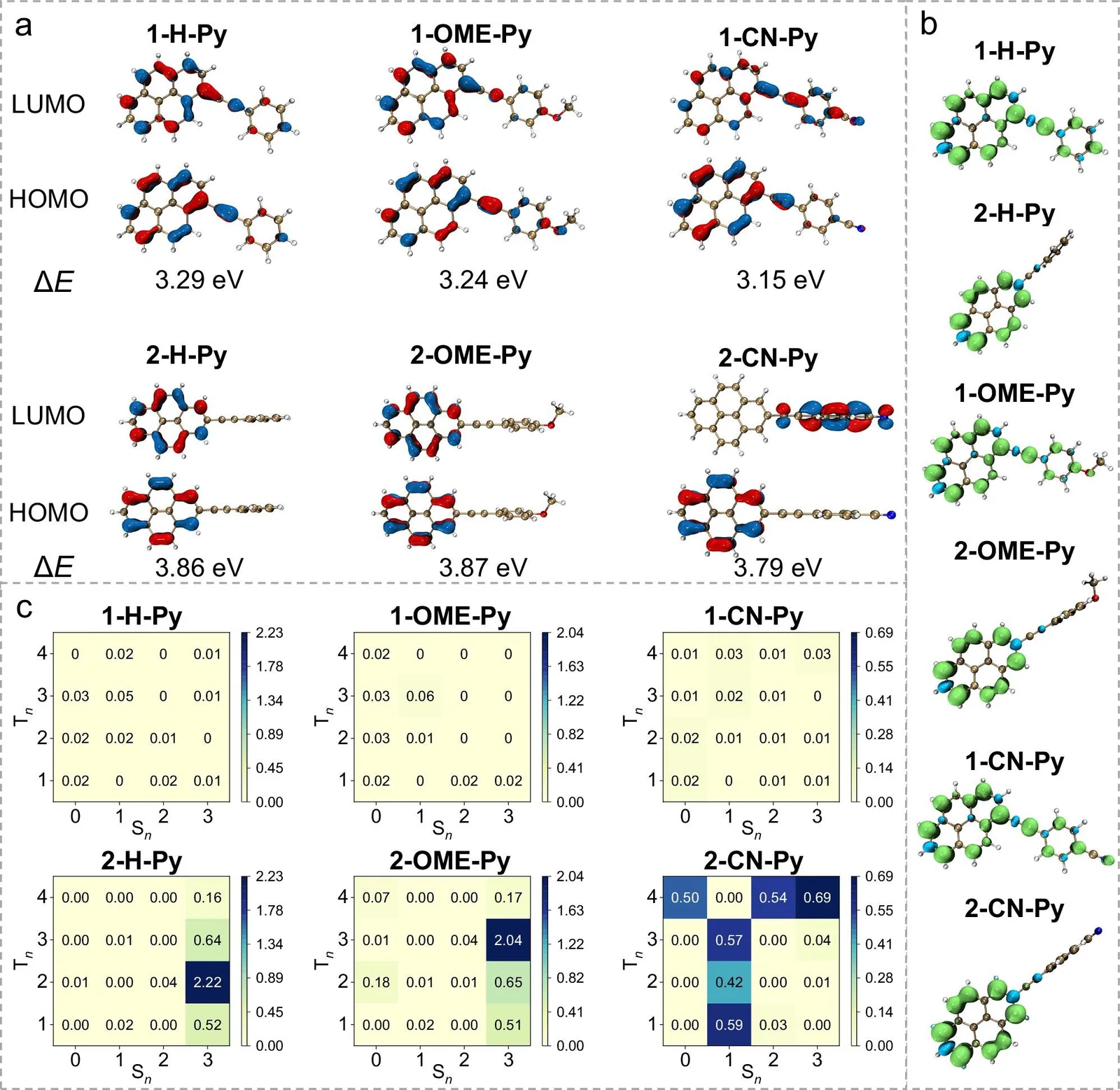
Theoretical calculations. (a) Calculated FMO distributions and energy levels of 1- and 2-substituted pyrenes with associated orbital energies at optimized S_0_ geometries. (b) T_1_ spin density distributions (isovalue: 0.004) calculated at optimized T_1_ geometries. (c) Calculation of SOC constants of 1- and 2-substituted pyrenes.

To connect the orbital node, chemical reactivity, and photophysics, we next systematically performed DFT calculations on the synthesized pyrene–phenylacetylene derivatives to predict their photophysical properties prior to spectroscopic measurements. The calculated HOMO–LUMO energy gap (Δ*E*_HL_) for these derivatives ranges from 3.1 to 3.9 eV (Fig. 3a). Notably, the derivatives substituted at the nodal 2-position of the pyrene skeleton exhibit larger Δ*E*_HL_ than those substituted at the anti-nodal 1-position, and the introduction of methoxy or cyano substituents has only a minor effect on this gap. The calculations reveal a node at the 2-position of pyrene, indicating that the 2-substituted derivatives adopt a non-conjugated junction between the pyrene ring and the alkynyl linker. This non-conjugated structure explains why the Δ*E*_HL_ in the 2-substituted derivatives is relatively insensitive to changes in the electron-donating or electron-withdrawing character of substituents on the phenylacetylene moiety. In other words, the electronic influence of the substituent cannot propagate effectively through the entire π-conjugated system, thereby limiting its impact on the Δ*E*_HL_. Theoretical models indicate that the pyrene plane and the benzene ring tend to adopt nearly perpendicular orientations to minimize steric repulsion in the absence of π-conjugation. This is supported by experimental crystallographic data (Figs S43 and S44). The dihedral angle in **2-H-Py** is 26.53°, much larger than 2.53° in **1-H-Py**. It should be noted that the dihedral-angle difference between the single-crystal structure and the calculated isolated molecule geometry arises from lattice effects on the equilibrium geometry, confirming the presence of a highly rotational structure in the 2-substituted derivatives, which thus influences the electronic properties.

These computational insights also rationalize the observed product yields in the Sonogashira coupling reaction. According to MO theory, the reaction involves net electron transfer from the nucleophile’s HOMO to the electrophile’s LUMO, forming new chemical bonds. However, for 2-substituted derivatives, the lack of electron wave-function distribution at the nodal site prevents effective orbital overlap with the alkyne, making the LUMO largely inaccessible for coupling. Instead, the reaction must engage a higher-energy orbital such as LUMO+1, where the substitution site becomes an anti-node (Fig. S46). Furthermore, the distribution of the HOMO and LUMO wave functions reveals that **2-CN-Py** is unique among the derivatives, whose HOMO is localized primarily on the pyrene core, while its LUMO is confined on the phenylacetylene moiety (Fig. 3a). This suggests a strong intramolecular CT character in this molecule.

From a quantum mechanical perspective, transitions between the HOMO and LUMO in unsubstituted pyrene are forbidden due to the high symmetry of the wave functions. Our calculations predict that 1-substituted pyrenes disrupt the symmetry and extend conjugation, thereby enhancing fluorescence. By contrast, the high symmetry is maintained in the 2-substituted isomers, which weakens absorption and slows fluorescence emission because of the more forbidden nature of the transition. Time-dependent DFT calculations further indicate that 1-substituted pyrenes have lower T_1_ energies than 2-substituted counterparts, consistent with the stabilization of the T_1_ states by extended π-conjugation (Fig. S47). As expected, the T_1_ spin densities of 1-substituted pyrenes distribute across the entire molecular skeleton, while those of 2-substituted pyrenes are mainly localized on the pyrene core (Fig. 3b). Similarly, hole–electron analysis on the T_1_ state reveals that 2-substituted pyrenes have hole and electron distributions localized on the pyrene core, whereas 1-substituted analogues show delocalized distributions extending to the substituents (Fig. S48).

To evaluate the likelihood of singlet–triplet ISC, SOC constants were computed (Fig. 3c). Compared to **1-H-Py** and **1-OME-Py, 2-H-Py** and **2-OME-Py** show similar forbidden S_1_–T*_n_* ISC (*n* = 1–4), but exhibit relatively efficient ISC between higher-lying singlet and triplet manifolds. This result is consistent with our previous observations in twisted organic systems [58], where twisted geometries can boost ISC between high-lying singlet and triplet states, even when such pathways are not favored by El-Sayed’s rule. Notably, because strong intermolecular CT occurs in **2-CN-Py**, enhanced SOC is obtained between S_1_ and T*_n_* (*n* = 1–4), with CT states serving as intermediates to facilitate spin flipping. Based on these calculations, we propose that 2-substituted pyrenes exhibit more efficient phosphorescence than their 1-substituted analogues.

### Photophysical investigations

**Figure 4.**
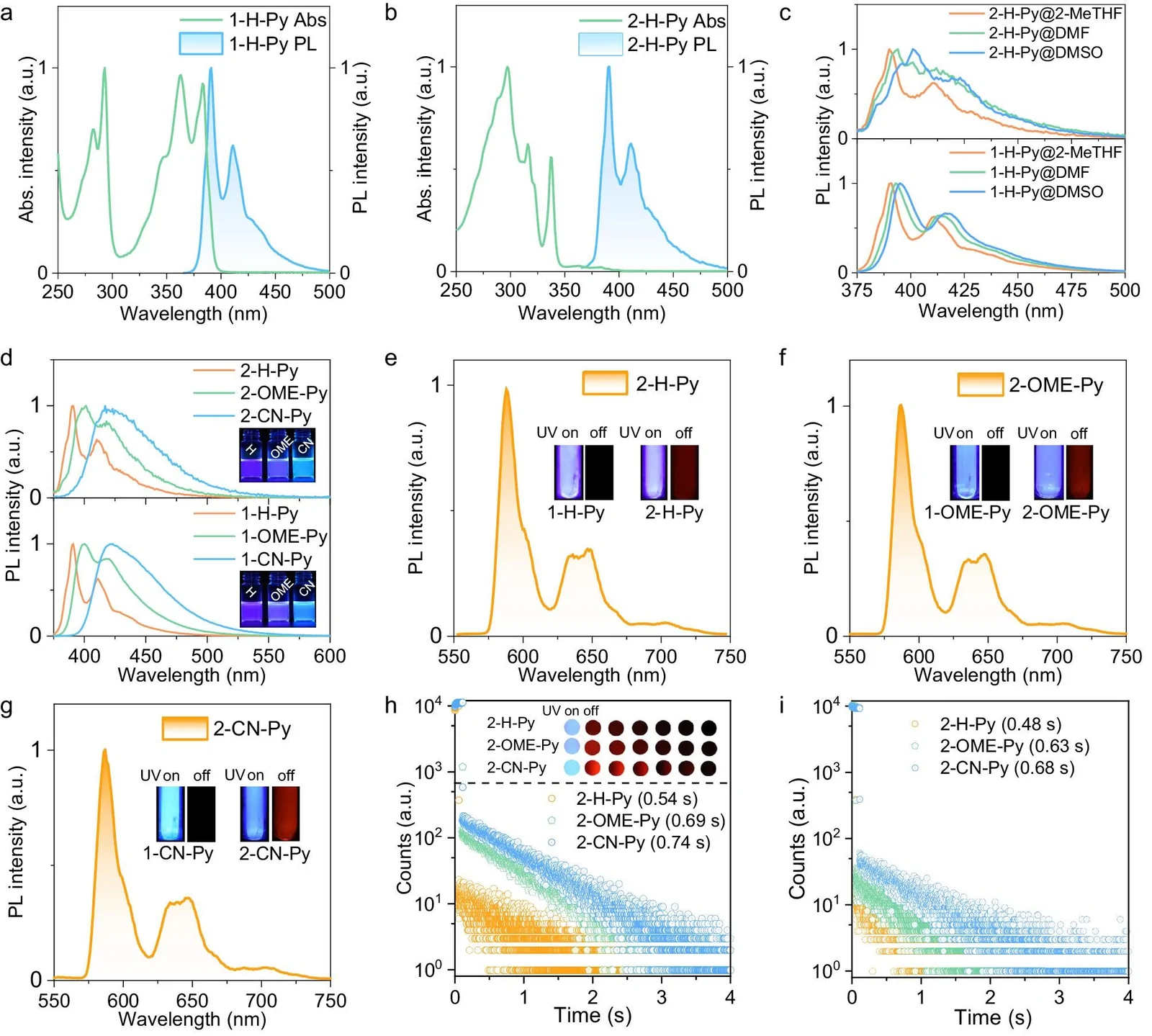
Absorption (Abs.) and photoluminescence (PL) investigations of 1- and 2-substituted pyrenes in dilute solution (*c* = 1 × 10^−5^ M). (a and b) UV–vis absorption and steady-state PL (*λ*_exc_ = 350 nm) spectra of (a) **1-H-Py** and (b) **2-H-Py** in 2-MeTHF recorded at 298 K. (c) Solvatochromic study in various solvents (*λ*_exc_ = 350 nm). (d) Steady-state PL spectra recorded in 2-MeTHF at 298 K (*λ*_exc_ = 350 nm); inset: photos showing luminescence in 2-MeTHF under 365-nm UV excitation. (e–g) Delayed PL spectra of (e) **2-H-Py**, (f) **2-OME-Py**, and (g) **2-CN-Py** recorded in 2-MeTHF at 77 K (gated time: 5–100 ms; λ_exc_ = 298 nm); insets: photos showing luminescence with 365-nm UV lamp on and off. (h and i) Time-resolved phosphorescence decay profiles recorded at (h) 587 and (i) 649 nm in 2-MeTHF at 77 K (*λ*_exc_ = 285 nm); inset: photos showing evolving afterglow after 365-nm UV lamp switched off.

Guided by theoretical calculations, the ultraviolet–visible (UV–Vis) absorption and steady-state photoluminescence (PL) spectra of these pyrene derivatives were measured in dilute 2-methyltetrahydrofuran (2-MeTHF) solution (Fig. 4a and b). As predicted from the higher π-conjugation in the 1-substituted derivatives, **1-H-Py** exhibits a red-shifted absorption compared to **2-H-Py**, confirming its extended electronic conjugation. The UV–vis spectrum of **1-H-Py** displays an additional intense absorption band peaking at 363 and 383 nm that are absent in **2-H-Py**, consistent with the theoretical prediction that 1-position substitution disrupts the original high symmetry of the pyrene core, enabling new allowed electronic transitions. This nodal effect can also be observed in dichloromethane (DCM; Fig. S49), confirming that the extended conjugation arises from the substitution pattern, independent of the solvent used. The measured excitation–emission maps of **1-H-Py** and **2-H-Py** further validate these observations, where **1-H-Py** exhibits more abundant excitation channels and a slightly red-shifted excitation profile compared to **2-H-Py** (Fig. S50). The effect of different substituents on the benzene ring aligns with computational expectations that introducing methoxy or cyano groups induces a red shift in the absorption of 1-substituted derivatives, while 2-substituted pyrenes show no obvious red shift in optical gap due to minimal orbital perturbation from node substitutions, aside from intensity differences (Fig. S51).

For steady-state PL measurements, both **1-H-Py** and **2-H-Py** show similar structured vibronic emissions with two main peaks at ∼390 nm and 410 nm (Fig. 4a and b), but solvent-polarity responses diverge (Fig. 4c and Table S1). Although both emitters exhibit limited sensitivity to solvent polarity, **2-H-Py** has a redder spectral shift from 390 to 401 nm. In contrast, **1-H-Py** retains its vibronic structure with only slight shifts from 390 to 395 nm. This difference arises from their distinct excited-state characters. The anti-nodal substitution in **1-H-Py** promotes extended conjugation and structural rigidity, resulting in an emission dominated by the LE character. Conversely, the nodal connection in **2-H-Py** electronically decouples the substituent, affording greater rotational freedom. This flexibility facilitates the formation of a CT state upon excitation. The CT state possesses a larger dipole moment, which is selectively stabilized by polar solvents via dipole–dipole interactions, leading to the observed red shift and broadened spectral profile.

We then compared the steady-state PL spectra of these pyrene derivatives in 2-MeTHF and found that with the introduction of substituents both 1- and 2-substituted pyrenes display gradually red PL shift at room temperature (Fig. 4d and Table S2), among which especially the cyano-substituted pyrenes have the largest shifts of 30 nm for **1-CN-Py** and 26 nm for **2-CN-Py**. Similar red-shifted emissions observed at 77 K further support the emergence of CT emission (Fig. S52). Time-resolved techniques detected PL lifetimes at associated emission maxima. Consistent with the orbital analysis, 1-substituted pyrenes at the anti-node have faster singlet radiative decay than 2-substituted isomers at both room temperature and 77 K (Figs S53, S54, and Table S2), indicating symmetry-breaking-induced enhancement of the S_1_→S_0_ oscillator strength. This consistent trend was also observed in dichloromethane (Fig. S55), demonstrating that the prolonged fluorescence lifetime in the nodal pyrene derivatives originates intrinsically from their unique excited-state dynamics rather than specific solvent interactions. The PL quantum yields (PLQYs) of the pyrene derivatives were then measured (Table S3). As expected, anti-node-substituted pyrenes have higher total PLQYs than their nodal counterparts, due to less forbidden singlet radiative transitions.

Beyond steady-state PL, we further explored the nodal effect on phosphorescence using time-gating techniques. At 77 K in 2-MeTHF glass, 2-substituted pyrenes display distinct phosphorescent afterglows, whereas 1-substituted analogues show no observable phosphorescence under identical conditions after ceasing 365-nm UV excitation. This behavior was also recorded in DCM at 77 K (Fig. S56). As discussed above, the activated phosphorescence in 2-substituted pyrenes is because their twisted molecular geometries help form intramolecular CT states that improve ISC and suppress fluorescence decay due to symmetry-forbidden transitions. The measured phosphorescence spectra of 2-substituted pyrenes feature similar emissions peaking at 587 and 649 nm (Fig. 4e–g). The excitation spectra measured at these two emission wavelengths show almost the same spectral profile except for intensity (Fig. S57), suggesting these two phosphorescence peaks stem from the same species—triplet excitons localized on the pyrene core, evidenced by the identical T_1_ spin density distribution on the core (Fig. 3b).

Upon substitution of phenylacetylene with methoxy and cyano groups, the resulting **2-OME-Py** and **2-CN-Py** both show gradually enhanced phosphorescence brightness in 2-MeTHF at 77 K compared to **2-H-Py**, as reflected by phosphorescence photos (Fig. 4e–g, insets). The measured time-resolved phosphorescence decay confirms that **2-OME-Py** and **2-CN-Py** have longer phosphorescence lifetimes than **2-H-Py** at both 587 and 649 nm in 2-MeTHF at 77 K (Fig. 4h, i and Table S4). Among them, **2-CN-Py**, with the strongest electron-withdrawing cyano group, exhibits the brightest phosphorescence and the longest lifetime, attributed to enhanced intramolecular CT, which facilitates more efficient ISC. The highly twisted geometry induces mixing between ^3^CT and ^3^LE states via relaxation, leading to observed ^3^LE-dominated phosphorescence from the pyrene core.

**Figure 5.**
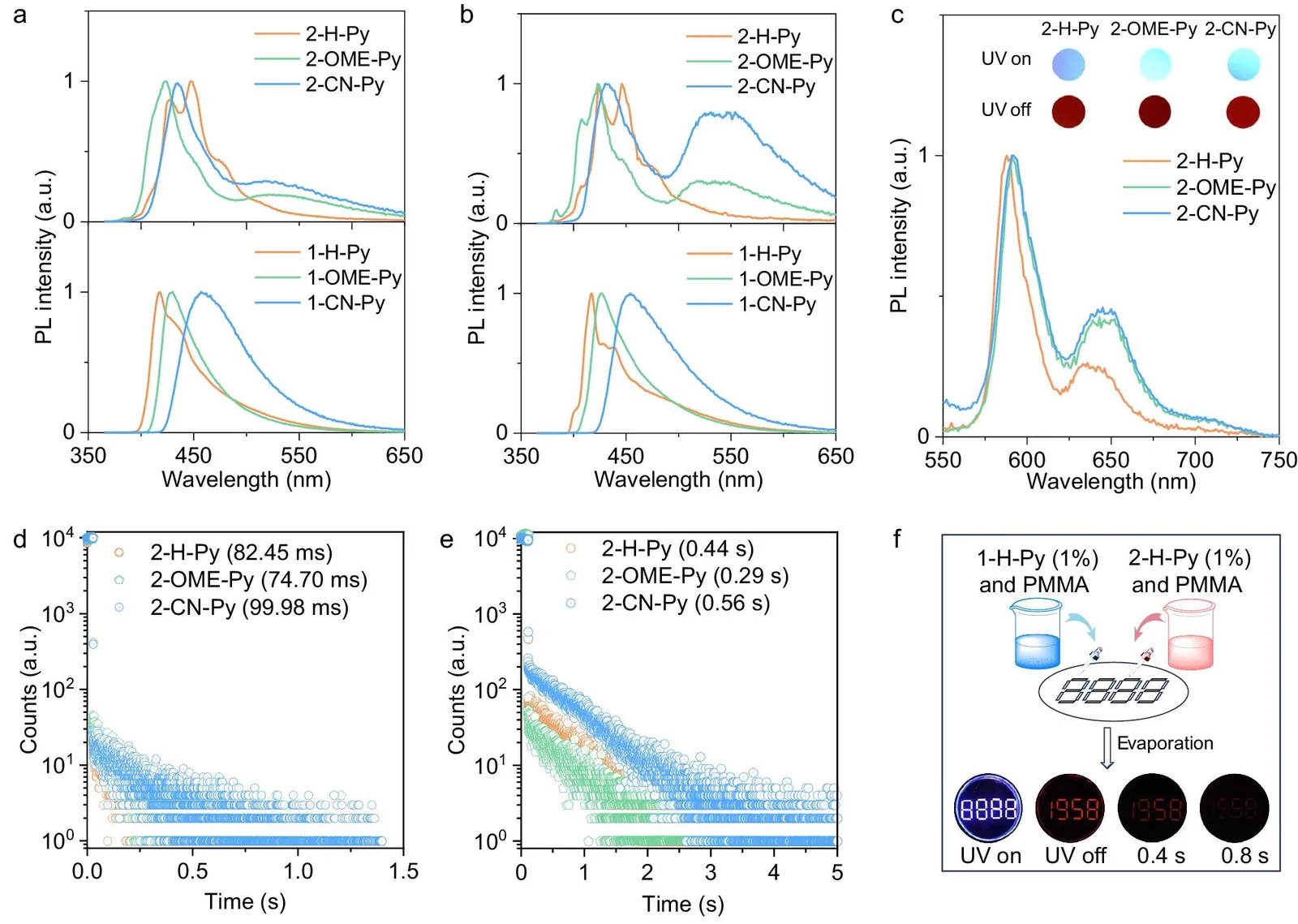
PL investigations of 1- and 2-substituted pyrenes doped in PMMA. (a and b) Steady-state PL spectra recorded at (a) 298 K and (b) 77 K (doping ratio: 0.5 wt%; *λ*_exc_ = 350 nm). (c) Delayed PL spectra recorded at 298 K (doping ratio: 0.5 wt%; gated time: 5–100 ms; *λ*_exc_ = 298 nm); inset: photos showing luminescence with 365-nm UV lamp on and off. (d and e) Time-resolved phosphorescence decay profiles recorded at emission maxima at (d) 298 K and (e) 77 K (doping ratio: 0.5 wt%; *λ*_exc_ = 285 nm). (f) Encryption demonstration using **1-H-Py** and **2-H-Py** in PMMA (doping ratio: 1 wt%).

Furthermore, the photophysical properties of these pyrene derivatives for RTP activation were investigated. The pyrene derivatives were doped into poly(methyl methacrylate) (PMMA) at 0.5 wt% to suppress nonradiative decay of triplet excitons. The steady-state PL spectra of 1-substituted pyrenes in PMMA film exhibit a pronounced red shift from 418 to 457 nm with increasing substituent polarity, accompanied by spectral broadening and loss of vibronic structure (Fig. 5a, bottom), consistent with extended π-conjugation and reduced Δ*E*_HL_ predicted by the calculations (Fig. 3a). However, the steady-state PL spectra of 2-substituted pyrenes display a slight blue shift (Fig. 5a, top). This observation can be rationalized by the rigidochromic effect [59,60]. Differing from 1-substituted pyrenes, 2-substituted derivatives mainly adopt highly rotational twisted geometries in the single-molecule state. The increased rigid environment heavily restricts conformational freedom and raises the energy barrier to form CT states. Similar PL behavior can be also observed at 77 K (Fig. 5b). It was also demonstrated that 1-substituted pyrenes have shorter fluorescence lifetimes than 2-substituted isomers in PMMA at both room temperature and 77 K (Fig. S58, Table S5 and S6), due to the symmetry-breaking-accelerated S_1_→S_0_ radiative transition, agreeing with the measurements recorded in 2-MeTHF.

Notably, comparing steady-state PL spectra of 2-substituted pyrenes at room temperature and 77 K, we can observe a clear emission enhancement around 500–650 nm, indicative of the presence of temperature-sensitive triplet excitons. To verify this, delayed PL measurements were performed. As expected, the measured phosphorescence spectra of 2-substituted pyrenes show obvious structured RTP emissions (Fig. 5c), alongside RTP lifetimes of 80–100 ms (Fig. 5d and Table S7); a red afterglow is visible upon ceasing UV excitation (Fig. 5c, inset). Similar to the recorded PL in 2-MeTHF, no phosphorescence can be observed in 1-substituted pyrenes in PMMA at both room temperature and 77 K. At 77 K, 0.5 wt% 2-substituted pyrenes in PMMA exhibit almost identical emission profiles to those in 2-MeTHF (Fig. S59), with narrower, more finely structured characteristics than at room temperature due to strongly suppressed vibronic modes, as reflected by significantly prolonged phosphorescence lifetimes of 0.4–0.6 s (Fig. 5e and Table S8). Notably, although 2-substituted pyrenes have the same triplet exciton origin, the excitation spectra recorded at the same phosphorescence wavelength (587 nm) are greatly different (Fig. S60), indicating substituent-dependent optimal excitation pathways. Additionally, using DCM solutions of 1 wt% **1-H-Py** and **2-H-Py** doped in PMMA as inks, we demonstrated that information encryption could be achieved by these two organic isomers (Fig. 5f). Specifically, the digits ‘1958’ (founding year of USTC) were written on the filter paper. Upon removal of 365 nm irradiation, only the **2-H-Py**/PMMA pattern remained visible because the **1-H-Py**/PMMA regions are RTP-silent.

To further demonstrate the generality of the nodal engineering in predicting chemical reactivity and activating RTP, additional examples in PMMA were studied (doping ratio: 0.5 wt%). First, **4-H-Py** bearing the phenylacetylene substituent at the 4-position (an alternative anti-node on pyrene) was synthesized. Similar to **1-H-Py, 4-H-Py** affords a higher product yield than **2-H-Py** but exhibits negligible RTP emission in doped PMMA (Fig. S61). In addition, derivatives with strongly electron-donating (dimethylamino: **1-N(Me)₂-Py** and **2-N(Me)₂-Py**) and strongly electron-withdrawing (nitro: **1-NO₂-Py** and **2-NO₂-Py**) groups were investigated (Fig. S62). As expected, the anti-node-substituted **1-N(Me)₂-Py** and **1-NO₂-Py** exhibit much higher product yields than their node-substituted counterparts. However, **2-N(Me)_2_-Py** and **2-NO_2_-Py** in PMMA exhibit observable RTP, which is not present in **1-N(Me)_2_-Py** and **1-NO_2_-Py**. Further, this framework on a different conjugated core was validated by synthesizing the phenanthrene–phenylacetylene derivatives **2-H-Phen** (nodal substitution) and **3-H-Phen** (anti-nodal substitution). Without doubt, **3-H-Phen** exhibits a higher product yield, whereas **2-H-Phen** in PMMA shows RTP intensity nearly 12-fold higher than **3-H-Phen** (Fig. S63). The detailed RTP QYs of the node-substituted compounds are summarized in Table S9, with calculated radiative and nonradiative decay rates. Note that the RTP QYs were estimated by applying Gaussian spectral deconvolution on the steady-state PL spectra (Fig. S64).

**Figure 6.**
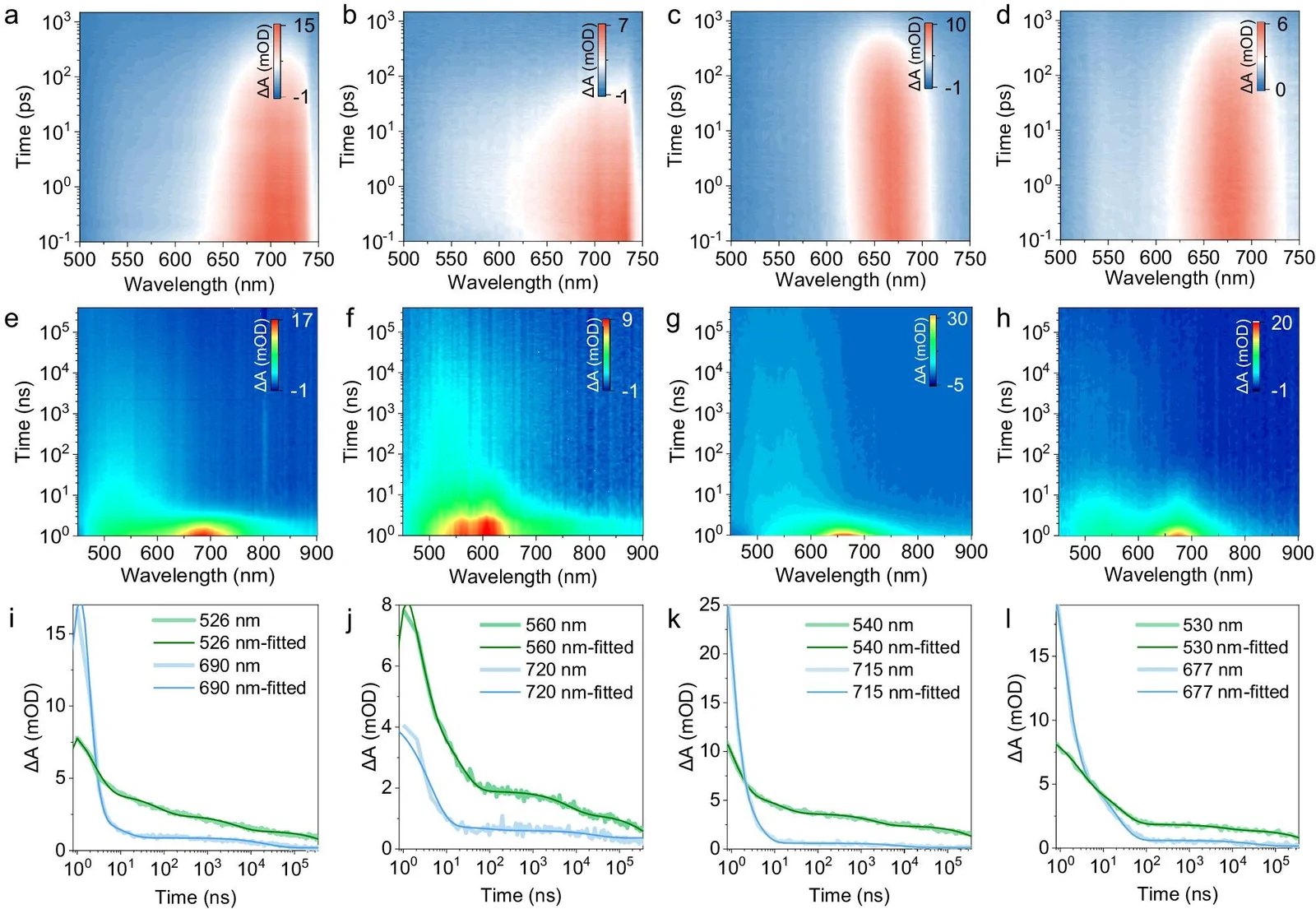
TA measurements of 1 wt% 1- and 2-substituted pyrenes in PMMA to elucidate exciton dynamics (*λ*_exc_ = 400 nm). (a–d) fs-TA intensity maps of (a) **1-H-Py**, (b) **2-H-Py**, (c) **1-CN-Py**, and (d) **2-CN-Py** in PMMA. (e–h) ns-TA intensity maps of (e) **1-H-Py**, (f) **2-H-Py**, (g) **1-CN-Py**, and (h) **2-CN-Py** in PMMA. (i–l) TA decay kinetics of (i) **1-H-Py**, (j) **2-H-Py**, (k) **1-CN-Py**, and (l) **2-CN-Py** in PMMA; note that though decay kinetics were not measured exactly at the absorption maxima, they reflect triplet-exciton dynamics of the pyrene core with and without CT-mediated ISC, because the two selected wavelengths have substantially minimized cross-interference among different triplet-exciton species.

Finally, time-resolved transient absorption (TA) spectroscopy techniques were employed to elucidate the excited-state dynamics of 1- and 2-substituted pyrenes, where **1-H-Py/2-H-Py** and **1-CN-Py/2-CN-Py** in PMMA were taken as examples for comparison. Upon excitation, all derivatives show positive S_*n*_←S_1_ absorption bands around 500–720 nm in fs-TA spectra, which have fast decay kinetics within several hundred picoseconds (Figs 6a–d and S65). Compared to 1-substituted pyrenes, **2-H-Py** and **2-CN-Py** have broader absorption bands with shoulder features at ca. 560 and 530 nm (Fig. S66), along with decay kinetics similar to those at 720 nm and 670 nm, respectively. This spectral broadening is attributed to the formation of CT states, matching the analyses of theoretical calculations and PL spectra, wherein nodal (2-position) substitution on the pyrene core promotes the formation of low-lying CT states that act as the bridge to enhance ISC and populate triplet excitons.

To probe the excited dynamics of triplet excitons, ns-TA spectra were then measured. **1-H-Py** and **1-CN-Py** have intense triplet absorptions at 685 and 661 nm (Fig. 6e and g), respectively, whose excitons decay within 10 ns (Fig. 6i and k). However, **2-H-Py** and **2-CN-Py** exhibit triplet absorptions mainly at 608 and 675 nm (Fig. 6f and h), respectively, with excitons persisting over 100 ns. This explains why 2-substituted pyrenes emit the bright RTP in PMMA, due to their long-lived triplet excitons. It should be noted that all **1-H-Py, 1-CN-Py, 2-H-Py** and **2-CN-Py** display weak yet long-lived absorption signals at 535–560 nm with similar exciton kinetics. These are attributed to the absorption of T_1_ excitons generated by the pyrene core itself without the perturbation of the substituents [61].

## CONCLUSION

In conclusion, we have established a clear structure–property relationship demonstrating how MO nodal phase regulates both ground-state chemical reactivity and excited-state dynamics in pyrene and phenanthrenes. Using Sonogashira coupling as the model reaction, we demonstrated that anti-nodal substitution delivers significantly higher cross-coupling yields under identical conditions, nearly twice those of nodal substitution. This can be rationalized by larger MO amplitude at the anti-nodal position that facilitates the bond formation. Integrated theoretical calculations and spectroscopic analyses further reveal that anti-nodal substitution extends π-conjugation and breaks symmetry, facilitating fluorescence while disfavoring ISC and triplet emission. By contrast, nodal substitution preserves frontier orbital symmetry and electronic decoupling at the junction, and promotes the formation of CT states that improve ISC and activate RTP. These findings provide insightful guidance on leveraging MO nodes to design functionalized luminophores with tailored chemical reactivity and photophysics, advancing versatile applications in optoelectronics, bioimaging, and information storage.

## MATERIALS AND METHODS

### Materials

1-Bromopyrene, 2-bromopyrene, phenylacetylene, *p*-methoxyphenylacetylene, *p*-cyanophenylacetylene, bis(triphenylphosphine)palladium(II) dichloride, copper(I) iodide, poly(methyl methacrylate) (average *M*w∼100 000), tetrahydrofuran, diisopropylamine, dichloromethane, petroleum ether, and acetonitrile were acquired from Energy Chemical Co., Ltd. and used as received. 2-Methyltetrahydrofuran was distilled prior to use.

### Structural characterization

NMR spectra were recorded on a Bruker AV400 NMR spectrometer operating in the Fourier transform mode (400 MHz for ^1^H and 101 MHz for ^13^C). NMR data are reported as chemical shift (δ, ppm), multiplicity (s = singlet, d = doublet, t = triplet, q = quartet, m = multiplet, br = broad), and coupling constant (*J*, Hz). Chemical shifts were referenced to tetramethylsilane (δ = 0.00 ppm) and are reported in the indicated deuterated solvent. High-resolution mass spectrometry was performed on a gas chromatography–Q Exactive Orbitrap mass spectrometer (GC–Q Exactive GC). High-performance liquid chromatography (HPLC) measurements were conducted on a Shimadzu Essentia LC-16 system using acetonitrile and water as the mobile phase. SCXRD was collected on a Bruker SMART APEXII CCD diffractometer using graphite-monochromated Cu Kα radiation (λ = 1.54178 Å).

### Photophysical investigations

UV–vis absorption spectra were recorded on a PerkinElmer Lambda 465 UV–Vis spectrometer. Steady-state excitation and emission spectra were recorded on a Horiba FluoroMax-4 spectrofluorometer (Horiba Scientific), equipped with a vertically mounted 150 W ozone-free continuous-wave xenon arc lamp. The distance between the excitation source and the sample was ca. 120 cm, and the integration time was 0.1 s. Delayed emission spectra were recorded on the same instrument using a 10-W xenon flash lamp as the excitation source.

Time-resolved PL decay measurements were carried out using time-correlated single-photon counting (TCSPC) and multichannel scaling (MCS) techniques. Fluorescence lifetimes were excited with a NanoLED-370 (peak wavelength: 372 nm), and phosphorescence lifetimes were recorded with a SpectraLED-280 (peak wavelength: 285 nm). Lifetime data were analyzed using Data Station v6.6 (Horiba Scientific). Time-resolved PL measurements were fitted to a sum of exponential decay models, with chi-squared (χ^2^) values between 1 and 1.1.

Femtosecond transient absorption (fs-TA) spectra were measured using a home-built fs pump−probe setup. The laser pulse (800 nm, 35 fs pulse width, 1 kHz repetition rate) was generated by a regeneratively amplified Ti: sapphire laser system (Coherent Astrella-Tunable-USP, USA). The output of the pulse was split into pump and probe beams with a beam splitter. The 400 nm pump pulse was produced by frequency doubling 90% of 800 nm beam using a β-barium borate crystal (type I, 0.5 mm thickness). The energy of the pump pulse was about 0.1 μJ. The probe beam was delayed with a computer-controlled optical delay line and then focused on a thin sapphire plate to generate the white-light supercontinuum, which was consequently split into the signal and reference beams (450–800 nm) using a broadband 50/50 beam splitter. The focused pump and probe pulses were spatially overlapped into a sample cuvette or on a film sample. The mutual polarization between the pump and probe beams was set to the magic angle (54.7°) using a half-wave plate in the pump path.

Nanosecond transient absorption (ns-TA) spectra were measured by a commercial spectrometer (Time-Tech Spectra). The pump generation was identical to that used for fs-TA. The probe beam was generated from a supercontinuum laser (LEUKOS-DISCO, France) with the spectral range from 350 to 1800 nm. The repetition rate was 2 kHz and pulse width was 700 ps–1 ns.

### Theoretical calculations

Density functional theory (DFT) calculations were performed with the Gaussian 16 program [62]. MO analysis and visualization were carried out using Multiwfn 3.8 (dev) [63,64] and VMD 1.9.3 [65]. Ground-state (S_0_) geometries were optimized at the B3LYP [56,66]/6-31G(d) [57] level. Triplet (T_1_) geometries were optimized using unrestricted DFT at the same level. Vibrational frequency calculations were performed to confirm that the optimized geometries correspond to local minima (no imaginary frequencies). Time-dependent DFT (TD-DFT) calculations within Tamm–Dancoff approximation (TDA) were conducted using the range-separated hybrid functional ωB97XD [67] with the high-precision 3-ζ def2TZVP [68] basis set, including 10 singlet and 10 triplet states. Spin-orbit coupling (SOC) matrix elements were computed using ORCA 6.1.0 at the same level. Reaction energies were estimated from the difference between the energy minima of the initial state (reactants) and the final state (products). The transition state was identified by the presence of a single imaginary frequency.

## Supplementary Material

nwag394_Supplemental_Files

## Data Availability

All data needed to support the conclusions of this manuscript are included in the main text and/or the supplemental information. The crystallographic data for **1-H-Py** and **2-H-Py** have been deposited in the Cambridge Crystallographic Data Center (CCDC; https://www.ccdc.cam.ac.uk/structures/) under accession numbers CCDC: 2512220 and 2512221.
