# Supplementary material for: Molecular orbital node engineering in pyrene: linking chemical reactivity with room-temperature phosphorescence
Source: Natl Sci Rev. 2026 Jun 26;13(17):nwag394. doi: 10.1093/nsr/nwag394 (PMC13570590; doi:10.1093/nsr/nwag394)

checkCIF/PLATON report

You have not supplied any structure factors. As a result the full set of tests cannot be run.

THIS REPORT IS FOR GUIDANCE ONLY. IF USED AS PART OF A REVIEW PROCEDURE FOR PUBLICATION, IT SHOULD NOT REPLACE THE EXPERTISE OF AN EXPERIENCED CRYSTALLOGRAPHIC REFEREE.

No syntax errors found. CIF dictionary Interpreting this report

Datablock: zxl-1

```
Bond precision:   C-C = 0.0040 A          Wavelength=1.54184

Cell:              a=22.4908(7)       b=14.6685(5)       c=4.8593(2)
                   alpha=90           beta=90            gamma=90
Temperature:       301 K

                Calculated                  Reported
Volume          1603.11(10)                 1603.11(10)
Space group     P n a 21                    P n a 21
Hall group      P 2c -2n                    P 2c -2n
Moiety formula  C24 H14                     C24 H14
Sum formula     C24 H14                     C24 H14
Mr              302.35                      302.35
Dx,g cm-3       1.253                       1.253
Z               4                           4
Mu (mm-1)       0.541                       0.541
F000            632.0                       632.0
F000'           633.66
h,k,lmax        28,18,6                     28,18,6
Nref            3481[ 1951]                 2884
Tmin,Tmax       0.771,0.850                 0.737,1.000
Tmin'           0.723

Correction method= # Reported T Limits: Tmin=0.737 Tmax=1.000
AbsCorr = MULTI-SCAN
```

Data completeness= 1.48/0.83   Theta(max)= 79.311

R(reflections)= 0.0422(2068)   wR2(reflections)= 0.1165(2884)

S = 1.005   Npar= 217

The following ALERTS were generated. Each ALERT has the format
test-name_ALERT_alert-type_alert-level.
Click on the hyperlinks for more details of the test.

Alert level C

```
CRYSC01_ALERT_1_C No recognised colour has been given for crystal colour.
STRVA01_ALERT_4_C           Flack test results are ambiguous.
           From the CIF: _refine_ls_abs_structure_Flack    0.400
           From the CIF: _refine_ls_abs_structure_Flack_su    1.000
PLAT230_ALERT_2_C Hirshfeld Test Diff for    C00L     --C00N     .        6.8 s.u.
PLAT340_ALERT_3_C Low Bond Precision on  C-C Bonds ..............    0.00404 Ang.
PLAT790_ALERT_4_C Centre of Gravity not Within Unit-Cell: Resd.  #          1 Note
              C24 H14
```

Alert level G

```
PLAT032_ALERT_4_G Std. Uncertainty on Flack Parameter Value High .      1.000 Report
PLAT371_ALERT_2_G Long   C(sp2)-C(sp1) Bond  C005     - C00F     .       1.43 Ang.
PLAT371_ALERT_2_G Long   C(sp2)-C(sp1) Bond  C009     - C00J     .       1.44 Ang.
PLAT720_ALERT_4_G Number of Unusual/Non-Standard Labels .........          38 Note
              C001    C002    C003    C004    C005    C006    C007    H007
              C008    C009    C00A    H00A    C00B    H00B    C00C    H00C
              C00D    H00D    C00E    H00E    C00F    C00G    H00G    C00H
              H00H    C00I    H00I    C00J    C00K    H00K    C00L    H00L
              C00M    H00M    C00N    H00N    C00O    H00O
PLAT933_ALERT_2_G Number of HKL-OMIT Records in Embedded .res File          33 Note
               18  5  4,  15  8  4,  15  9  3,  17  7  3,  13  9  4,  20  9 -3,
               17  6  4,  16  8  3,  19  5  3,  16  7  4,  18  6  3,  13 10  3,
               11  8  5,  14  8  4,  15  8  2,  10 11  3,  16  6  4,   9 15 -3,
               14  9  3,  16  8  2,  21  8 -3,  12 10  3,  12 10  2,   8 11  2,
               15  7  4,  13  8  4,  15  8  3,  15  4  5,  19 10 -3,  18 11 -3,
               13  6  5,   1  8  0,  18  4  4,
PLAT941_ALERT_3_G Average HKL Measurement Multiplicity ..........        3.2 Low
```

---

0 **ALERT level A** = Most likely a serious problem - resolve or explain
0 **ALERT level B** = A potentially serious problem, consider carefully
5 **ALERT level C** = Check. Ensure it is not caused by an omission or oversight
6 **ALERT level G** = General information/check it is not something unexpected

1 ALERT type 1 CIF construction/syntax error, inconsistent or missing data
4 ALERT type 2 Indicator that the structure model may be wrong or deficient
2 ALERT type 3 Indicator that the structure quality may be low
4 ALERT type 4 Improvement, methodology, query or suggestion
0 ALERT type 5 Informative message, check

---

checkCIF publication errors

Alert level A

```
PUBL004_ALERT_1_A  The contact author's name and address are missing,
            _publ_contact_author_name and _publ_contact_author_address.
PUBL005_ALERT_1_A  _publ_contact_author_email, _publ_contact_author_fax and
            _publ_contact_author_phone are all missing.
            At least one of these should be present.
PUBL006_ALERT_1_A  _publ_requested_journal is missing
            e.g. 'Acta Crystallographica Section C'
PUBL008_ALERT_1_A  _publ_section_title is missing. Title of paper.
PUBL009_ALERT_1_A  _publ_author_name is missing. List of author(s) name(s).
PUBL010_ALERT_1_A  _publ_author_address is missing. Author(s) address(es).
PUBL012_ALERT_1_A  _publ_section_abstract is missing.
            Abstract of paper in English.
```

---

7 **ALERT level A** = Data missing that is essential or data in wrong format
0 **ALERT level G** = General alerts. Data that may be required is missing

---

Publication of your CIF

You should attempt to resolve as many as possible of the alerts in all categories. Often the minor alerts point to easily fixed oversights, errors and omissions in your CIF or refinement strategy, so attention to these fine details can be worthwhile. In order to resolve some of the more serious problems it may be necessary to carry out additional measurements or structure refinements. However, the nature of your study may justify the reported deviations from journal submission requirements and the more serious of these should be commented upon in the discussion or experimental section of a paper or in the "special_details" fields of the CIF. *checkCIF* was carefully designed to identify outliers and unusual parameters, but every test has its limitations and alerts that are not important in a particular case may appear. Conversely, the absence of alerts does not guarantee there are no aspects of the results needing attention. It is up to the individual to critically assess their own results and, if necessary, seek expert advice.

If level A alerts remain, which you believe to be justified deviations, and you intend to submit this CIF for publication in a journal, you should additionally insert an explanation in your CIF using the Validation Reply Form (VRF) below. This will allow your explanation to be considered as part of the review process.

Validation response form

Please find below a validation response form (VRF) that can be filled in and pasted into your CIF.

```
# start Validation Reply Form
_vrf_PUBL004_GLOBAL
;
PROBLEM: The contact author's name and address are missing,
RESPONSE: ...
```

```
;
_vrf_PUBL005_GLOBAL
;
PROBLEM: _publ_contact_author_email, _publ_contact_author_fax and
RESPONSE: ...
;
_vrf_PUBL006_GLOBAL
;
PROBLEM: _publ_requested_journal is missing
RESPONSE: ...
;
_vrf_PUBL008_GLOBAL
;
PROBLEM: _publ_section_title is missing. Title of paper.
RESPONSE: ...
;
_vrf_PUBL009_GLOBAL
;
PROBLEM: _publ_author_name is missing. List of author(s) name(s).
RESPONSE: ...
;
_vrf_PUBL010_GLOBAL
;
PROBLEM: _publ_author_address is missing. Author(s) address(es).
RESPONSE: ...
;
_vrf_PUBL012_GLOBAL
;
PROBLEM: _publ_section_abstract is missing.
RESPONSE: ...
;
# end Validation Reply Form
```

If you wish to submit your CIF for publication in Acta Crystallographica Section C or E, you should upload your CIF via the web. If you wish to submit your CIF for publication in IUCrData you should upload your CIF via the web. If your CIF is to form part of a submission to another IUCr journal, you will be asked, either during electronic submission or by the Co-editor handling your paper, to upload your CIF via our web site.

PLATON version of 26/09/2025; check.def file version of 20/09/2025

Datablock zxl-1 - ellipsoid plot

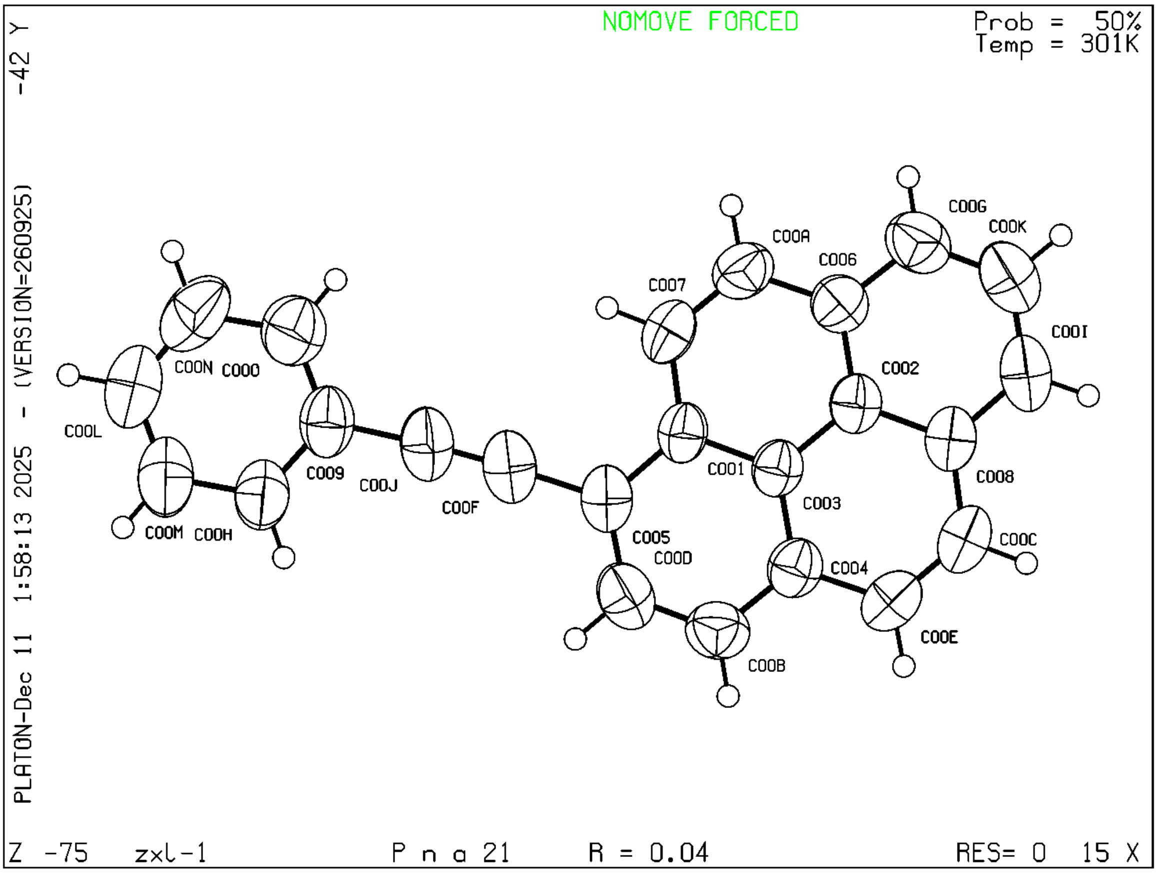

Supplement: Supplementary file 1 [file nwag394_supplemental_files.zip › Checkcif file of 1-H-Py.pdf]
