# Supplementary material for: Molecular orbital node engineering in pyrene: linking chemical reactivity with room-temperature phosphorescence
Source: Natl Sci Rev. 2026 Jun 26;13(17):nwag394. doi: 10.1093/nsr/nwag394 (PMC13570590; doi:10.1093/nsr/nwag394)

checkCIF/PLATON report

You have not supplied any structure factors. As a result the full set of tests cannot be run.

THIS REPORT IS FOR GUIDANCE ONLY. IF USED AS PART OF A REVIEW PROCEDURE FOR PUBLICATION, IT SHOULD NOT REPLACE THE EXPERTISE OF AN EXPERIENCED CRYSTALLOGRAPHIC REFEREE.

No syntax errors found. CIF dictionary Interpreting this report

Datablock: zxl-3

```
Bond precision:   C-C = 0.0019 A          Wavelength=1.54184

Cell:            a=8.0731(1)       b=8.1138(1)       c=24.6106(4)
                 alpha=89.493(1)   beta=89.429(1)    gamma=79.638(1)
Temperature:     301 K

                  Calculated                    Reported
Volume            1585.67(4)                    1585.67(4)
Space group       P -1                          P -1
Hall group        -P 1                          -P 1
Moiety formula    C24 H14                       C24 H14
Sum formula       C24 H14                       C24 H12
Mr                302.35                        300.34
Dx,g cm-3         1.266                         1.258
Z                 4                             4
Mu (mm-1)         0.547                         0.546
F000              632.0                         624.0
F000'             633.66
h,k,lmax          10,10,31                      10,10,31
Nref              6892                          6626
Tmin,Tmax         0.822,0.849                   0.506,1.000
Tmin'             0.721

Correction method= # Reported T Limits: Tmin=0.506 Tmax=1.000
AbsCorr = NONE
```

Data completeness= 0.961 Theta(max)= 79.361

R(reflections)= 0.0713(5446) wR2(reflections)= 0.1990(6626)

S = 1.051 Npar= 433

The following ALERTS were generated. Each ALERT has the format
test-name_ALERT_alert-type_alert-level.
Click on the hyperlinks for more details of the test.

Alert level C

```
CRYSC01_ALERT_1_C No recognised colour has been given for crystal colour.
RINTA01_ALERT_3_C  The value of Rint is greater than 0.12
            Rint given   0.144
PLAT020_ALERT_3_C The Value of Rint is Greater Than 0.12 .........      0.144 Report
PLAT041_ALERT_1_C Calc. and Reported SumFormula    Strings  Differ     Please Check
              Calc: C24 H14
              Rep.: C24 H12
PLAT043_ALERT_1_C Calculated and Reported Mol. Weight Differ by ..       2.01 Check
PLAT068_ALERT_1_C Reported F000 Differs from Calcd (or Missing)...     Please Check
PLAT230_ALERT_2_C Hirshfeld Test Diff for    C016     --C01A     .        5.7 s.u.
PLAT230_ALERT_2_C Hirshfeld Test Diff for    C013     --C015     .        6.4 s.u.
```

Alert level G

```
FORMU01_ALERT_1_G  There is a discrepancy between the atom counts in the
            _chemical_formula_sum and _chemical_formula_moiety. This is
            usually due to the moiety formula being in the wrong format.
            Atom count from _chemical_formula_sum:   C24 H12
            Atom count from _chemical_formula_moiety:C24 H14
FORMU01_ALERT_2_G  There is a discrepancy between the atom counts in the
            _chemical_formula_sum and the formula from the _atom_site* data.
            Atom count from _chemical_formula_sum:C24 H12
            Atom count from the _atom_site data:  C24 H14
CELLZ01_ALERT_1_G Difference between formula and atom_site contents detected.
CELLZ01_ALERT_1_G ALERT: Large difference may be due to a
            symmetry error - see SYMMG tests
           From the CIF: _cell_formula_units_Z    4
           From the CIF: _chemical_formula_sum  C24 H12
           TEST: Compare cell contents of formula and atom_site data

           atom    Z*formula  cif sites diff
           C         96.00     96.00    0.00
           H         48.00     56.00   -8.00
PLAT072_ALERT_2_G SHELXL First  Parameter in WGHT  Unusually Large       0.14 Report
PLAT154_ALERT_1_G The s.u.'s on the Cell Angles are Equal ..(Note)      0.001 Degree
PLAT371_ALERT_2_G Long   C(sp2)-C(sp1) Bond  C005     - C00I     .       1.43 Ang.
PLAT371_ALERT_2_G Long   C(sp2)-C(sp1) Bond  C00G     - C00J     .       1.43 Ang.
PLAT371_ALERT_2_G Long   C(sp2)-C(sp1) Bond  C00K     - C00X     .       1.43 Ang.
PLAT371_ALERT_2_G Long   C(sp2)-C(sp1) Bond  C00L     - C00W     .       1.43 Ang.
PLAT720_ALERT_4_G Number of Unusual/Non-Standard Labels .........          76 Note
              C001    C002    C003    C004    C005    C006    C007    C008
              H008    C009    H009    C00A    C00B    H00B    C00C    C00D
```

```
                C00E    H00E    C00F    H00F    C00G    C00H    H00H    C00I
                C00J    C00K    C00L    C00M    H00M    C00N    H00N    C00O
                C00P    H00P    C00Q    C00R    H00R    C00S    H00S    C00T
                C00U    H00U    C00V    H00V    C00W    C00X    C00Y    H00Y
                C00Z    H00Z    C010    H010    C011    H011    C012    H012
                C013    H013    C014    H014    C015    H015    C016    H016
                C017    H017    C018    H018    C019    H019    C01A    H01A
                C01B    H01B    C01C    H01C
PLAT933_ALERT_2_G Number of HKL-OMIT Records in Embedded .res File         62 Note
                 10  3  2,  -2 -4  9,   6  3  8,  -6 -2  5,   9  4  9,  -9 -6  6,
                  4  0  6,   7  4  6,   9  6  1,  -1 -1  1, -10 -1  1,  -1 -5  4,
                -10 -2  2,  -5 -3  4,   9  6  5,  -3  3 16,  -9 -6  5,  -2 -5  7,
                 -7 -3  7, -10 -3  5,   3 -5 23, -10 -2  5,  -2 -4  1,  -5 -2  4,
                  9  6  2,   9  6  3,  -2 -3  1,   0  8 16,   3  2  9,   1 -3  9,
                  1 -1  5,   2 -2  2,   7  7 13,  -5 -3  2,   2 -2 21,  -5  1  1,
                 -2 -4  8,  -6  3 21,   5  4  7,  10  1  4,   4 -2 10,  -6 -3  5,
                  3 -3 18,  -9 -6  4,  -5 -2  6,   2  0  4,   3  0  4,  -6 -1  1,
                -10 -3  2,   2 -1  4,
PLAT941_ALERT_3_G Average HKL Measurement Multiplicity ..........        3.1 Low
```

```
   0 ALERT level A = Most likely a serious problem - resolve or explain
   0 ALERT level B = A potentially serious problem, consider carefully
   8 ALERT level C = Check. Ensure it is not caused by an omission or oversight
  13 ALERT level G = General information/check it is not something unexpected

   8 ALERT type 1 CIF construction/syntax error, inconsistent or missing data
   9 ALERT type 2 Indicator that the structure model may be wrong or deficient
   3 ALERT type 3 Indicator that the structure quality may be low
   1 ALERT type 4 Improvement, methodology, query or suggestion
   0 ALERT type 5 Informative message, check
```

---

checkCIF publication errors

Alert level A

```
PUBL004_ALERT_1_A  The contact author's name and address are missing,
            _publ_contact_author_name and _publ_contact_author_address.
PUBL005_ALERT_1_A  _publ_contact_author_email, _publ_contact_author_fax and
            _publ_contact_author_phone are all missing.
            At least one of these should be present.
PUBL006_ALERT_1_A  _publ_requested_journal is missing
            e.g. 'Acta Crystallographica Section C'
PUBL008_ALERT_1_A  _publ_section_title is missing. Title of paper.
PUBL009_ALERT_1_A  _publ_author_name is missing. List of author(s) name(s).
PUBL010_ALERT_1_A  _publ_author_address is missing. Author(s) address(es).
PUBL012_ALERT_1_A  _publ_section_abstract is missing.
            Abstract of paper in English.
```

```
   7 ALERT level A = Data missing that is essential or data in wrong format
   0 ALERT level G = General alerts. Data that may be required is missing
```

Publication of your CIF

You should attempt to resolve as many as possible of the alerts in all categories. Often the minor alerts point to easily fixed oversights, errors and omissions in your CIF or refinement strategy, so attention to these fine details can be worthwhile. In order to resolve some of the more serious problems it may be necessary to carry out additional measurements or structure refinements. However, the nature of your study may justify the reported deviations from journal submission requirements and the more serious of these should be commented upon in the discussion or experimental section of a paper or in the "special_details" fields of the CIF. *checkCIF* was carefully designed to identify outliers and unusual parameters, but every test has its limitations and alerts that are not important in a particular case may appear. Conversely, the absence of alerts does not guarantee there are no aspects of the results needing attention. It is up to the individual to critically assess their own results and, if necessary, seek expert advice.

If level A alerts remain, which you believe to be justified deviations, and you intend to submit this CIF for publication in a journal, you should additionally insert an explanation in your CIF using the Validation Reply Form (VRF) below. This will allow your explanation to be considered as part of the review process.

Validation response form

Please find below a validation response form (VRF) that can be filled in and pasted into your CIF.

```
# start Validation Reply Form
_vrf_PUBL004_GLOBAL
;
PROBLEM: The contact author's name and address are missing,
RESPONSE: ...
;
_vrf_PUBL005_GLOBAL
;
PROBLEM: _publ_contact_author_email, _publ_contact_author_fax and
RESPONSE: ...
;
_vrf_PUBL006_GLOBAL
;
PROBLEM: _publ_requested_journal is missing
RESPONSE: ...
;
_vrf_PUBL008_GLOBAL
;
PROBLEM: _publ_section_title is missing. Title of paper.
RESPONSE: ...
;
_vrf_PUBL009_GLOBAL
;
PROBLEM: _publ_author_name is missing. List of author(s) name(s).
RESPONSE: ...
;
_vrf_PUBL010_GLOBAL
;
PROBLEM: _publ_author_address is missing. Author(s) address(es).
```

```
RESPONSE: ...
;
_vrf_PUBL012_GLOBAL
;
PROBLEM: _publ_section_abstract is missing.
RESPONSE: ...
;
# end Validation Reply Form
```

If you wish to submit your CIF for publication in Acta Crystallographica Section C or E, you should upload your CIF via the web. If you wish to submit your CIF for publication in IUCrData you should upload your CIF via the web. If your CIF is to form part of a submission to another IUCr journal, you will be asked, either during electronic submission or by the Co-editor handling your paper, to upload your CIF via our web site.

PLATON version of 26/09/2025; check.def file version of 20/09/2025

Datablock zxl-3 - ellipsoid plot

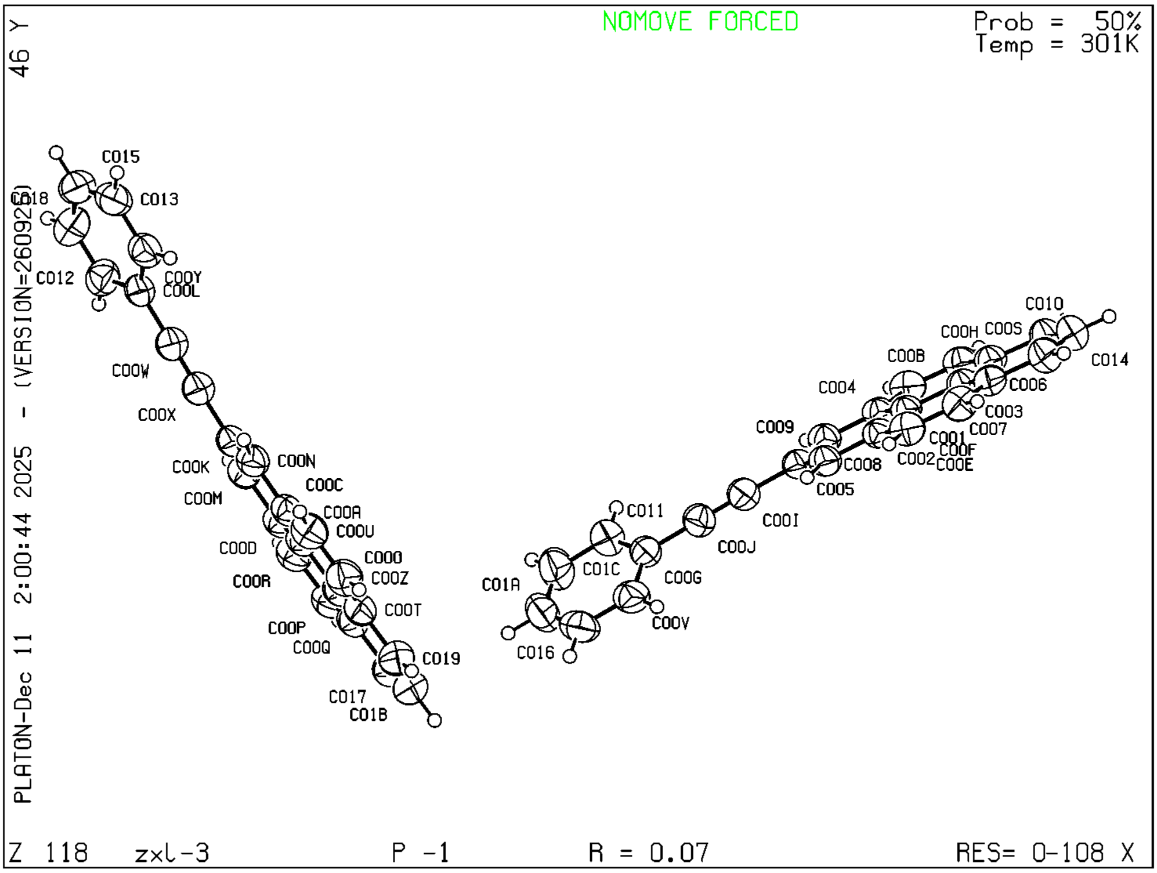

Supplement: Supplementary file 1 [file nwag394_supplemental_files.zip › Checkcif file of 2-H-Py.pdf]
