# Supplementary material for: Molecular orbital node engineering in pyrene: linking chemical reactivity with room-temperature phosphorescence
Source: Natl Sci Rev. 2026 Jun 26;13(17):nwag394. doi: 10.1093/nsr/nwag394 (PMC13570590; doi:10.1093/nsr/nwag394)
Supplement: Supplementary file 1 [file nwag394_supplemental_files.zip › Supporting Information.pdf]

Supplemental Information

Molecular Orbital Node Engineering in Pyrene: Linking Chemical Reactivity with Room-Temperature Phosphorescence

Hongping Liu†, Dingcheng Zhou†, Wei Zhang†, Xiaolong Zhang, Chengze Yang, Baicheng Zhang, Aoyuan Cheng, Hao Su,Yang Zhang, Meng Zhou*, Xuepeng Zhang*, Tao Wang* and Guoqing Zhang*

Table of Contents

Part I. Materials, Synthesis Procedures and Preparation of Samples 3
Synthesis 3
Synthesis of 1-H-Py 3
Synthesis of 1-OME-Py 3
Synthesis of 1-CN-Py 4
Synthesis of 2-H-Py 4
Synthesis of 2-OME-Py 5
Synthesis of 2-CN-Py 5
Synthesis of 4-H-Py 6
Synthesis of 1-NMe2-Py 6
Synthesis of 2-NMe_2-Py 7
Synthesis of 1-NO_2-Py 7
Synthesis of 2-NO_2-Py 8
Synthesis of 2-H-Phen 8
Synthesis of 3-H-Phen 9
Preparation of polymer films 9
Preparation of single crystal samples 9
Part II. Structural characterization 10
Part III. Other Supplementary Figures 55
Theoretical calculations 55
Spectral data in solution 58
Spectral data in PMMA 67
Ultrafast spectroscopy 74
Part IV. Other Supplementary Tables 76

Part I. Materials, Synthesis Procedures and Preparation of Samples

Synthesis

Synthesis of 1-H-Py

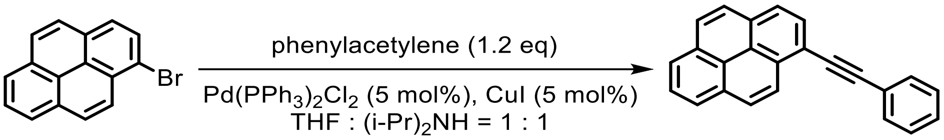

Scheme S1. Synthetic route and chemical structure of **1-H-Py**.

1-Bromopyrene (0.60 g, 2.10 mmol), bis(triphenylphosphine)palladium(II) dichloride (0.075 g, 0.10 mmol), and copper(I) iodide (0.020 g, 0.10 mmol) were added to a flask. Phenylacetylene (0.25 g, 2.50 mmol) was dissolved in tetrahydrofuran (3 mL) and added, followed by tetrahydrofuran (7 mL) and diisopropylamine (10 mL). The mixture was stirred under nitrogen at 80 °C for 72 h and quenched with dichloromethane. The product was purified by column chromatography (petroleum ether/dichloromethane = 3:1) to afford 0.496 g (76.8%) of the target compound. **^{1}H NMR** (400 MHz, Chloroform-*d*) δ (ppm): 8.67 (d, J = 9.1 Hz, 1H), 8.26–8.17 (m, 4H), 8.15–8.08 (m, 2H), 8.07–8.00 (m, 2H), 7.75–7.70 (m, 2H), 7.47–7.37 (m, 3H). **^{13}C NMR** (101 MHz, Chloroform-*d*) δ (ppm): 131.92, 131.73, 131.27, 131.10, 129.64, 128.53, 128.45, 128.36, 128.18, 127.29, 126.27, 125.66, 125.61, 125.58, 124.57, 124.52, 124.35, 123.54, 117.81, 95.13, 88.65. **HRMS** (EI), m/z calcd for $C_{24}H_{14}$: 302.10955 $[M]^{+\cdot}$, found: 302.10850.

Synthesis of 1-OME-Py

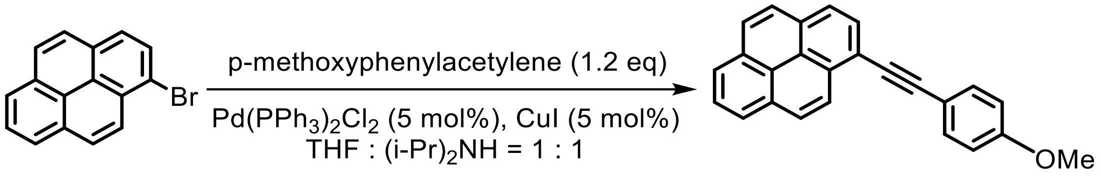

Scheme S2. Synthetic route and chemical structure of **1-OME-Py**.

1-Bromopyrene (0.60 g, 2.10 mmol), bis(triphenylphosphine)palladium(II) dichloride (0.075 g, 0.10 mmol), and copper(I) iodide (0.020 g, 0.10 mmol) were added to a flask. p-Methoxyphenylacetylene (0.33 g, 2.50 mmol) was dissolved in tetrahydrofuran (3 mL) and added, followed by tetrahydrofuran (7 mL) and diisopropylamine (10 mL). The mixture was stirred under nitrogen at 80 °C for 72 h and quenched with dichloromethane. The product was purified by column chromatography (petroleum ether/dichloromethane = 3:1) to afford 0.606 g (85.4%) of the target compound. **^{1}H NMR** (400 MHz, Chloroform-*d*) δ (ppm): 8.66 (d, J = 9.1 Hz, 1H), 8.25–8.15 (m, 4H), 8.14–7.99 (m, 4H), 7.69–7.63 (m, 2H), 7.00–6.93 (m, 2H), 3.87 (s, 3H). **^{13}C NMR** (101 MHz, Chloroform-*d*) δ (ppm): 159.80, 133.19, 131.74, 131.31, 131.14, 131.02, 129.47, 128.20, 127.98, 127.30, 126.22, 125.67, 125.53, 125.49, 124.56, 124.40, 118.26, 115.67, 114.16, 95.22, 87.37, 55.39. **HRMS** (EI), m/z calcd for $C_{25}H_{16}O$: 332.12012 $[M]^{+\cdot}$, found: 332.11907.

Synthesis of 1-CN-Py

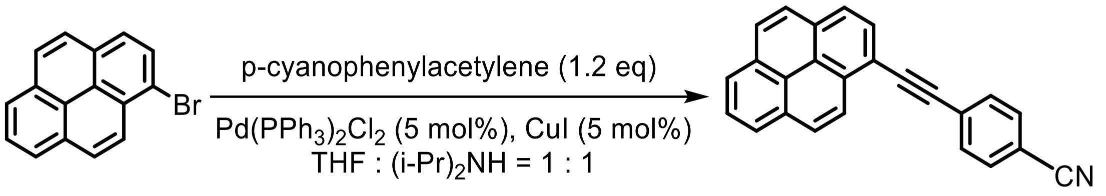

Scheme S3. Synthetic route and chemical structure of **1-CN-Py**.

1-Bromopyrene (0.60 g, 2.10 mmol), bis(triphenylphosphine)palladium(II) dichloride (0.075 g, 0.10 mmol), and copper(I) iodide (0.020 g, 0.10 mmol) were added to a flask. p-Cyanophenylacetylene (0.32 g, 2.50 mmol) was dissolved in tetrahydrofuran (3 mL) and added, followed by tetrahydrofuran (7 mL) and diisopropylamine (10 mL). The mixture was stirred under nitrogen at 80 °C for 72 h and quenched with dichloromethane. The product was purified by column chromatography (petroleum ether/dichloromethane = 3:1) to afford 0.439 g (62.8%) of the target compound. **^{1}H NMR** (400 MHz, Chloroform-*d*) δ (ppm): 8.58 (d, J = 9.1 Hz, 1H), 8.27–8.17 (m, 4H), 8.15–8.09 (m, 2H), 8.07–8.01 (m, 2H), 7.77–7.63 (m, 4H). **^{13}C NMR** (101 MHz, Chloroform-*d*) δ (ppm): 132.17, 132.09, 131.92, 131.21, 130.98, 129.82, 128.79, 128.72, 128.46, 127.23, 126.45, 125.98, 125.16, 124.60, 124.46, 124.23, 118.63, 116.48, 111.46, 93.36, 93.21. **HRMS** (EI), m/z calcd for $C_{25}H_{13}N$: 327.10480 $[M]^{+\cdot}$, found: 327.10389.

Synthesis of 2-H-Py

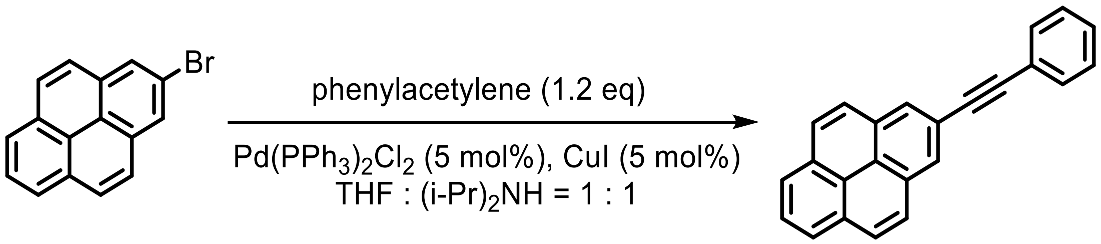

Scheme S4. Synthetic route and chemical structure of **2-H-Py**.

2-Bromopyrene (0.60 g, 2.10 mmol), bis(triphenylphosphine)palladium(II) dichloride (0.075 g, 0.10 mmol), and copper(I) iodide (0.020 g, 0.10 mmol) were added to a flask. Phenylacetylene (0.25 g, 2.50 mmol) was dissolved in tetrahydrofuran (3 mL) and added, followed by tetrahydrofuran (7 mL) and diisopropylamine (10 mL). The mixture was stirred under nitrogen at 80 °C for 72 h and quenched with dichloromethane. The product was purified by column chromatography (petroleum ether/dichloromethane = 3:1) to afford 0.236 g (36.5%) of the target compound. **^{1}H NMR** (400 MHz, Chloroform-*d*) δ (ppm): 8.34 (s, 2H), 8.19 (d, J = 7.6 Hz, 2H), 8.11–7.98 (m, 5H), 7.68–7.62 (m, 2H), 7.44–7.35 (m, 3H). **^{13}C NMR** (101 MHz, Chloroform-*d*) δ (ppm): 131.77, 131.25, 131.11, 128.48, 128.43, 128.10, 127.76, 126.97, 126.35, 125.40, 124.40, 124.23, 123.32, 120.65, 90.07, 89.82. **HRMS** (EI), m/z calcd for $C_{24}H_{14}$: 302.10955 $[M]^{+\cdot}$, found: 302.10858.

Synthesis of 2-OME-Py

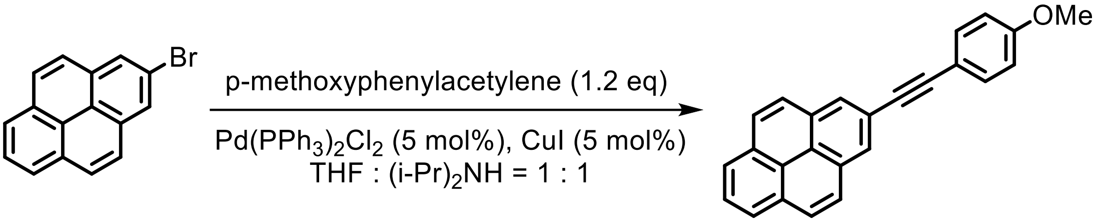

Scheme S5. Synthetic route and chemical structure of **2-OME-Py**.

2-Bromopyrene (0.60 g, 2.10 mmol), bis(triphenylphosphine)palladium(II) dichloride (0.075 g, 0.10 mmol), and copper(I) iodide (0.020 g, 0.10 mmol) were added to a flask. p-Methoxyphenylacetylene (0.33 g, 2.50 mmol) was dissolved in tetrahydrofuran (3 mL) and added, followed by tetrahydrofuran (7 mL) and diisopropylamine (10 mL). The mixture was stirred under nitrogen at 80 °C for 72 h and quenched with dichloromethane. The product was purified by column chromatography (petroleum ether/dichloromethane = 3:1) to afford 0.318 g (44.8%) of the target compound. **^{1}H NMR** (400 MHz, Chloroform-*d*) δ (ppm): 8.32 (d, J = 1.1 Hz, 2H), 8.18 (dd, J = 7.7, 1.2 Hz, 2H), 8.11–7.97 (m, 5H), 7.63–7.55 (m, 2H), 6.97–6.90 (m, 2H), 3.86 (s, 3H). **^{13}C NMR** (101 MHz, Chloroform-*d*) δ (ppm): 159.75, 133.22, 131.22, 131.11, 128.03, 127.63, 126.98, 126.26, 125.34, 124.44, 124.08, 121.04, 115.42, 114.11, 89.87, 88.76, 55.36. **HRMS** (EI), m/z calcd for $C_{25}H_{16}O$: 332.12012 $[M]^{+\cdot}$, found: 332.11966.

Synthesis of 2-CN-Py

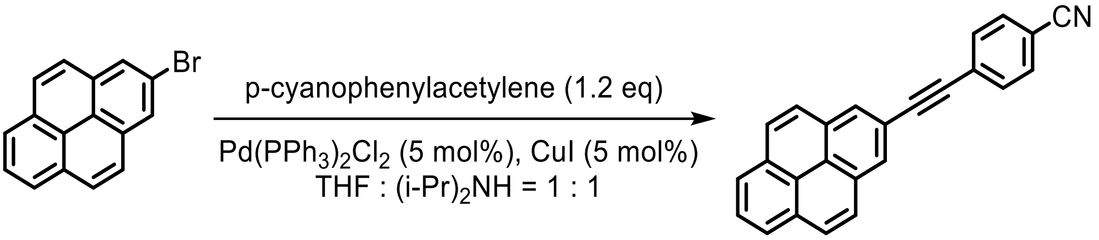

Scheme S6. Synthetic route and chemical structure of **2-CN-Py**.

2-Bromopyrene (0.60 g, 2.10 mmol), bis(triphenylphosphine)palladium(II) dichloride (0.075 g, 0.10 mmol), and copper(I) iodide (0.020 g, 0.10 mmol) were added to a flask. p-Cyanophenylacetylene (0.32 g, 2.50 mmol) was dissolved in tetrahydrofuran (3 mL) and added, followed by tetrahydrofuran (7 mL) and diisopropylamine (10 mL). The mixture was stirred under nitrogen at 80 °C for 72 h and quenched with dichloromethane. The product was purified by column chromatography (petroleum ether/dichloromethane = 3:1) to afford 0.240 g (34.3%) of the target compound. **^{1}H NMR** (400 MHz, Chloroform-*d*) δ (ppm): 8.34 (s, 2H), 8.20 (d, J = 7.6 Hz, 2H), 8.11 (d, J = 9.0 Hz, 2H), 8.07–8.01 (m, 3H), 7.73–7.65 (m, 4H). **^{13}C NMR** (101 MHz, Chloroform-*d*) δ (ppm): 132.18, 132.14, 131.33, 131.17, 128.36, 128.31, 127.79, 127.08, 126.87, 126.63, 125.58, 124.64, 124.31, 119.48, 118.59, 111.55, 94.54, 88.10. **HRMS** (EI), m/z calcd for $C_{25}H_{13}N$: 327.10480 $[M]^{+\cdot}$, found: 327.10446.

Synthesis of 4-H-Py

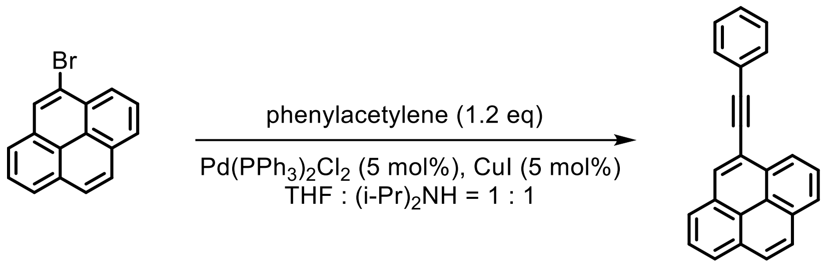

Scheme S7. Synthetic route and chemical structure of **4-H-Py**.

4-Bromopyrene (0.60 g, 2.10 mmol), bis(triphenylphosphine)palladium(II) dichloride (0.075 g, 0.10 mmol), and copper(I) iodide (0.020 g, 0.10 mmol) were added to a flask. Phenylacetylene (0.25 g, 2.50 mmol) was dissolved in tetrahydrofuran (3 mL) and added, followed by tetrahydrofuran (7 mL) and diisopropylamine (10 mL). The mixture was stirred under nitrogen at 80 °C for 72 h and quenched with dichloromethane. The product was purified by column chromatography (petroleum ether/dichloromethane = 10:1) to afford 0.412 g (63.8%) of the target compound. ^{1}**H NMR** (400 MHz, Chloroform-*d*) δ (ppm): 8.79 (dd, J = 7.8, 1.1 Hz, 1H), 8.42 (s, 1H), 8.27–8.16 (m, 3H), 8.15–7.99 (m, 4H), 7.79–7.73 (m, 2H), 7.50–7.40 (m, 3H). ^{13}**C NMR** (101 MHz, Chloroform-*d*) δ (ppm): 131.08, 130.76, 130.19, 129.96, 129.30, 129.27, 127.48, 127.47, 126.56, 126.16, 125.16, 125.14, 124.83, 124.66, 124.15, 123.43, 123.19, 122.80, 122.33, 119.28, 93.05, 86.78. **HRMS** (EI), m/z calcd: 302.1096.1096 $[M]^{+\cdot}$, found: 302.10905.

Synthesis of 1-NMe2-Py

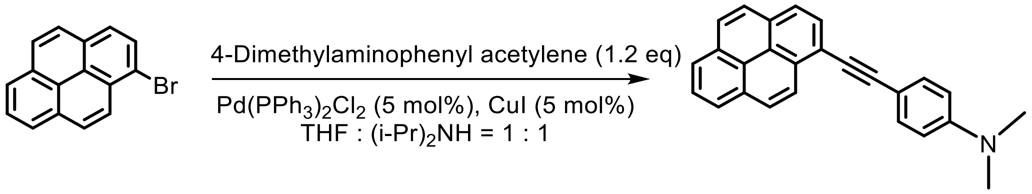

Scheme S8. Synthetic route and chemical structure of **1-N(Me)$_2$-Py**.

1-Bromopyrene (0.60 g, 2.10 mmol), bis(triphenylphosphine)palladium(II) dichloride (0.075 g, 0.10 mmol), and copper(I) iodide (0.020 g, 0.10 mmol) were added to a flask. 4-Dimethylaminophenyl acetylene (0.363 g, 2.50 mmol) was dissolved in tetrahydrofuran (3 mL) and added, followed by tetrahydrofuran (7 mL) and diisopropylamine (10 mL). The mixture was stirred under nitrogen at 80 °C for 44 h and quenched with dichloromethane. The product was purified by column chromatography (petroleum ether/dichloromethane = 2:1) to afford 0.450 g (61.1%) of the target compound. ^{1}**H NMR** (400 MHz, Chloroform-*d*) δ (ppm): 8.70 (d, J = 9.1 Hz, 1H), 8.24–8.16 (m, 4H), 8.12 (d, J = 8.0 Hz, 1H), 8.09–7.99 (m, 3H), 7.68–7.58 (m, 2H), 6.75 (d, J = 8.4 Hz, 2H), 3.04 (s, 6H). ^{13}**C NMR** (101 MHz, Chloroform-*d*) δ (ppm): 150.14, 132.83, 131.46, 131.31, 131.16, 130.61, 129.26, 127.92, 127.65, 127.28, 126.09, 125.82, 125.31, 125.26, 124.55, 124.53, 124.42, 118.91, 111.94, 96.60, 86.69, 40.27. **HRMS** (ESI), m/z calcd: 346.1551 $[M+H]^{+}$, found: 346.15847.

Synthesis of 2-NMe2-Py

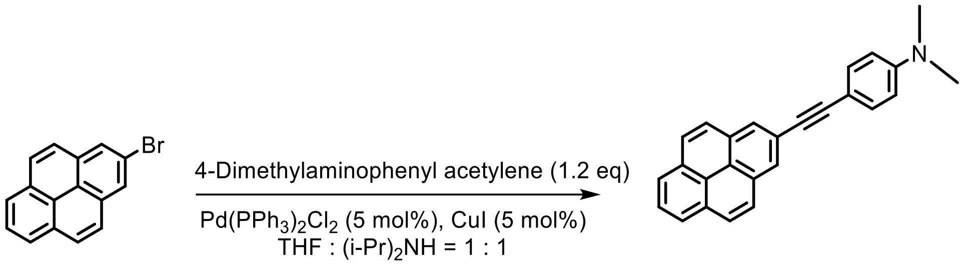

Scheme S9. Synthetic route and chemical structure of **2-N(Me)2-Py**.

2-Bromopyrene (0.60 g, 2.10 mmol), bis(triphenylphosphine)palladium(II) dichloride (0.075 g, 0.10 mmol), and copper(I) iodide (0.020 g, 0.10 mmol) were added to a flask. 4-Dimethylaminophenyl acetylene (0.363 g, 2.50 mmol) was dissolved in tetrahydrofuran (3 mL) and added, followed by tetrahydrofuran (7 mL) and diisopropylamine (10 mL). The mixture was stirred under nitrogen at 80 °C for 44 h and quenched with dichloromethane. The product was purified by column chromatography (petroleum ether/dichloromethane = 2:1) to afford 0.269 g (36.4%) of the target compound. ^{1}**H NMR** (400 MHz, Chloroform-*d*) δ (ppm): 8.31 (s, 2H), 8.16 (d, J = 7.6 Hz, 2H), 8.08–7.96 (m, 5H), 7.57–7.49 (m, 2H), 6.72 (d, J = 8.4 Hz, 2H), 3.02 (s, 6H). ^{13}**C NMR** (101 MHz, Chloroform-*d*) δ (ppm): 131.84, 130.12, 130.04, 126.86, 126.47, 125.97, 125.06, 124.21, 123.44, 122.79, 120.61, 90.13, 87.02, 39.24. **HRMS** (EI), m/z calcd: 345.1517 $[M]^{+\cdot}$, found: 345.15114.

Synthesis of 1-NO2-Py

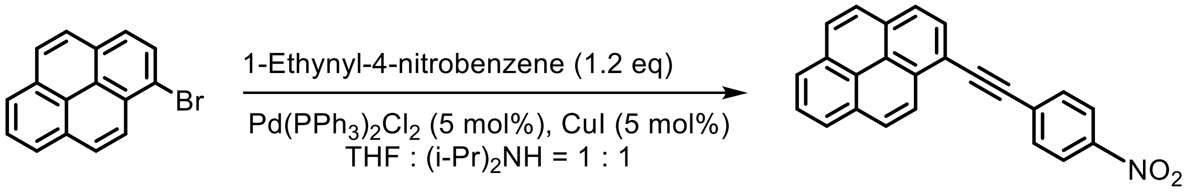

Scheme S10. Synthetic route and chemical structure of **1-NO2-Py**.

1-Bromopyrene (0.60 g, 2.10 mmol), bis(triphenylphosphine)palladium(II) dichloride (0.075 g, 0.10 mmol), and copper(I) iodide (0.020 g, 0.10 mmol) were added to a flask. 1-Ethynyl-4-nitrobenzene (0.368 g, 2.50 mmol) was dissolved in tetrahydrofuran (3 mL) and added, followed by tetrahydrofuran (7 mL) and diisopropylamine (10 mL). The mixture was stirred under nitrogen at 80 °C for 24 h and quenched with dichloromethane. The product was purified by column chromatography (petroleum ether/dichloromethane = 4:1) to afford 0.244 g (33.1%) of the target compound. ^{1}**H NMR** (400 MHz, Chloroform-*d*) δ (ppm): 8.62 (d, J = 9.1 Hz, 1H), 8.25 (ddd, J = 18.0, 8.2, 4.5 Hz, 6H), 8.19–8.12 (m, 2H), 8.10–8.02 (m, 2H), 7.88–7.77 (m, 2H). ^{13}**C NMR** (101 MHz, Chloroform-*d*) δ (ppm): 146.92, 132.23, 132.01, 131.18, 130.93, 130.46, 129.87, 128.85, 128.80, 127.20, 126.45, 126.03, 125.10, 124.59, 124.43, 124.18, 123.76, 116.29, 94.18, 93.18. **HRMS** (ESI), m/z calcd: 348.0980 $[M+H]^{+}$, found: 348.10181.

Synthesis of 2-NO2-Py

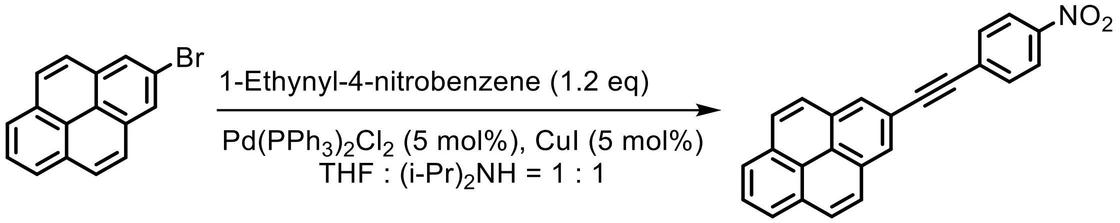

Scheme S11. Synthetic route and chemical structure of **2-NO2-Py**.

2-Bromopyrene (0.60 g, 2.10 mmol), bis(triphenylphosphine)palladium(II) dichloride (0.075 g, 0.10 mmol), and copper(I) iodide (0.020 g, 0.10 mmol) were added to a flask. 1-Ethynyl-4-nitrobenzene (0.368 g, 2.50 mmol) was dissolved in tetrahydrofuran (3 mL) and added, followed by tetrahydrofuran (7 mL) and diisopropylamine (10 mL). The mixture was stirred under nitrogen at 80 °C for 24 h and quenched with dichloromethane. The product was purified by column chromatography (petroleum ether/dichloromethane = 4:1) to afford 0.121 g (16.3%) of the target compound. **^{1}H NMR** (400 MHz, Chloroform-*d*) δ (ppm): 8.36 (s, 2H), 8.28 – 8.25 (m, 2H), 8.21 (d, J = 7.6 Hz, 2H), 8.12 (d, J = 9.0 Hz, 2H), 8.08–8.02 (m, 3H), 7.79–7.73 (m, 2H). **^{13}C NMR** (101 MHz, Chloroform-*d*) δ (ppm): 145.97, 131.33, 130.30, 130.14, 129.28, 127.36, 126.78, 125.83, 125.63, 124.57, 123.67, 123.25, 122.70, 118.30, 94.45, 86.88. **HRMS** (ESI), m/z calcd: 348.0980 $[M+H]^+$, found: 348.10178

Synthesis of 2-H-Phen

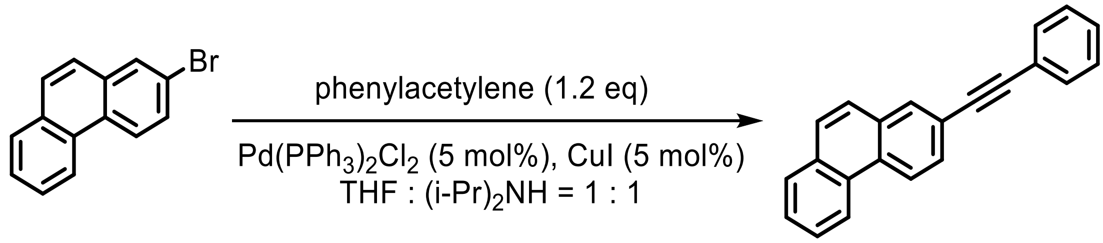

Scheme S12. Synthetic route and chemical structure of **2-H-Phen**.

2-Bromophenanthrene (0.54 g, 2.10 mmol), bis(triphenylphosphine)palladium(II) dichloride (0.075 g, 0.10 mmol), and copper(I) iodide (0.020 g, 0.10 mmol) were added to a flask. Phenylacetylene (0.25 g, 2.50 mmol) was dissolved in tetrahydrofuran (3 mL) and added, followed by tetrahydrofuran (7 mL) and diisopropylamine (10 mL). The mixture was stirred under nitrogen at 80 °C for 18 h. The product was purified by column chromatography (petroleum ether/dichloromethane = 10:1) to afford 0.205 g (35.2%) of the target compound. **^{1}H NMR** (400 MHz, Chloroform-*d*) δ (ppm): 8.70–8.62 (m, 2H), 8.09 (d, J = 1.8 Hz, 1H), 7.89 (dd, J = 7.8, 1.5 Hz, 1H), 7.82–7.75 (m, 2H), 7.73–7.58 (m, 5H), 7.43–7.35 (m, 3H). **^{13}C NMR** (101 MHz, Chloroform-*d*) δ (ppm): 131.25, 130.79, 130.76, 130.64, 128.92, 128.86, 128.26, 127.62, 127.37, 127.32, 126.63, 125.94, 125.81, 125.42, 122.22, 121.80, 120.26, 89.09, 88.54. **HRMS** (EI), m/z calcd: 278.1096 $[M]^{+\cdot}$, found: 278.10916.

Synthesis of 3-H-Phen

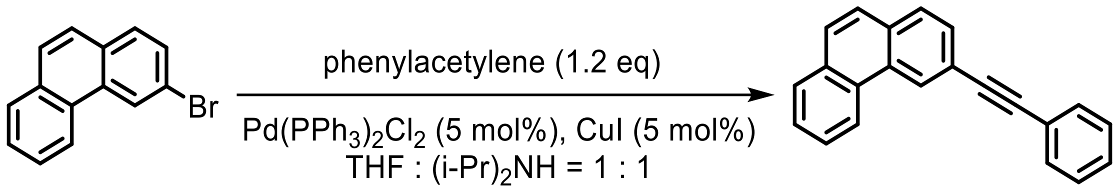

Scheme S13. Synthetic route and chemical structure of **3-H-Phen**.

3-Bromophenanthrene (0.54 g, 2.10 mmol), bis(triphenylphosphine)palladium(II) dichloride (0.075 g, 0.10 mmol), and copper(I) iodide (0.020 g, 0.10 mmol) were added to a flask. Phenylacetylene (0.25 g, 2.50 mmol) was dissolved in tetrahydrofuran (3 mL) and added, followed by tetrahydrofuran (7 mL) and diisopropylamine (10 mL). The mixture was stirred under nitrogen at 80 °C for 18 h. The mixture was stirred under nitrogen at 80 °C for 18 h. The product was purified by column chromatography (petroleum ether/dichloromethane = 10:1) to afford 0.329 g (56.5%) of the target compound. **^{1}H NMR** (400 MHz, Chloroform-*d*) δ (ppm): 8.92 (d, J = 1.4 Hz, 1H), 8.72 (d, J = 8.1 Hz, 1H), 7.93–7.86 (m, 2H), 7.80–7.63 (m, 7H), 7.4 –7.37 (m, 3H). **^{13}C NMR** (101 MHz, Chloroform-*d*) δ (ppm): 131.13, 130.63, 130.56, 129.01, 128.68, 128.19, 127.55, 127.53, 127.37, 127.30, 126.74, 125.87, 125.79, 125.42, 125.27, 122.23, 121.67, 120.13, 89.11, 88.89. **HRMS** (EI), m/z calcd: 278.1096 $[M]^{+\cdot}$, found: 278.10923.

Preparation of polymer films

For photoluminescence spectra and lifetime measurements, 5 mg of corresponding synthesized compound was mixed with poly(methyl methacrylate) (PMMA) (1 g) in dichloromethane. The solution was evenly cast on a quartz plate and allowed to evaporate. The polymer film on the quartz plate (0.5 wt%) is scraped off and transferred to a glass tube, which was then evacuated and sealed. For TA measurements, the loading of the specific compound in the film was increased to 1 wt%.

Preparation of single crystal samples

Single crystals of **1-H-Py** and **2-H-Py** for SCXRD characterization were obtained by slow evaporation of their acetonitrile solutions.

Part II. Structural characterization

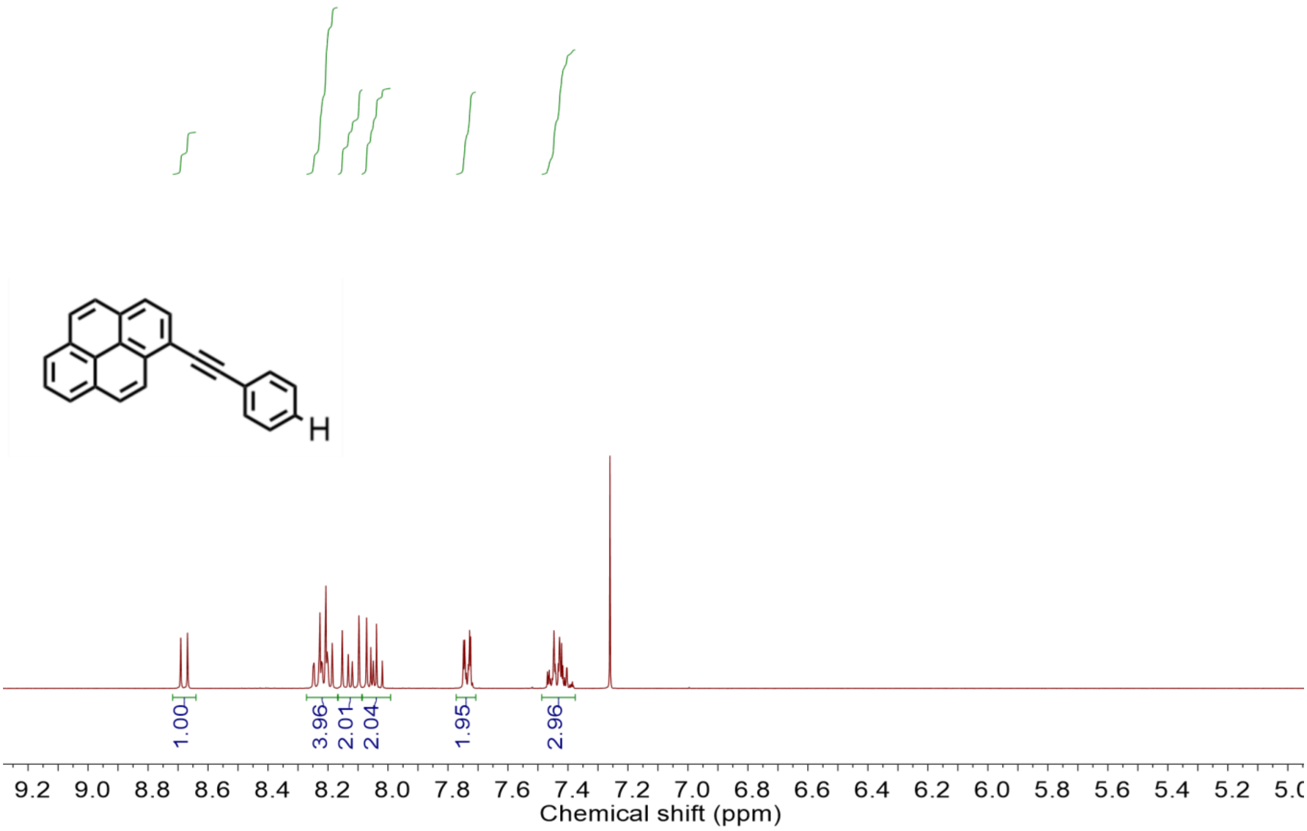

Figure S1. ^{1}H NMR spectrum of **1-H-Py** (400 MHz, Chloroform-d, 298 K).

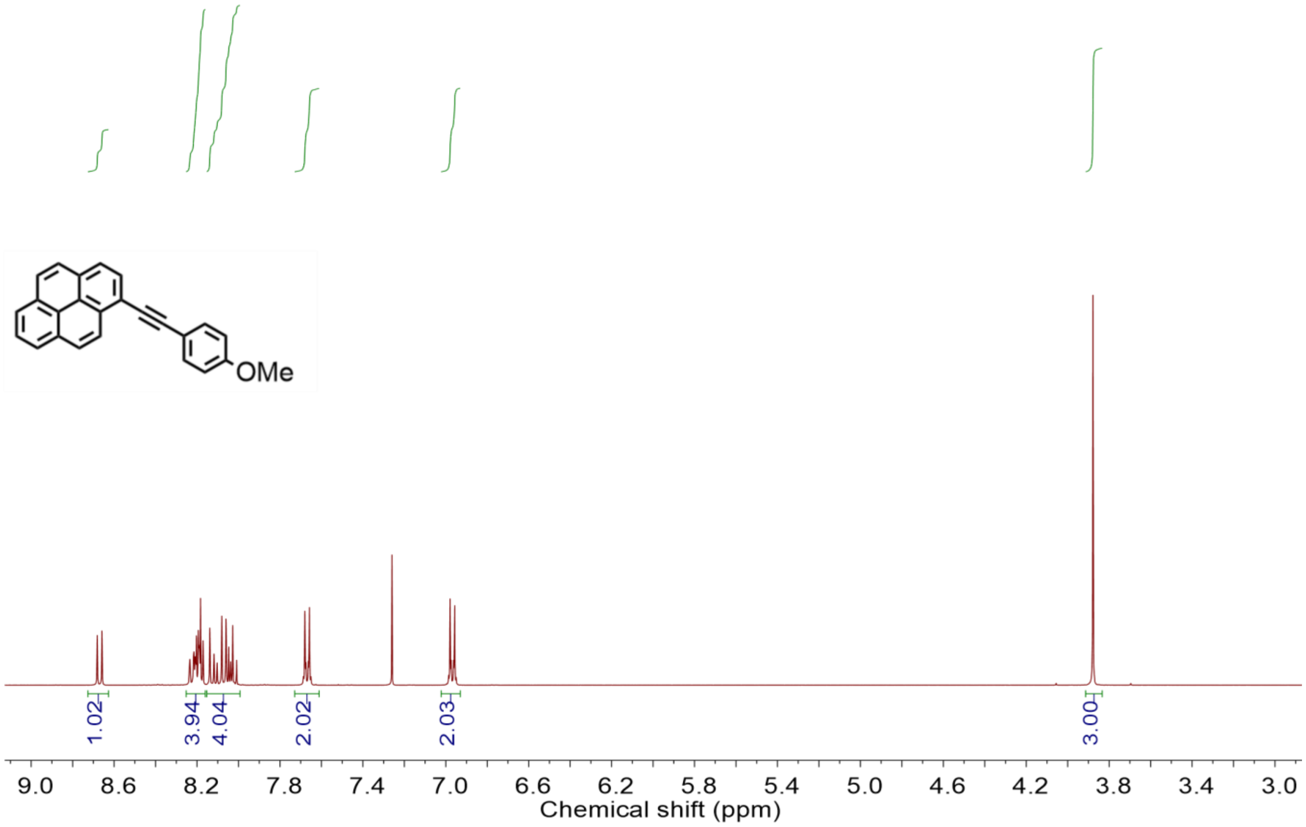

Figure S2. ^{1}H NMR spectrum of **1-OME-Py** (400 MHz, Chloroform-d, 298 K).

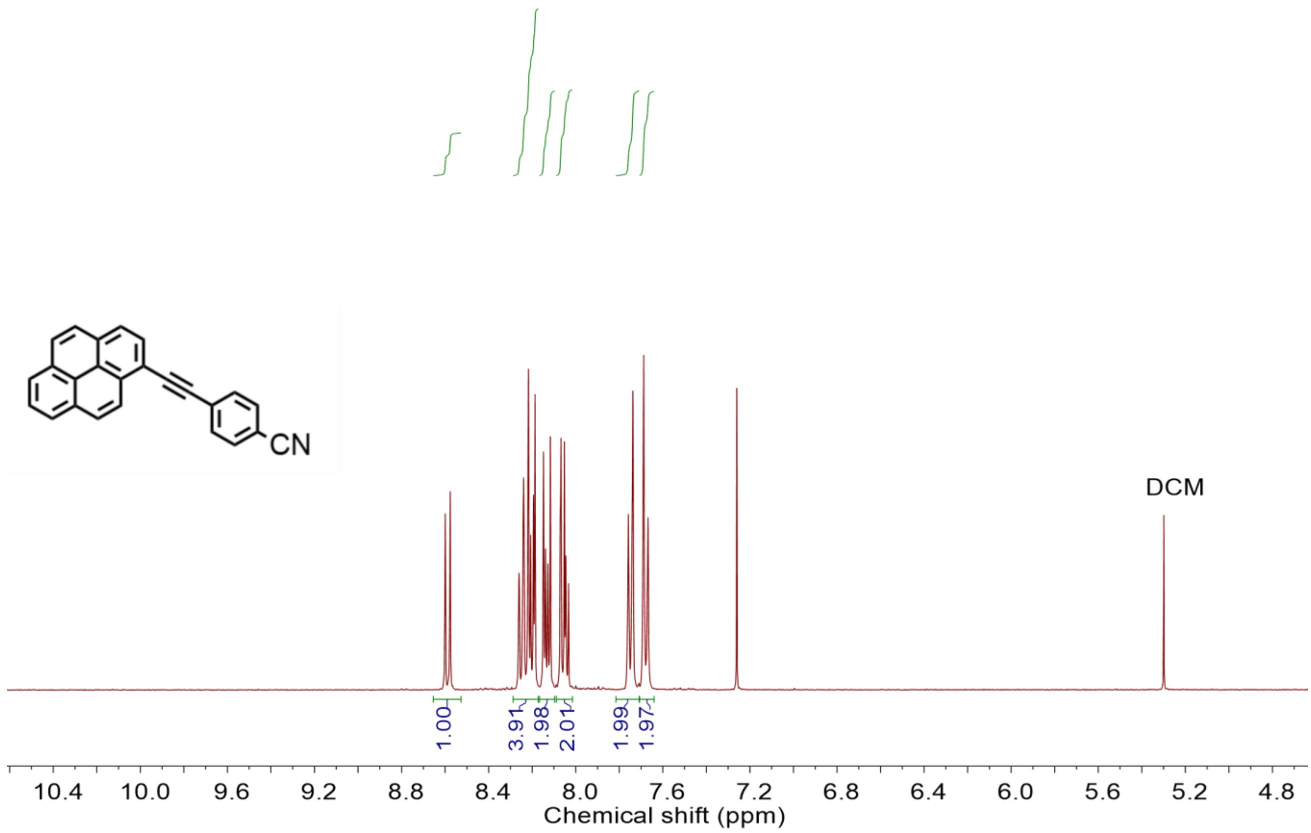

Figure S3. ^{1}H NMR spectrum of **1-CN-Py** (400 MHz, Chloroform-d, 298 K).

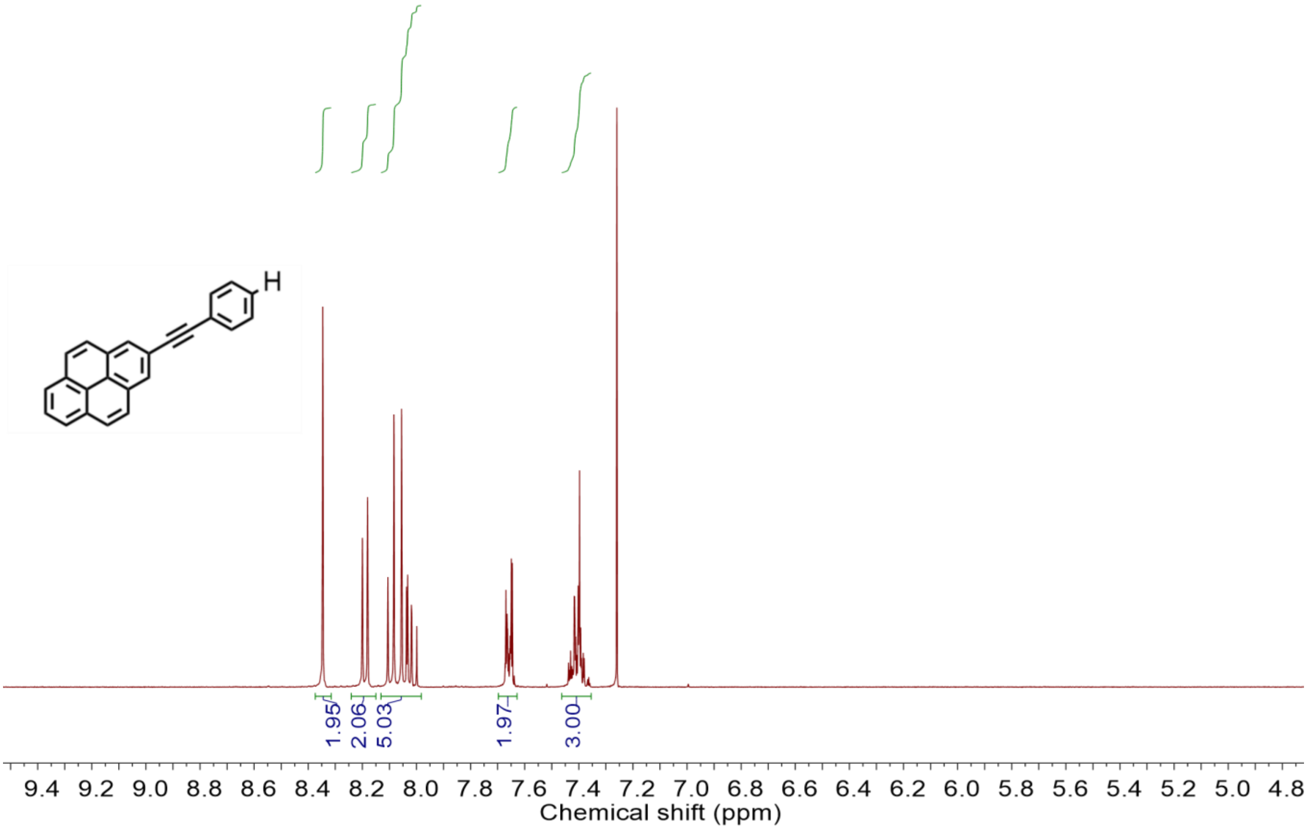

Figure S4. ^1H NMR spectrum of **2-H-Py** (400 MHz, Chloroform-d, 298 K).

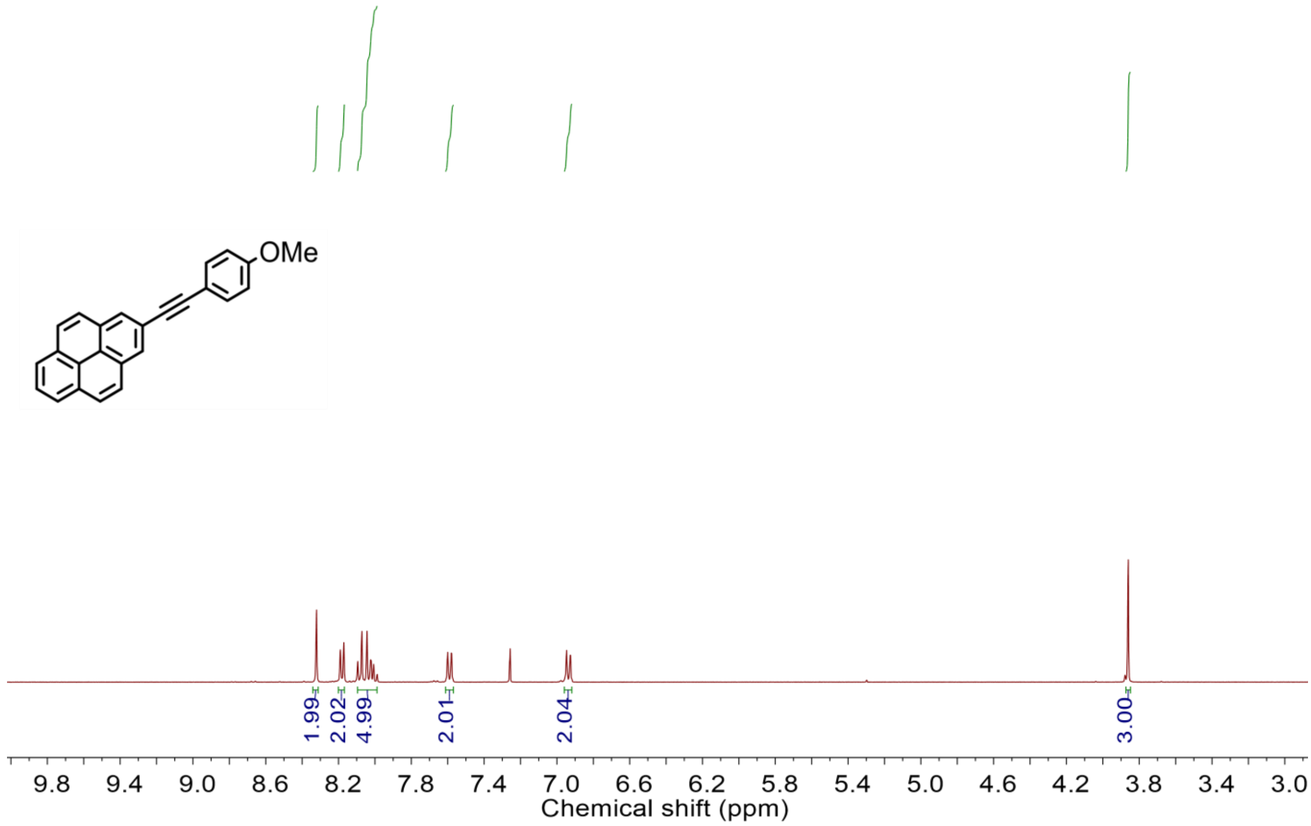

Figure S5. ^{1}H NMR spectrum of **2-OME-Py** (400 MHz, Chloroform-d, 298 K).

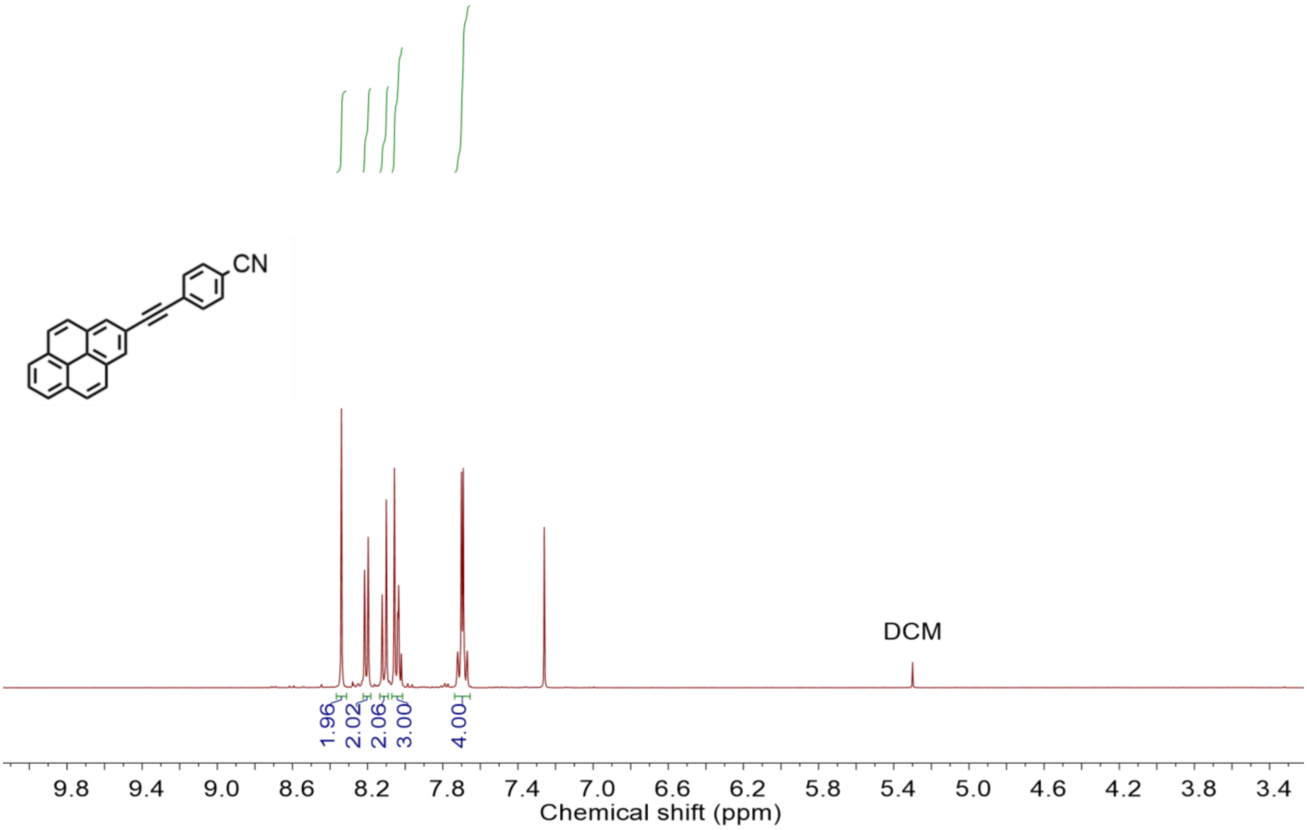

Figure S6. ^{1}H NMR spectrum of **2-CN-Py** (400 MHz, Chloroform-d, 298 K).

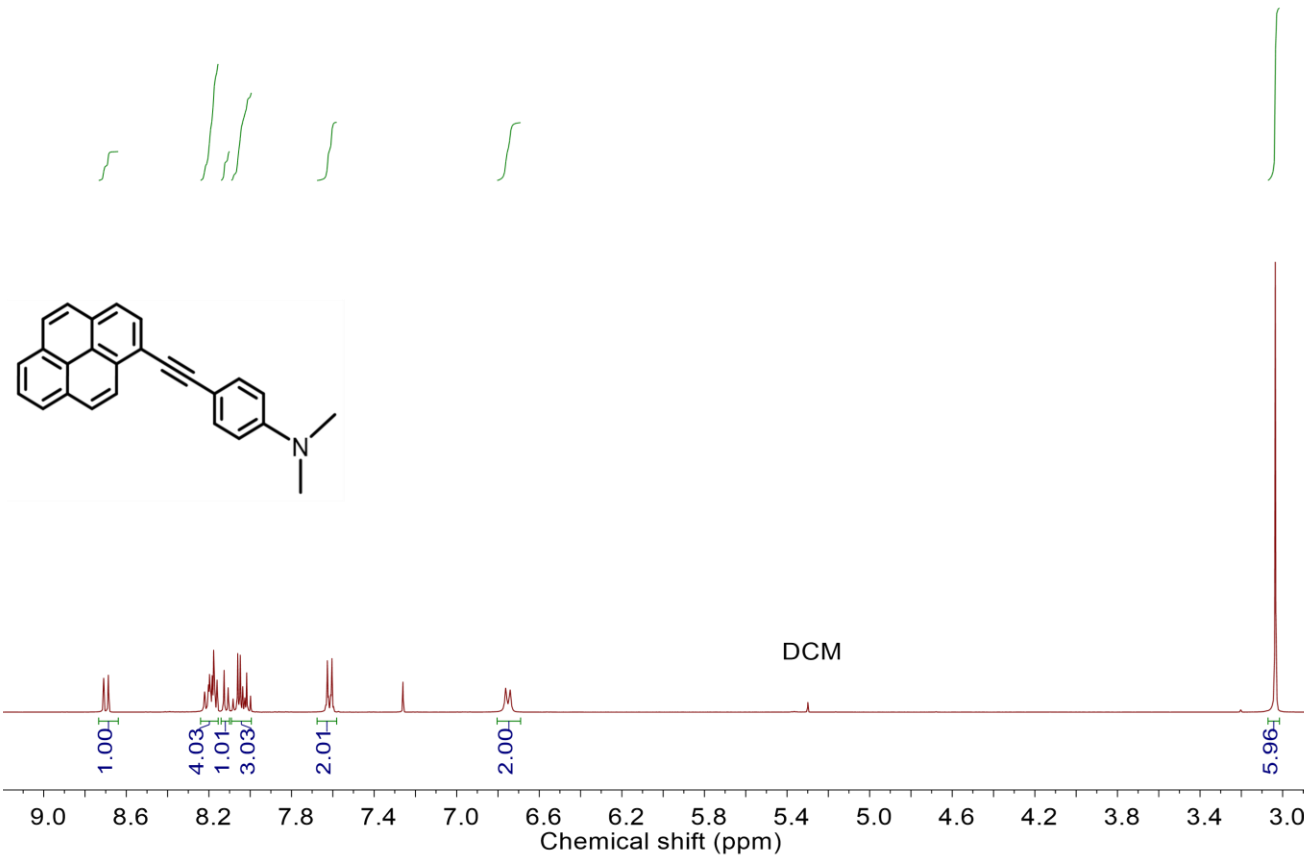

Figure S7. ^{1}H NMR spectrum of **1-N(Me)$_2$-Py** (400 MHz, Chloroform-d, 298 K).

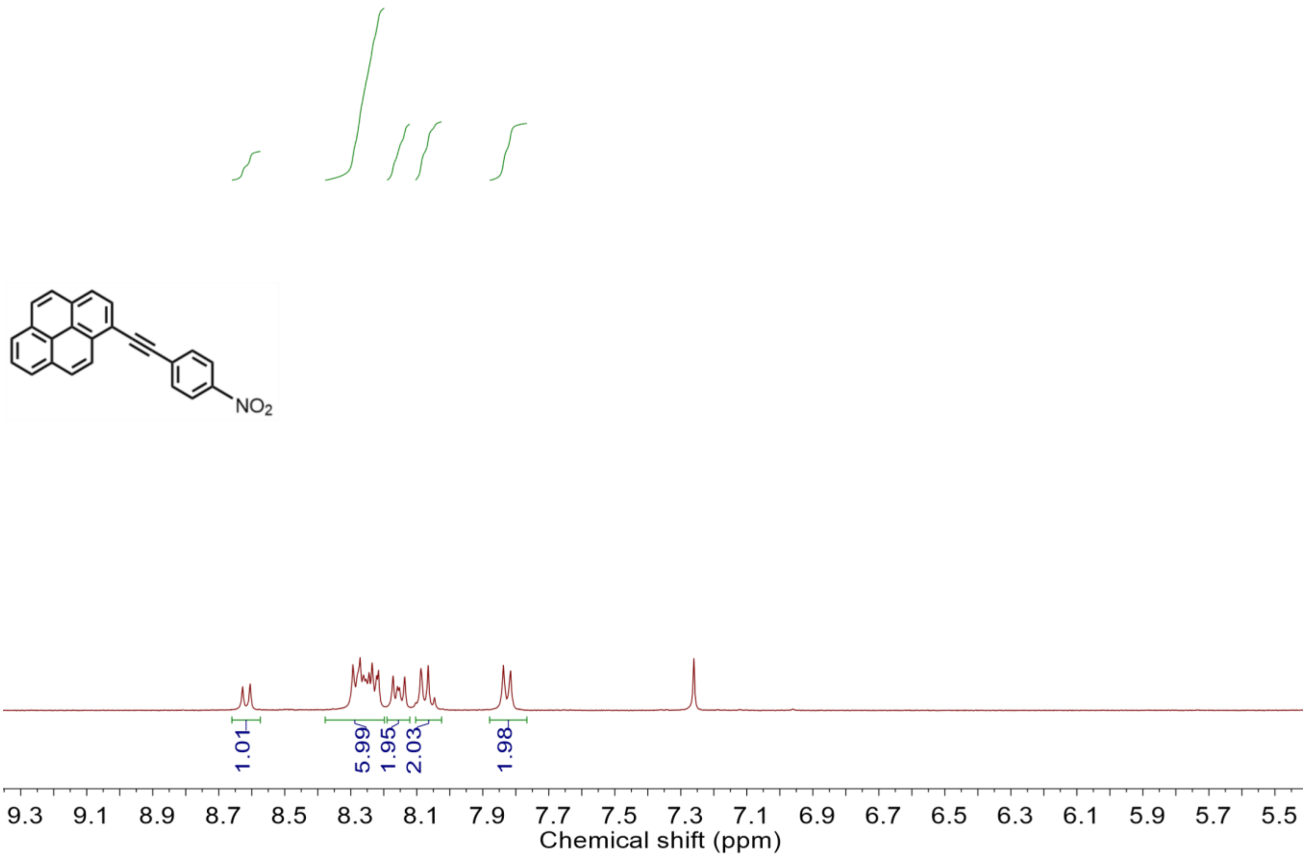

Figure S8. ^{1}H NMR spectrum of **1-NO$_2$-Py** (400 MHz, Chloroform-d, 298 K).

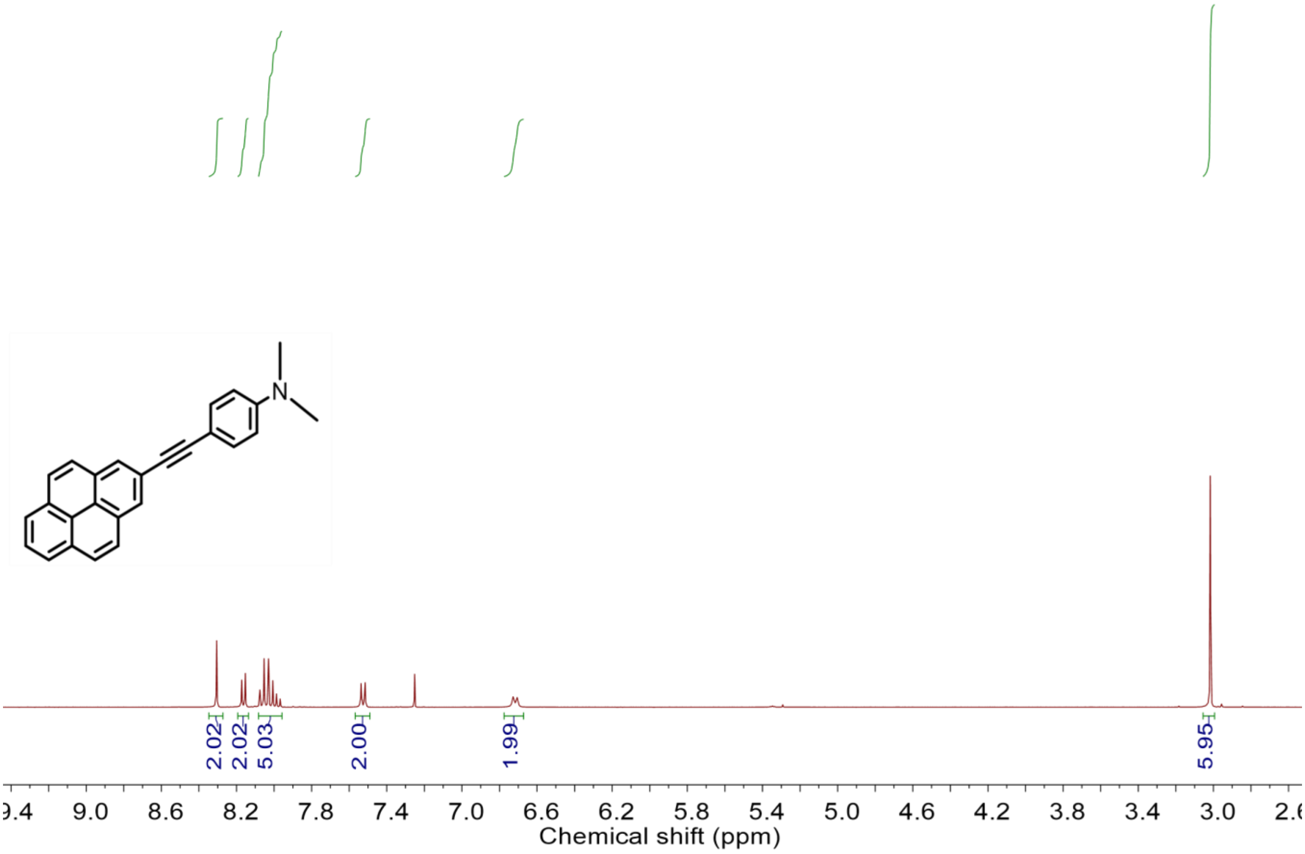

Figure S9. ^{1}H NMR spectrum of **2-N(Me)$_2$-Py** (400 MHz, Chloroform-d, 298 K).

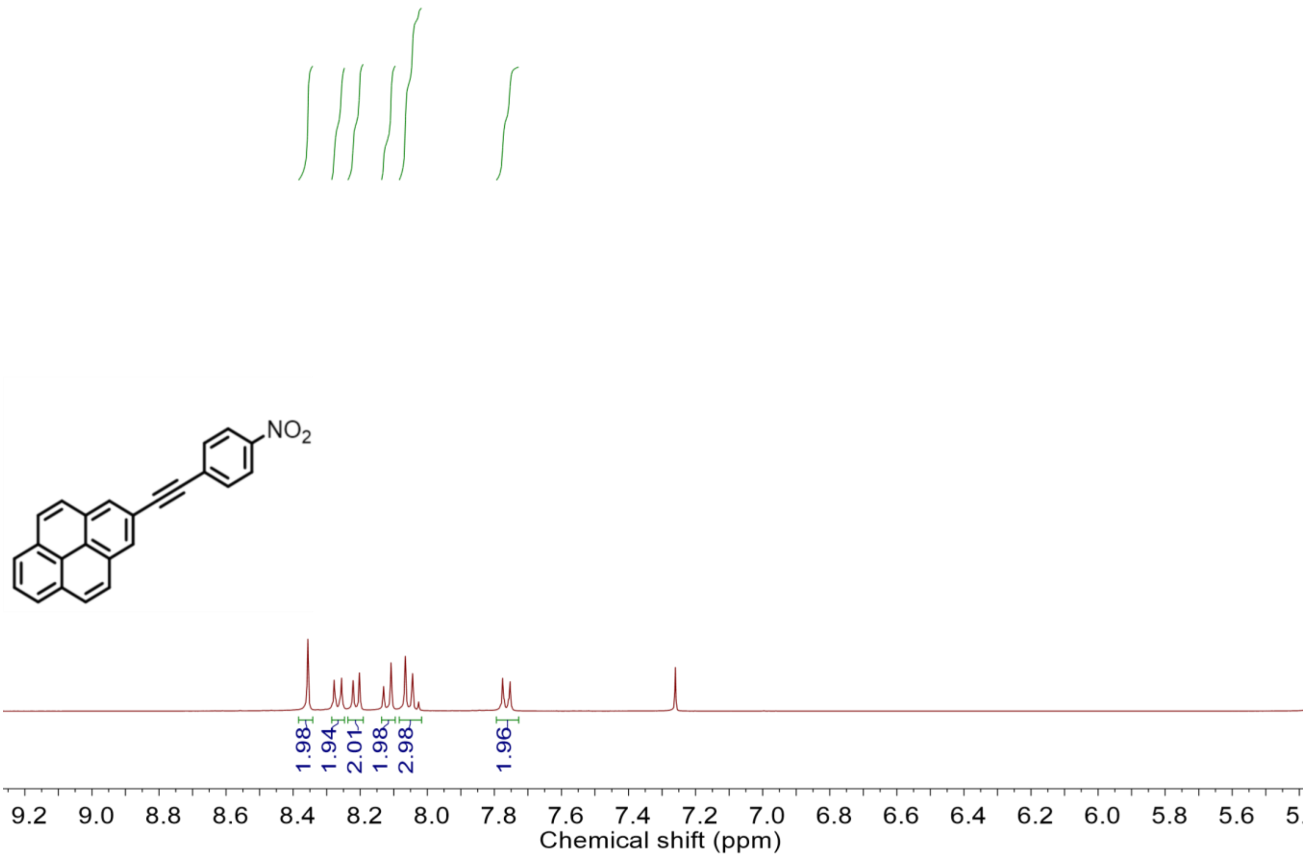

Figure S10. ^{1}H NMR spectrum of **2-NO$_2$-Py** (400 MHz, Chloroform-d, 298 K).

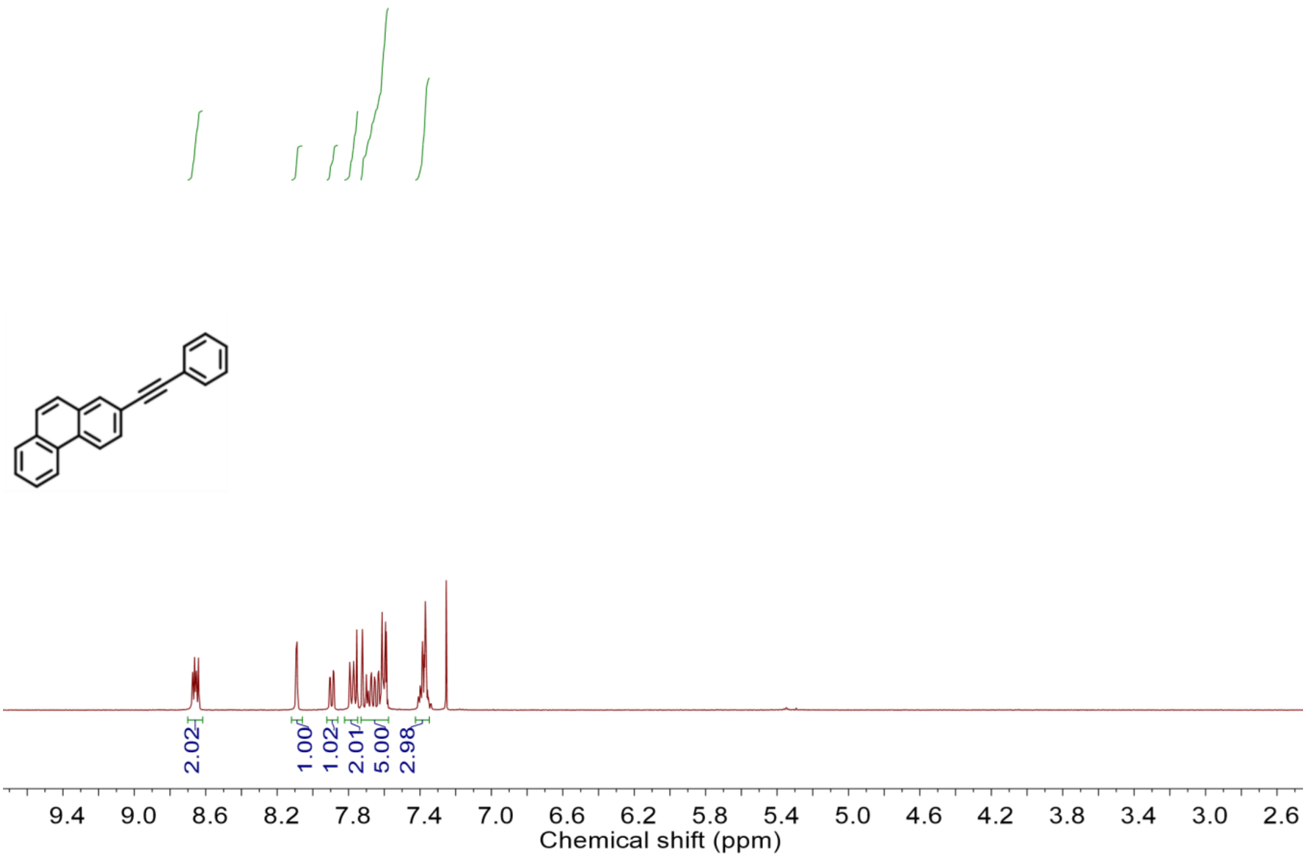

Figure S11. ^{1}H NMR spectrum of **2-H-Phen** (400 MHz, Chloroform-d, 298 K).

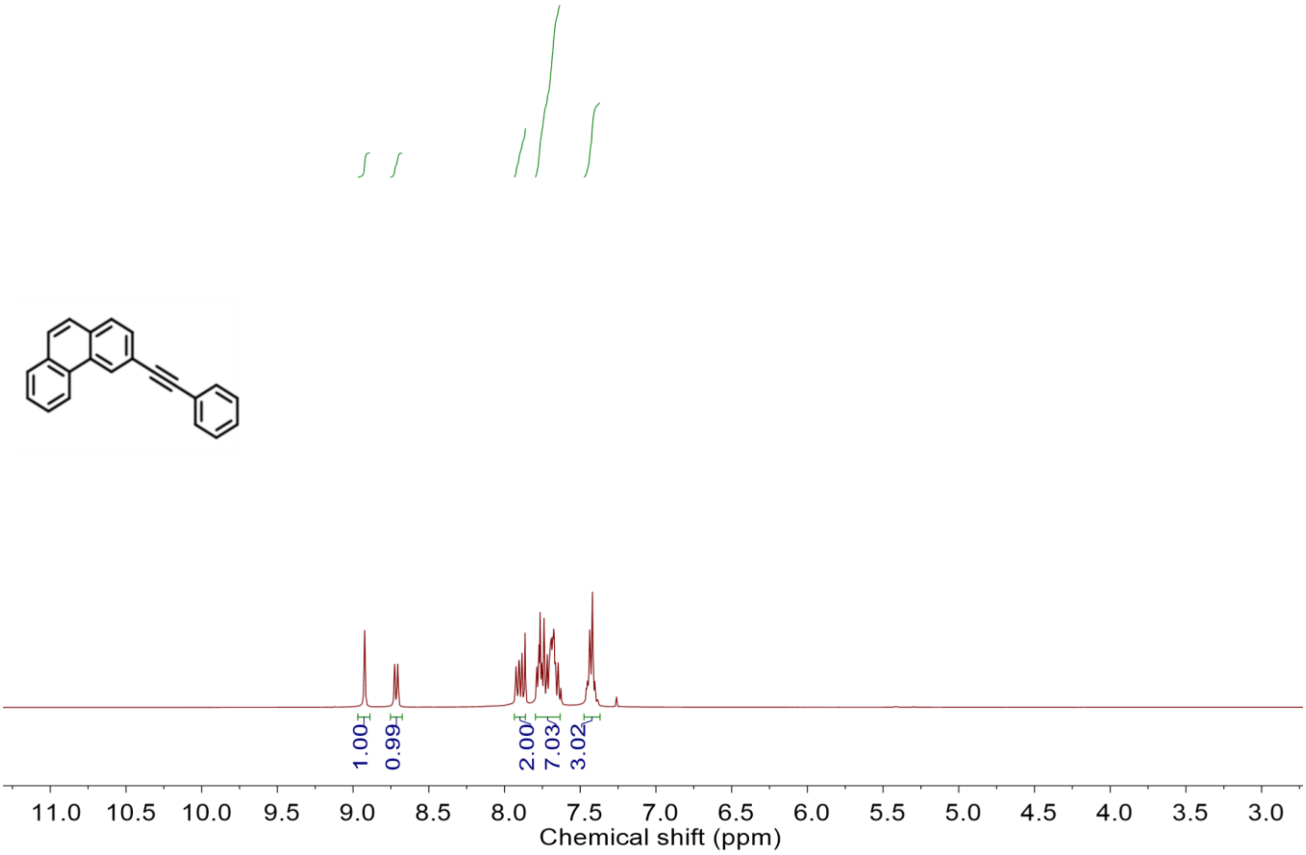

Figure S12. ^{1}H NMR spectrum of **3-H-Phen** (400 MHz, Chloroform-d, 298 K).

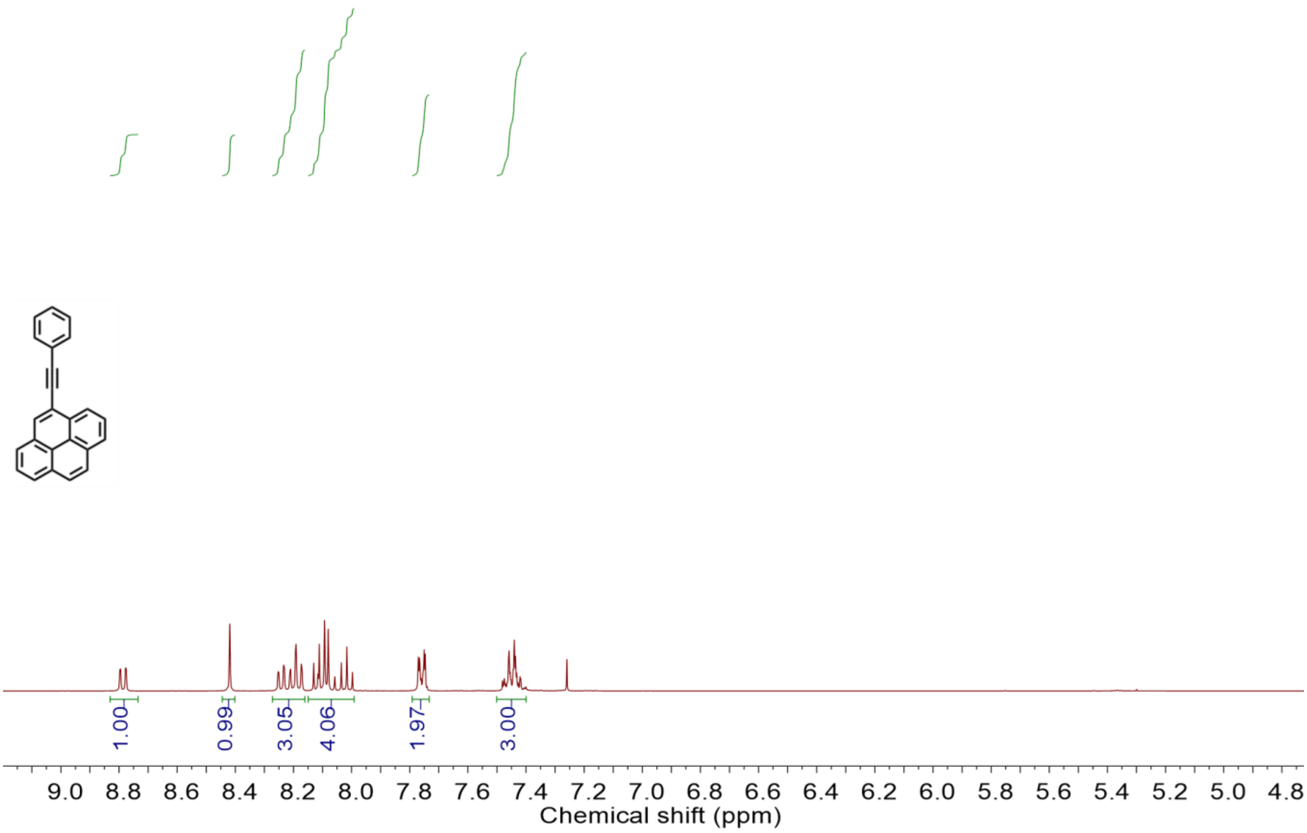

Figure S13. ^{1}H NMR spectrum of **4-H-Py** (400 MHz, Chloroform-d, 298 K).

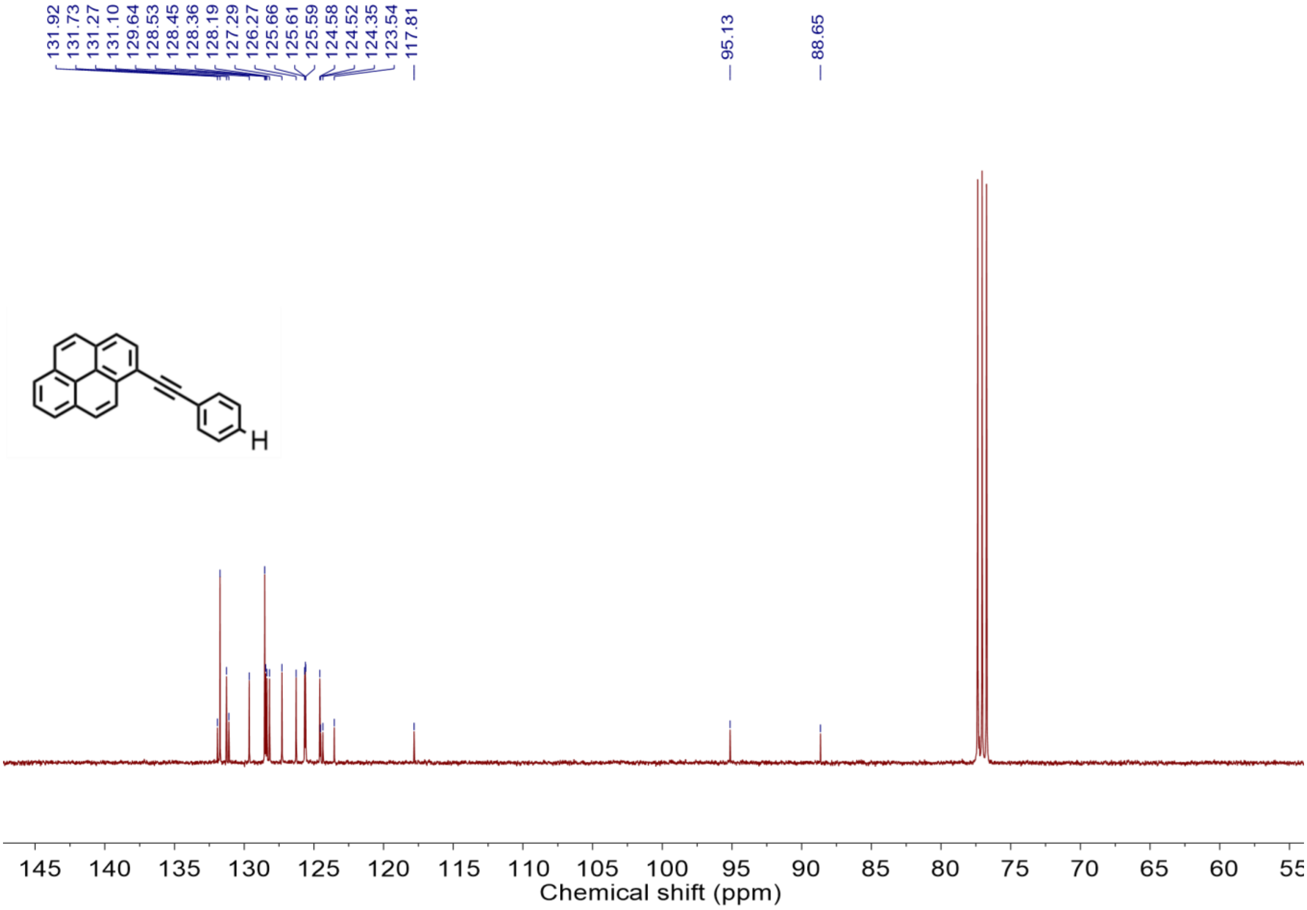

Figure S14. ^{13}C NMR spectrum of **1-H-Py** (101 MHz, Chloroform-d, 298 K).

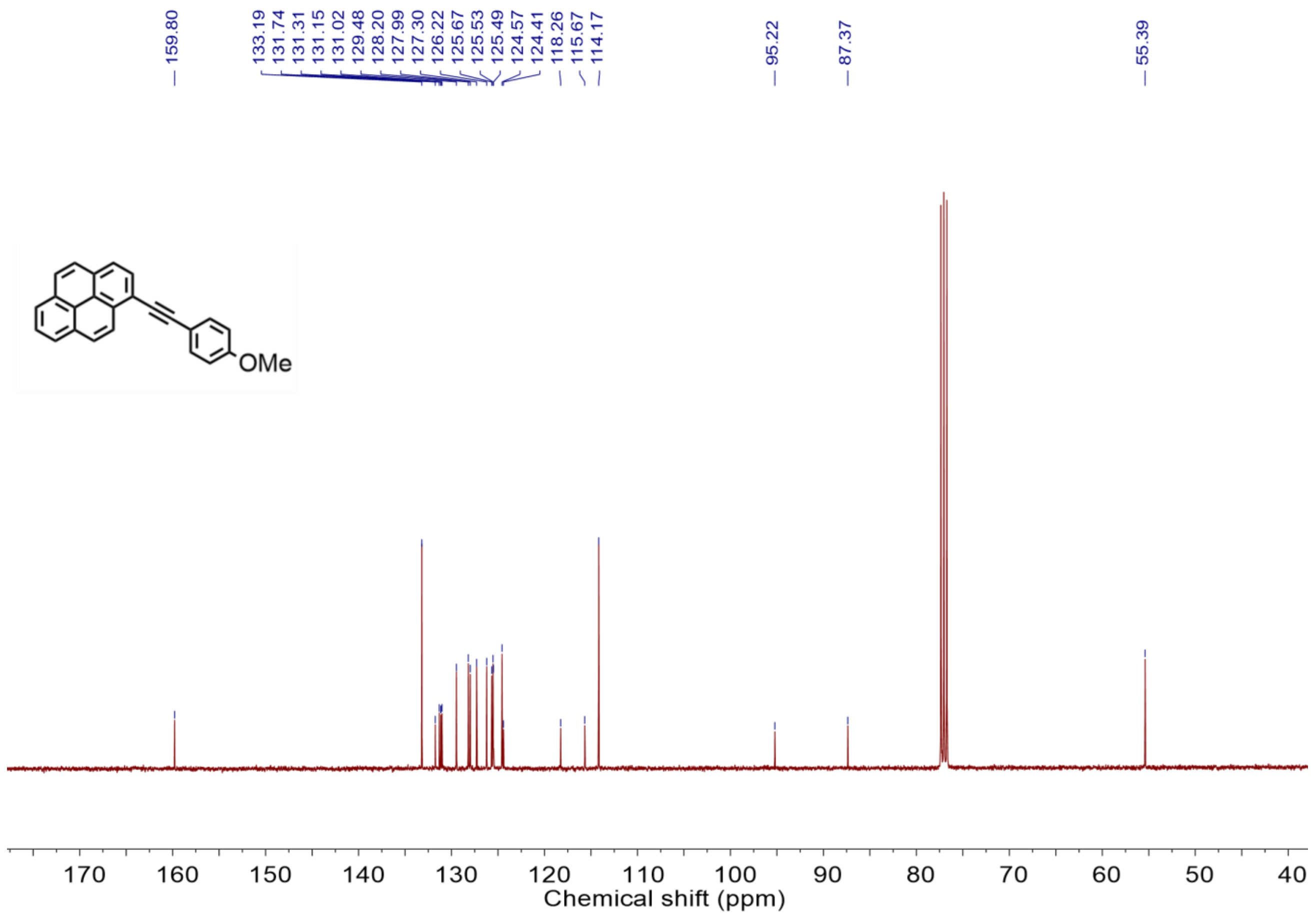

Figure S15 ^{13}C NMR spectrum of **1-OME-Py** (101 MHz, Chloroform-d, 298 K).

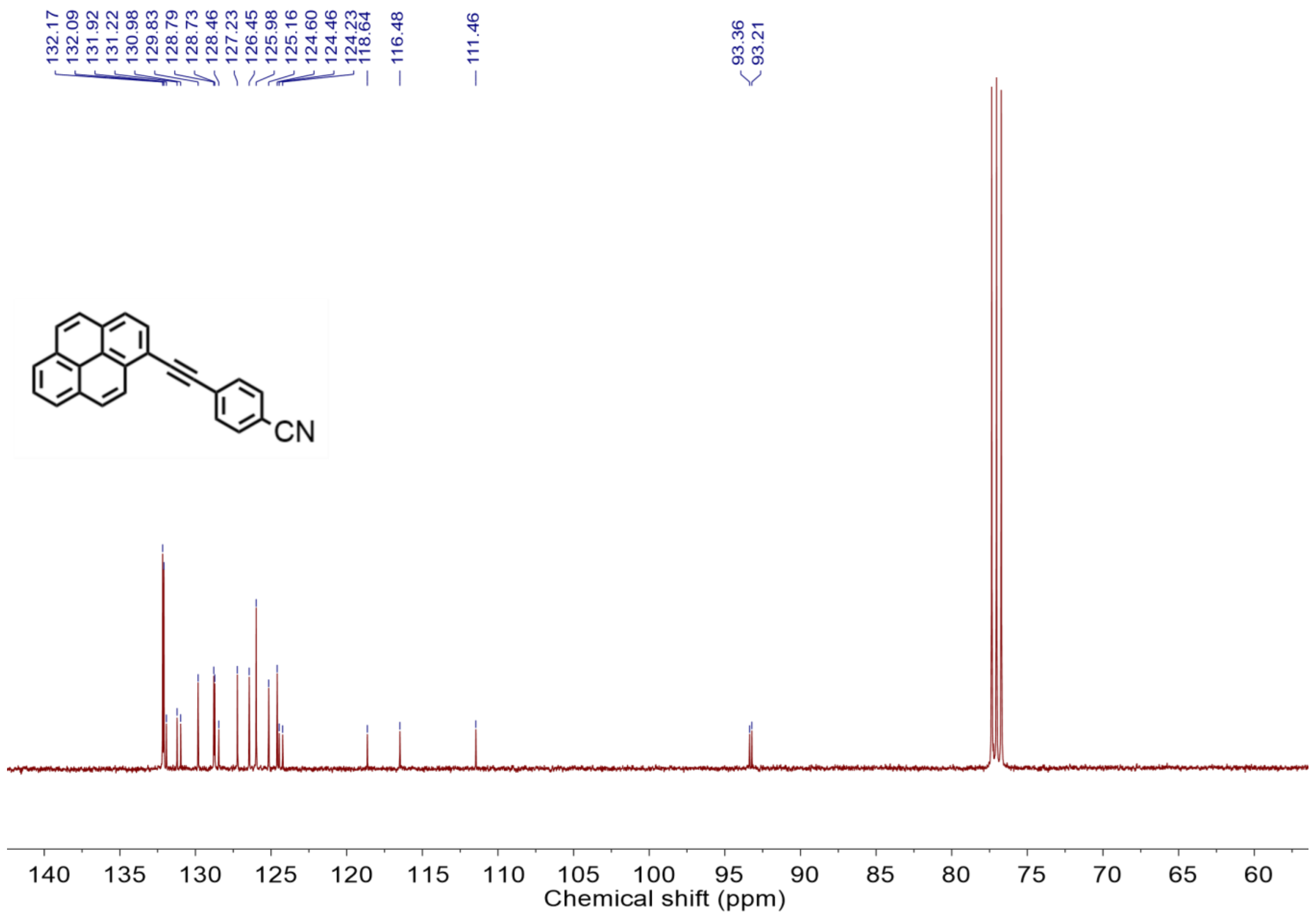

Figure S16. ^{13}C NMR spectrum of **1-CN-Py** (101 MHz, Chloroform-d, 298 K).

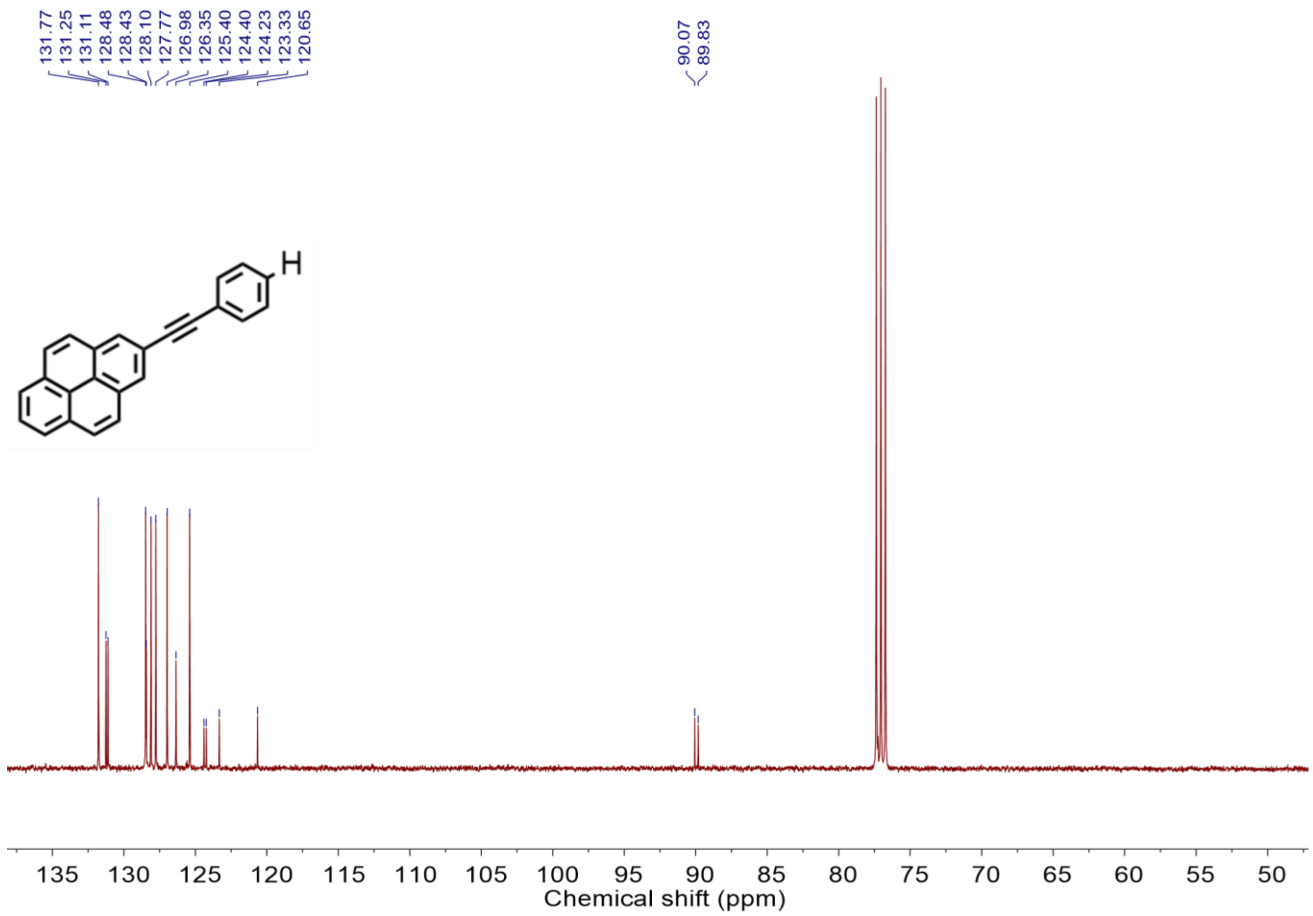

Figure S17. ^{13}C NMR spectrum of **2-H-Py** (101 MHz, Chloroform-d, 298 K).

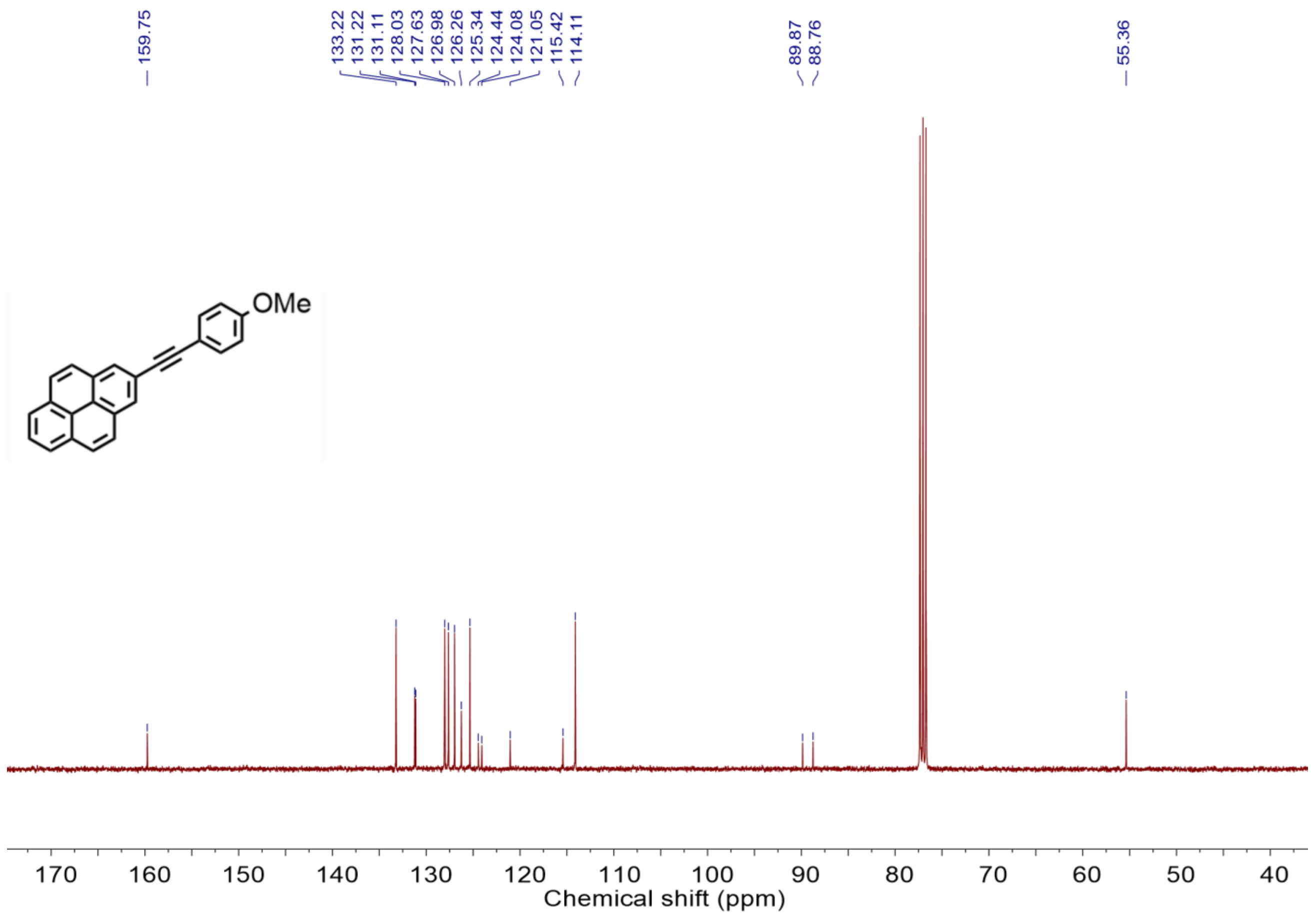

Figure S18. ^{13}C NMR spectrum of **2-OME-Py** (101 MHz, Chloroform-d, 298 K).

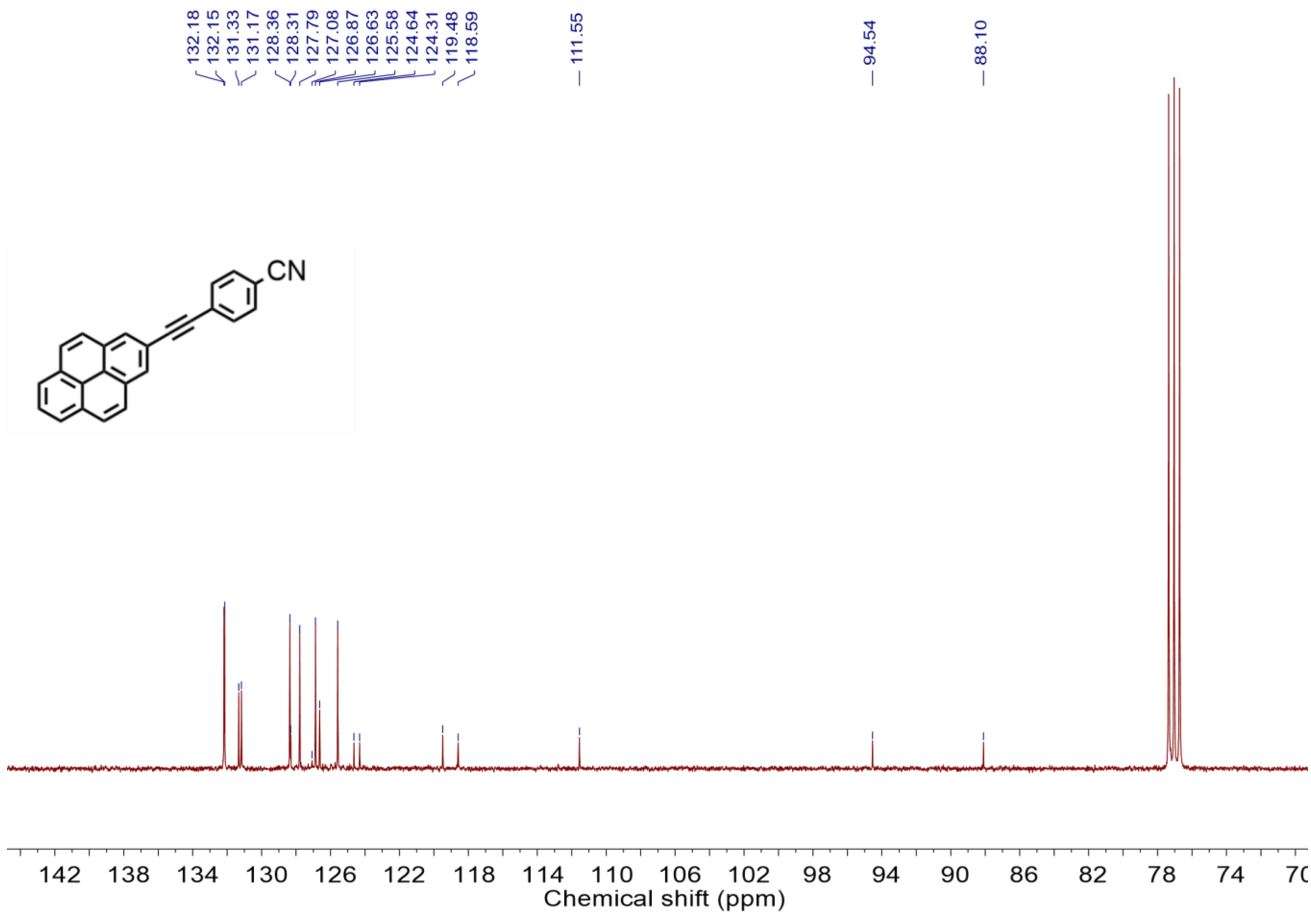

Figure S19. ^{13}C NMR spectrum of **2-CN-Py** (101 MHz, Chloroform-d, 298 K).

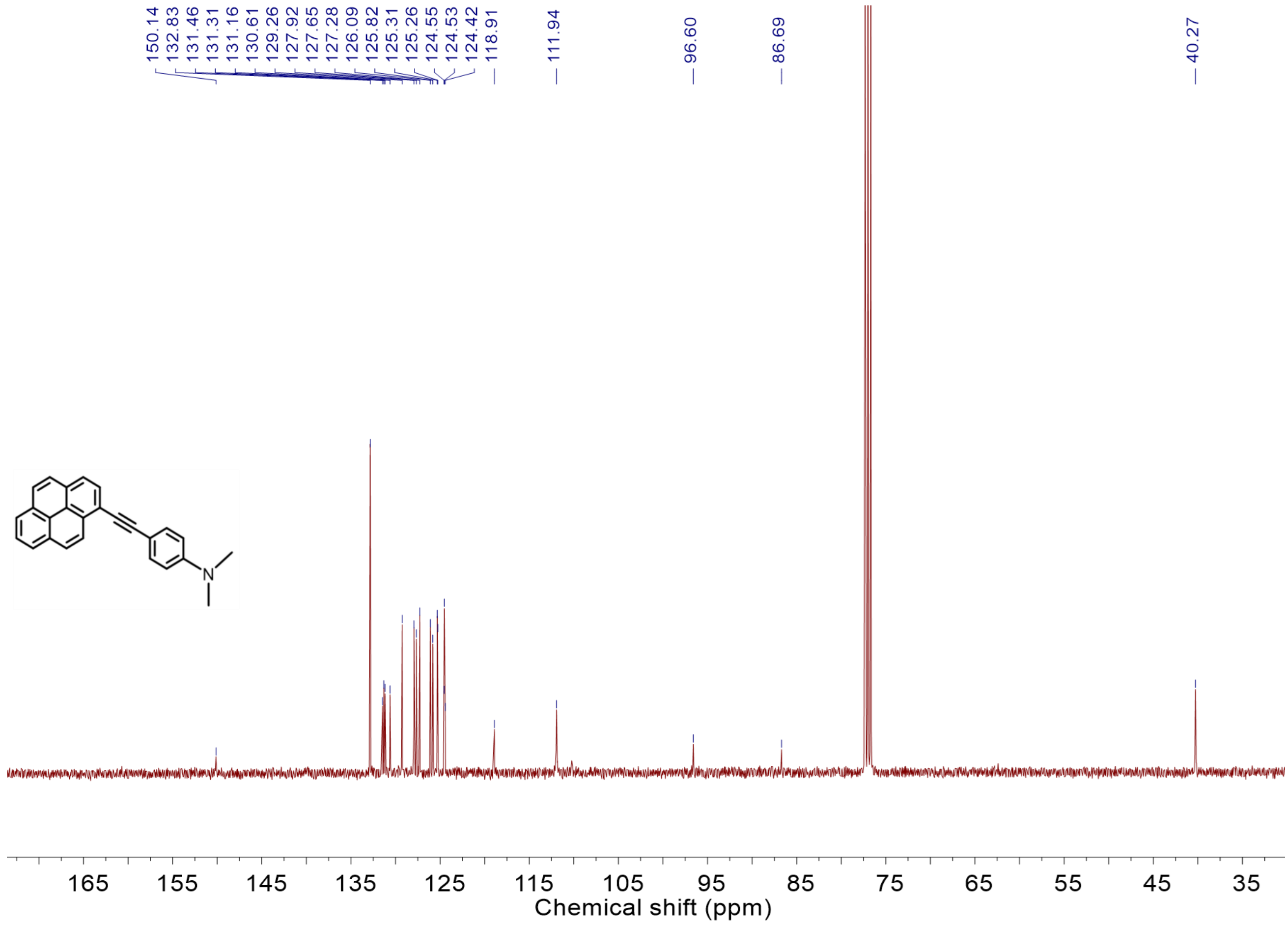

Figure S20. ^{13}C NMR spectrum of **1-N(Me)₂-Py** (101 MHz, Chloroform-d, 298 K).

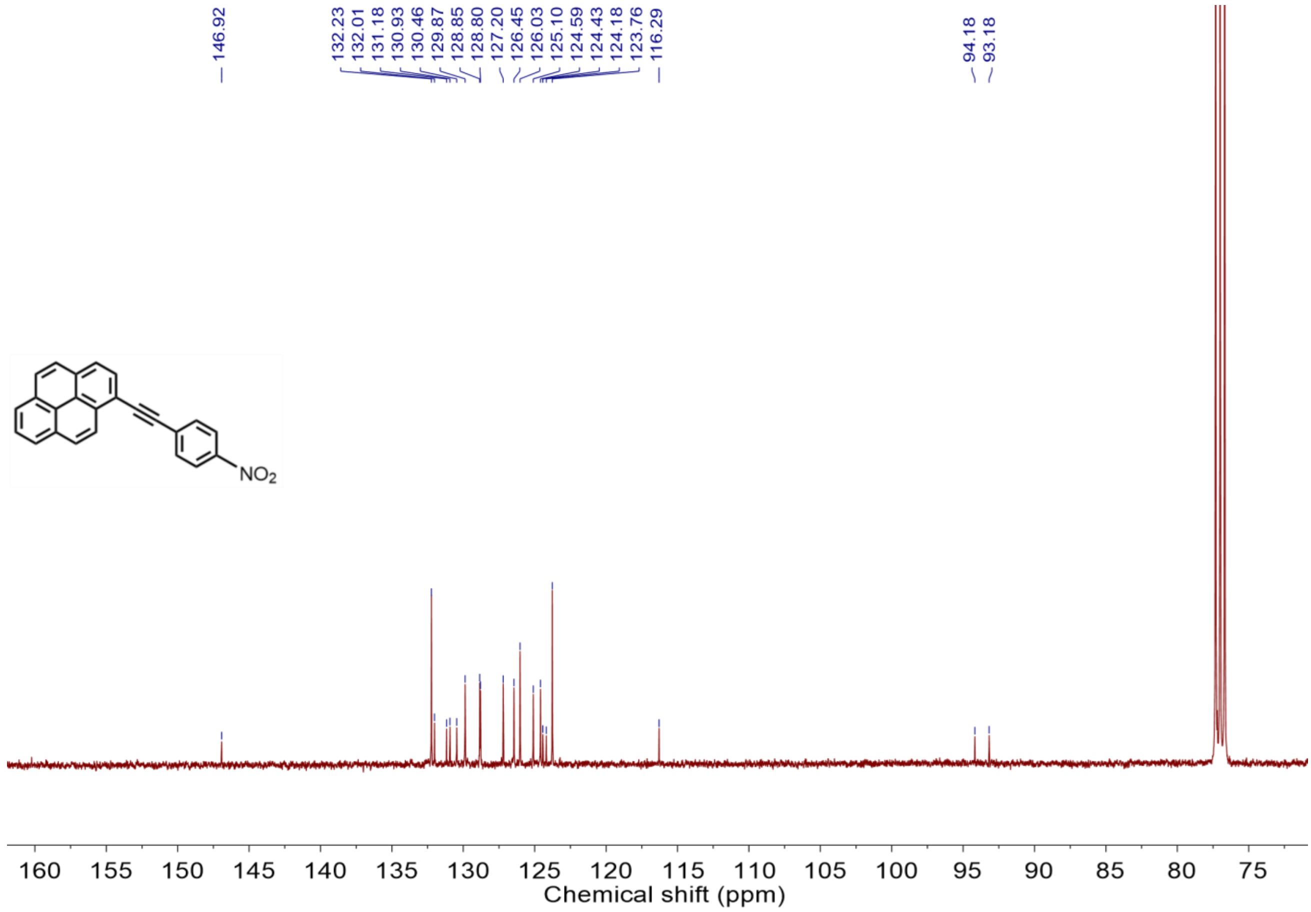

Figure S21. ^{13}C NMR spectrum of **1-NO$_2$-Py** (101 MHz, Chloroform-d, 298 K).

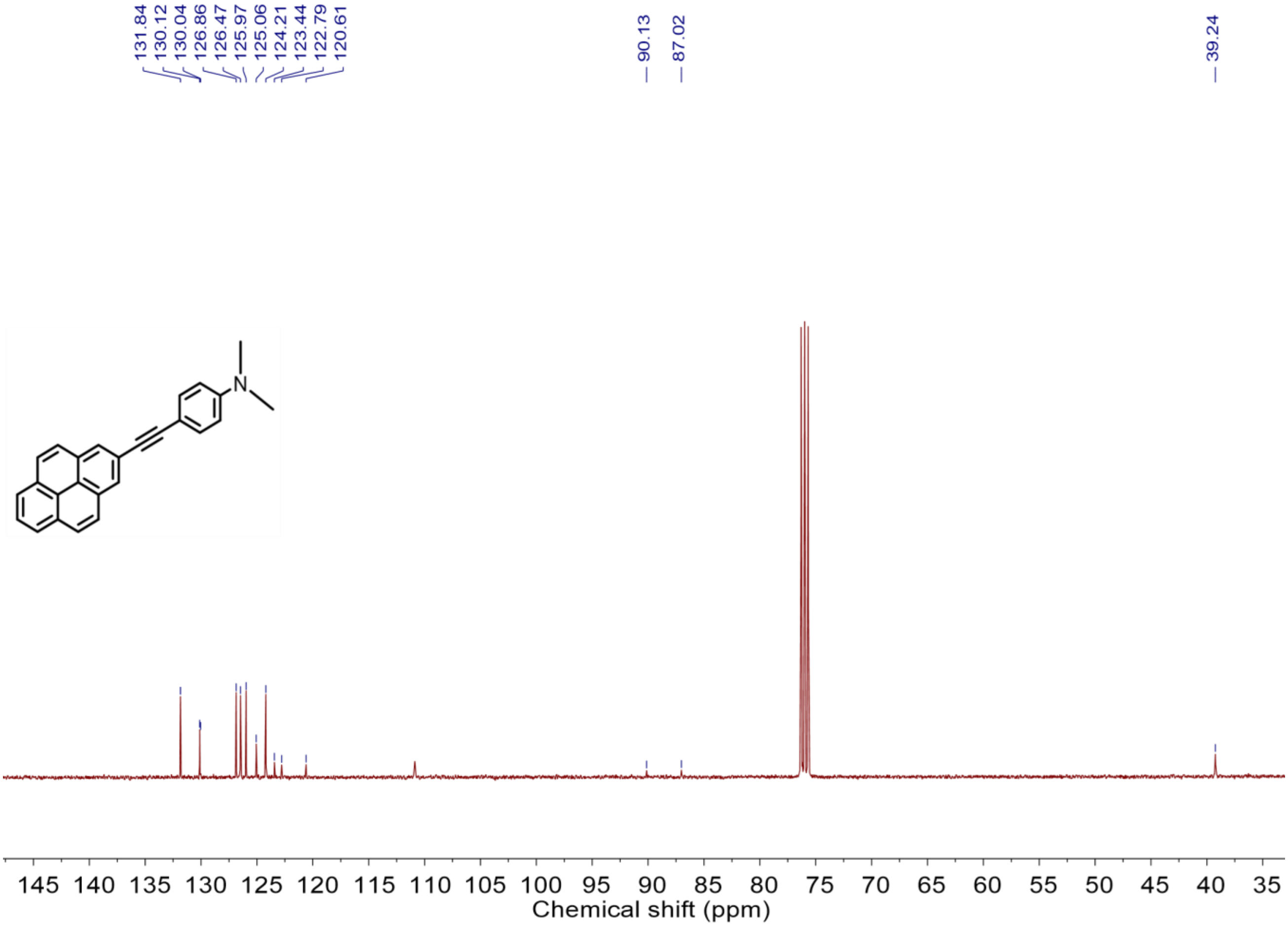

Figure S22. C NMR spectrum of **2-N(Me)$_2$-Py** (101 MHz, Chloroform-d, 298 K)**.**

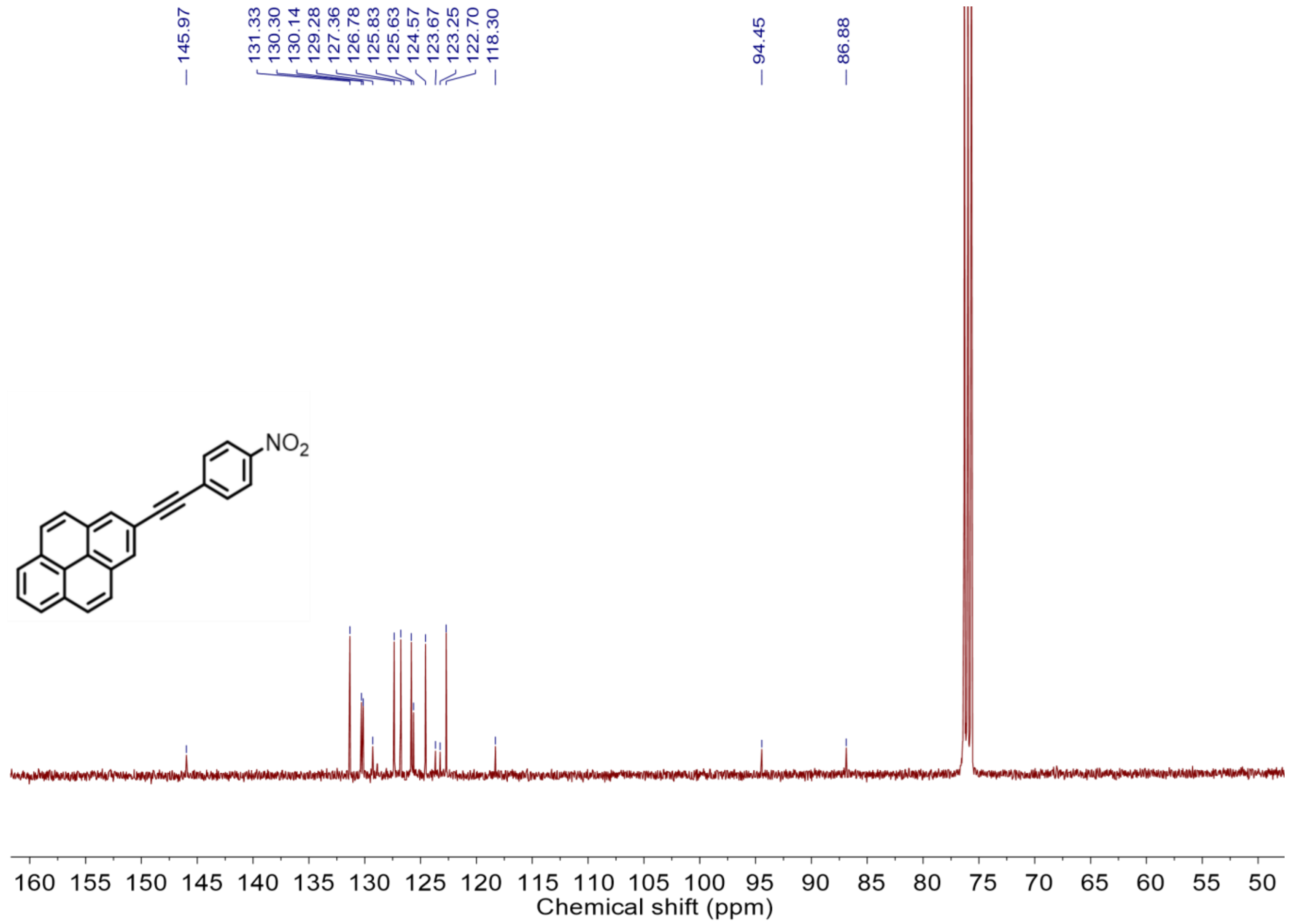

Figure S23. ^{13}C NMR spectrum of **2-NO$_2$-Py** (101 MHz, Chloroform-d, 298 K).

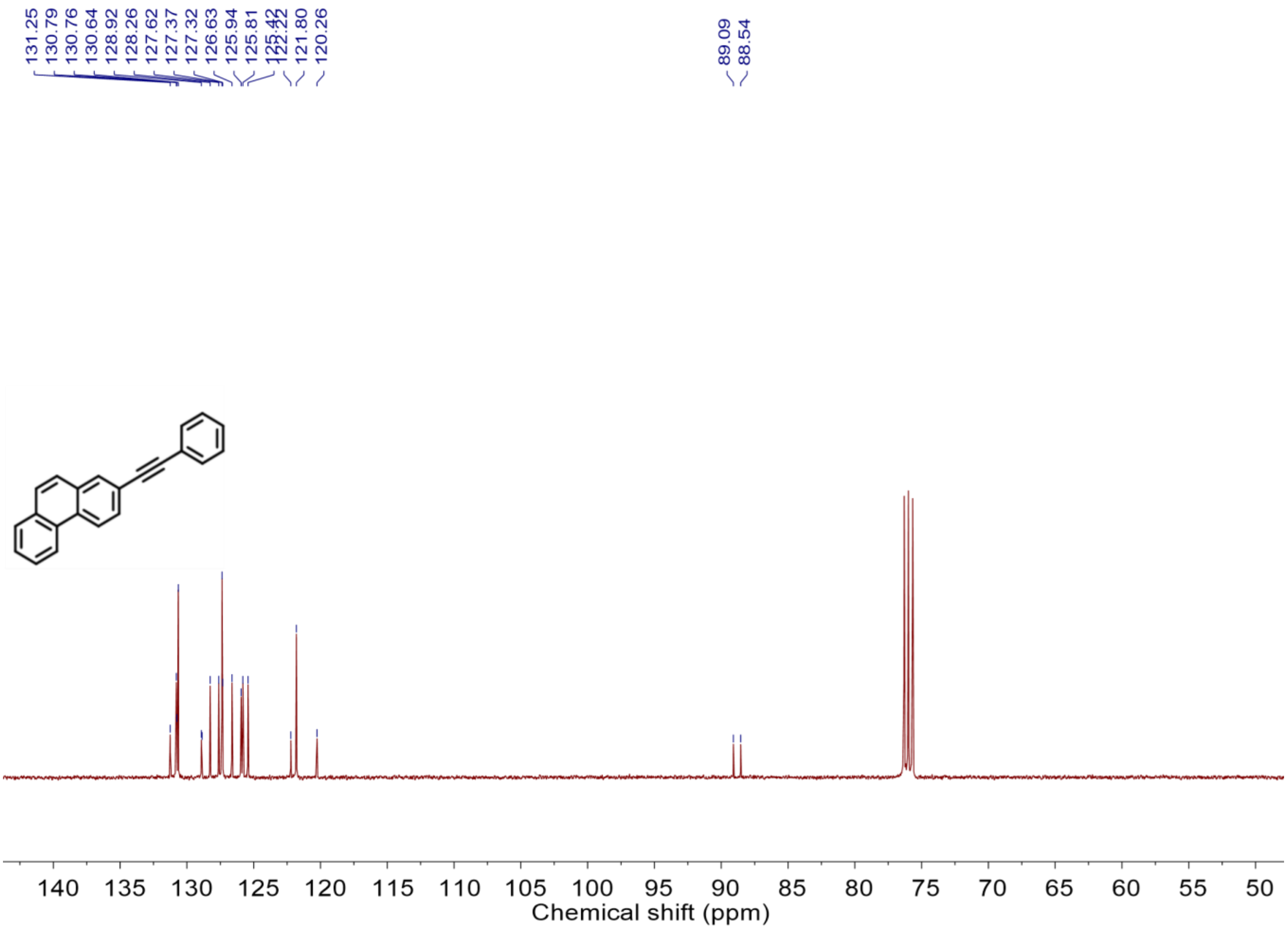

Figure S24. ^{13}C NMR spectrum of **2-H-Phen** (101 MHz, Chloroform-d, 298 K).

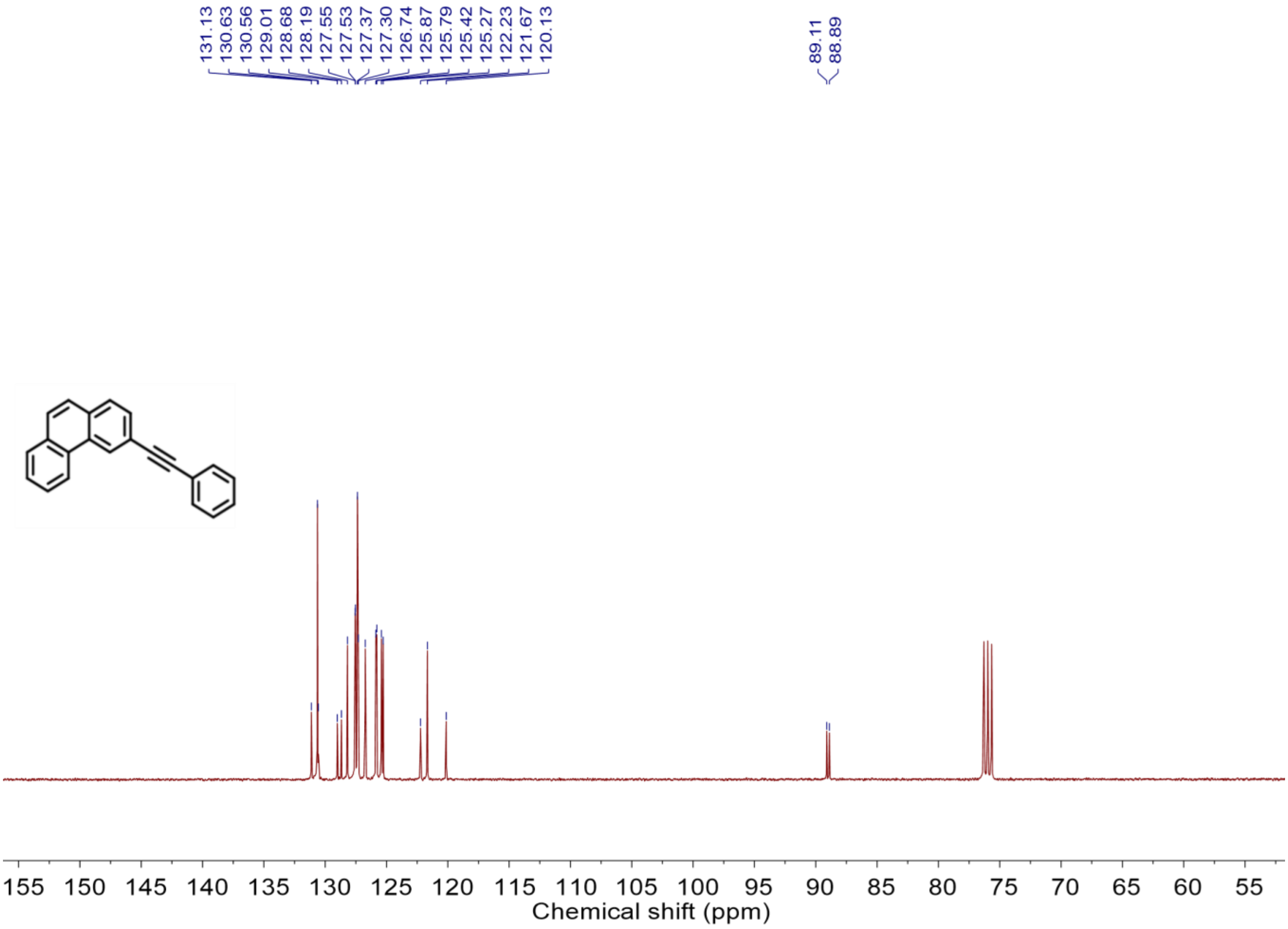

Figure S25. ^{13}C NMR spectrum of **3-H-Phen** (101 MHz, Chloroform-d, 298 K).

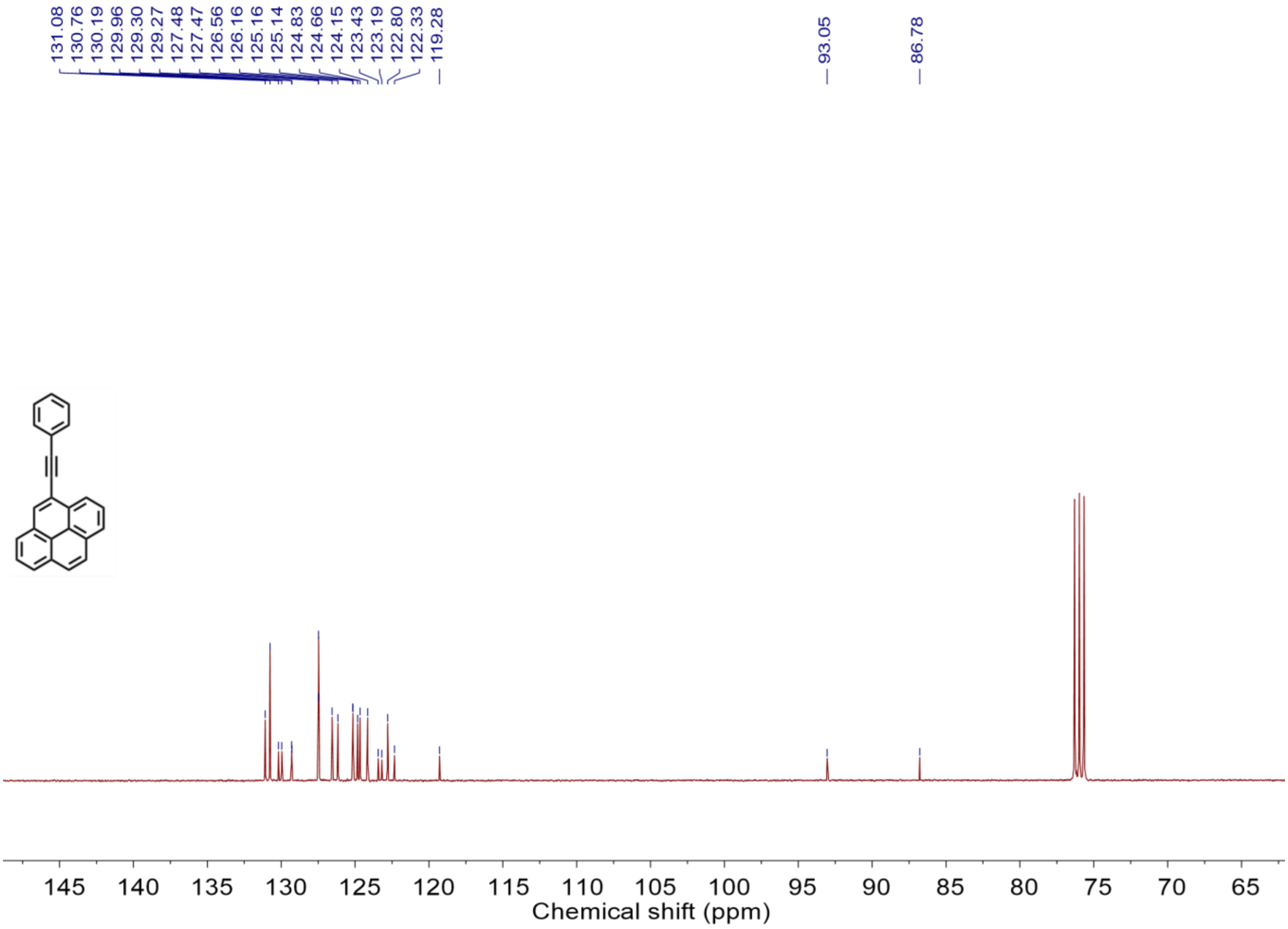

Figure S26. ^{13}C NMR spectrum of **4-H-Py** (101 MHz, Chloroform-d, 298 K).

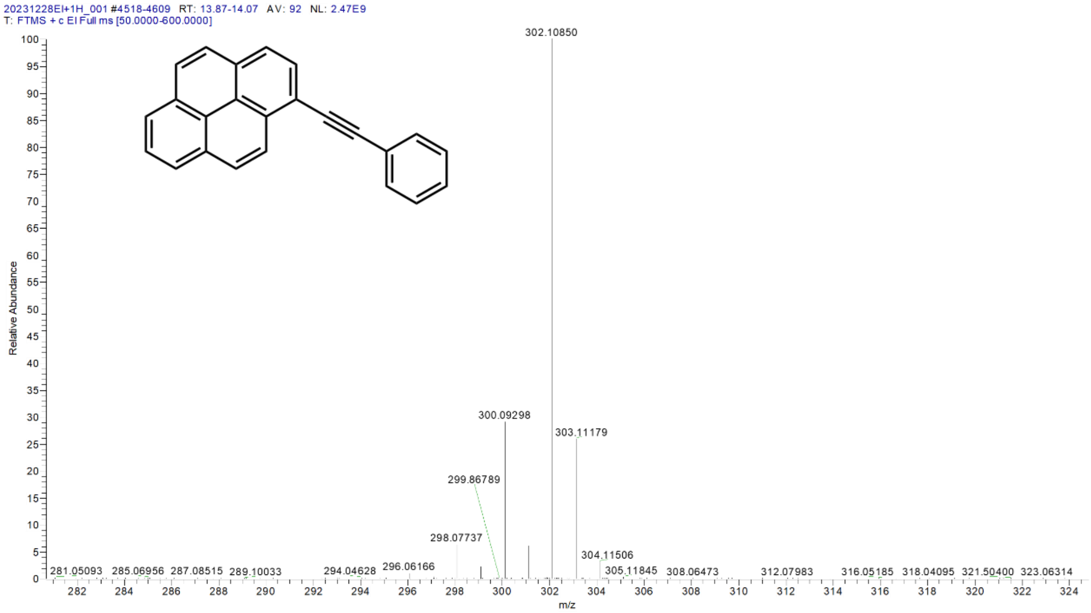

Figure S27. EI-MS spectrum of **1-H-Py,** m/z calcd for $C_{24}H_{14}$: 302.10955 $[M]^{+\cdot}$, found: 302.10850.

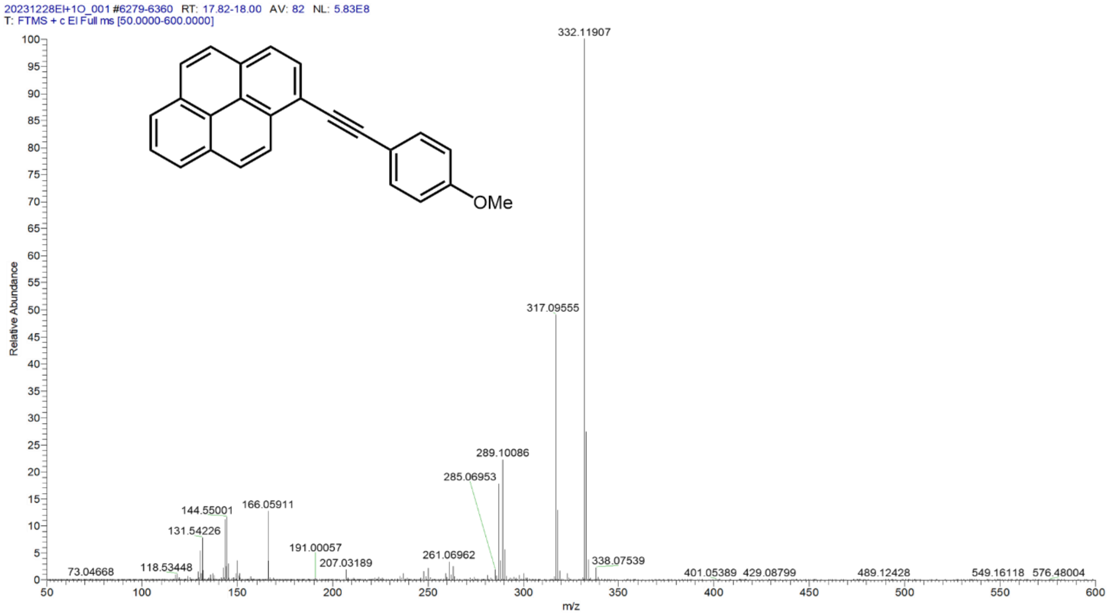

Figure S28. EI-MS spectrum of **1-OME-Py**, m/z calcd for $C_{25}H_{16}O$: 332.12012 $[M]^{+\cdot}$, found: 332.11907.

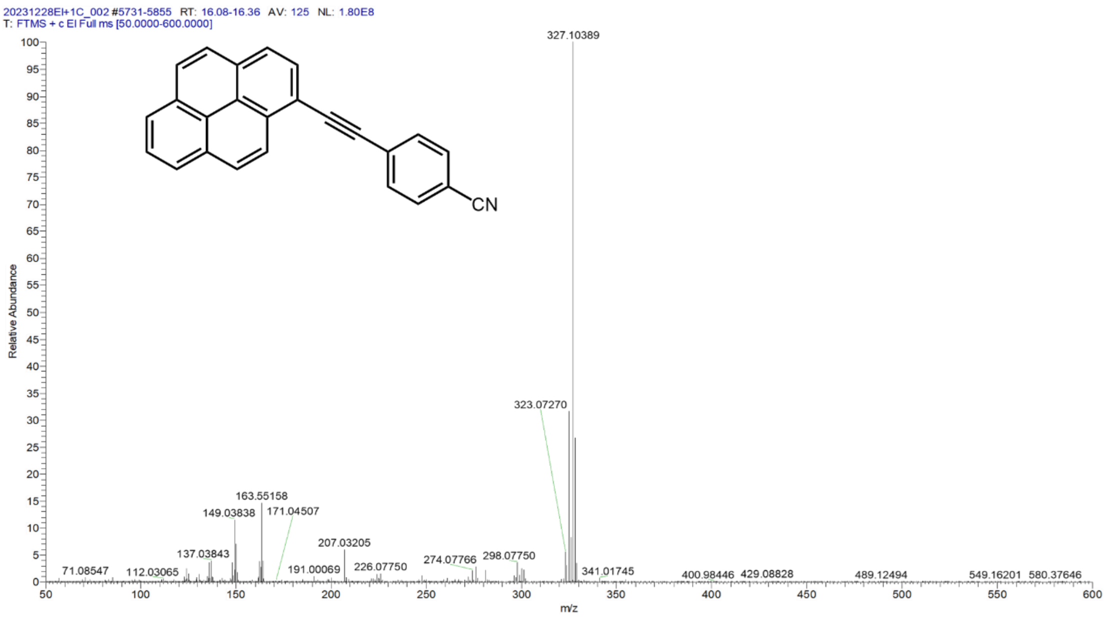

Figure S29. EI-MS spectrum of **1-CN-Py**, m/z calcd for $C_{25}H_{13}N$: 327.10480 $[M]^{+\cdot}$, found: 327.10389.

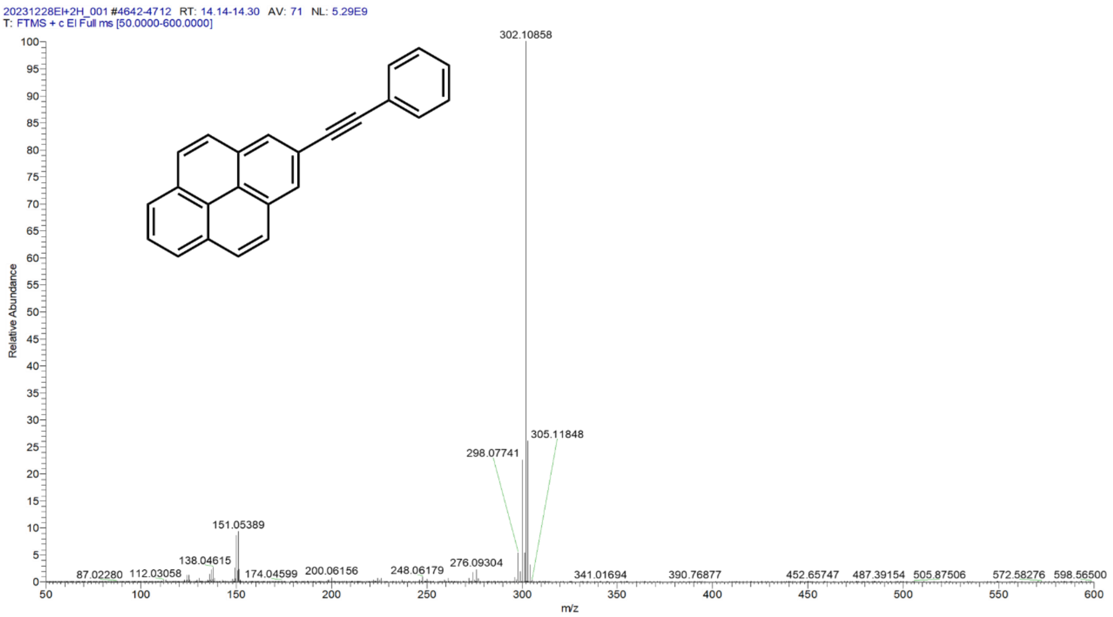

Figure S30. EI-MS spectrum of **2-H-Py**, m/z calcd for $C_{24}H_{14}$: 302.10955 $[M]^{+\cdot}$, found: 302.10858.

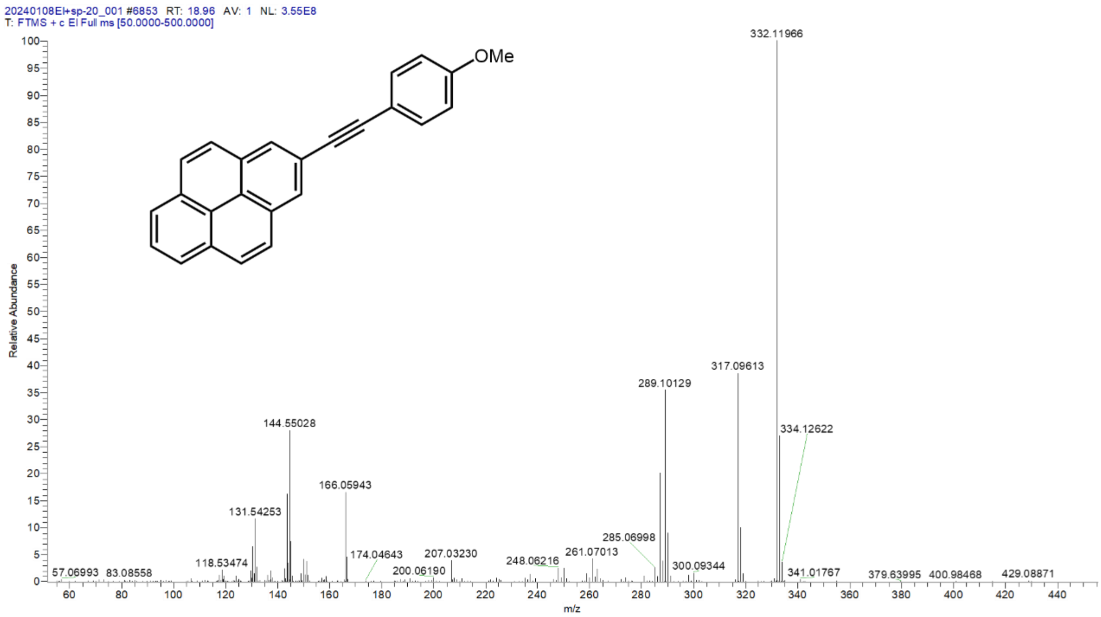

Figure S31. EI-MS spectrum of **2-OME-Py**, m/z calcd for $C_{25}H_{16}O$: 332.12012 $[M]^{+\cdot}$, found: 332.11966.

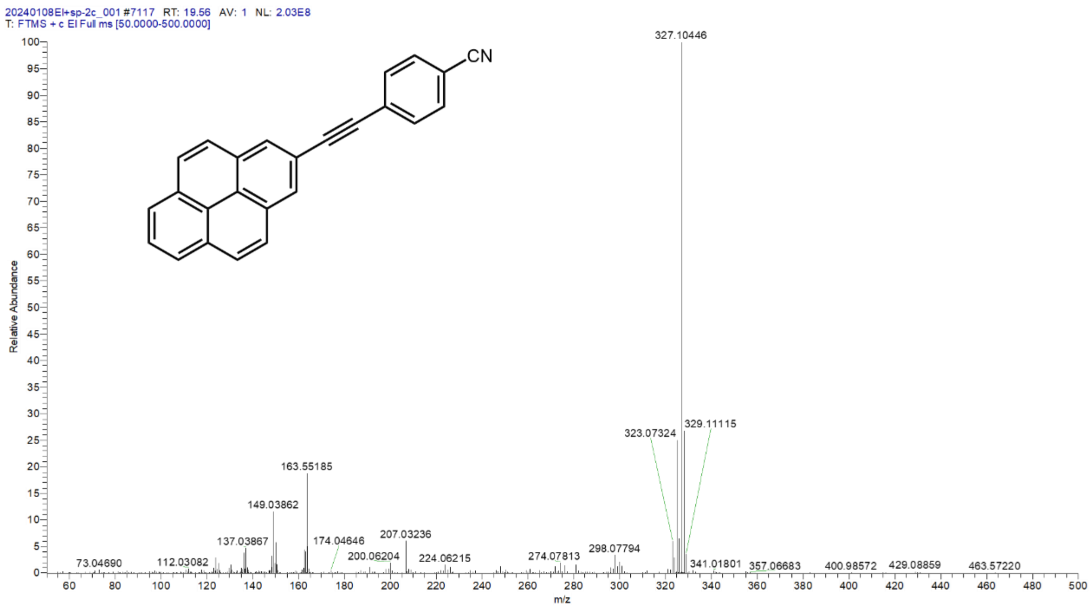

Figure S32. EI-MS spectrum of **2-CN-Py**, m/z calcd for $C_{25}H_{13}N$: 327.10480 $[M]^{+\cdot}$, found: 327.10446.

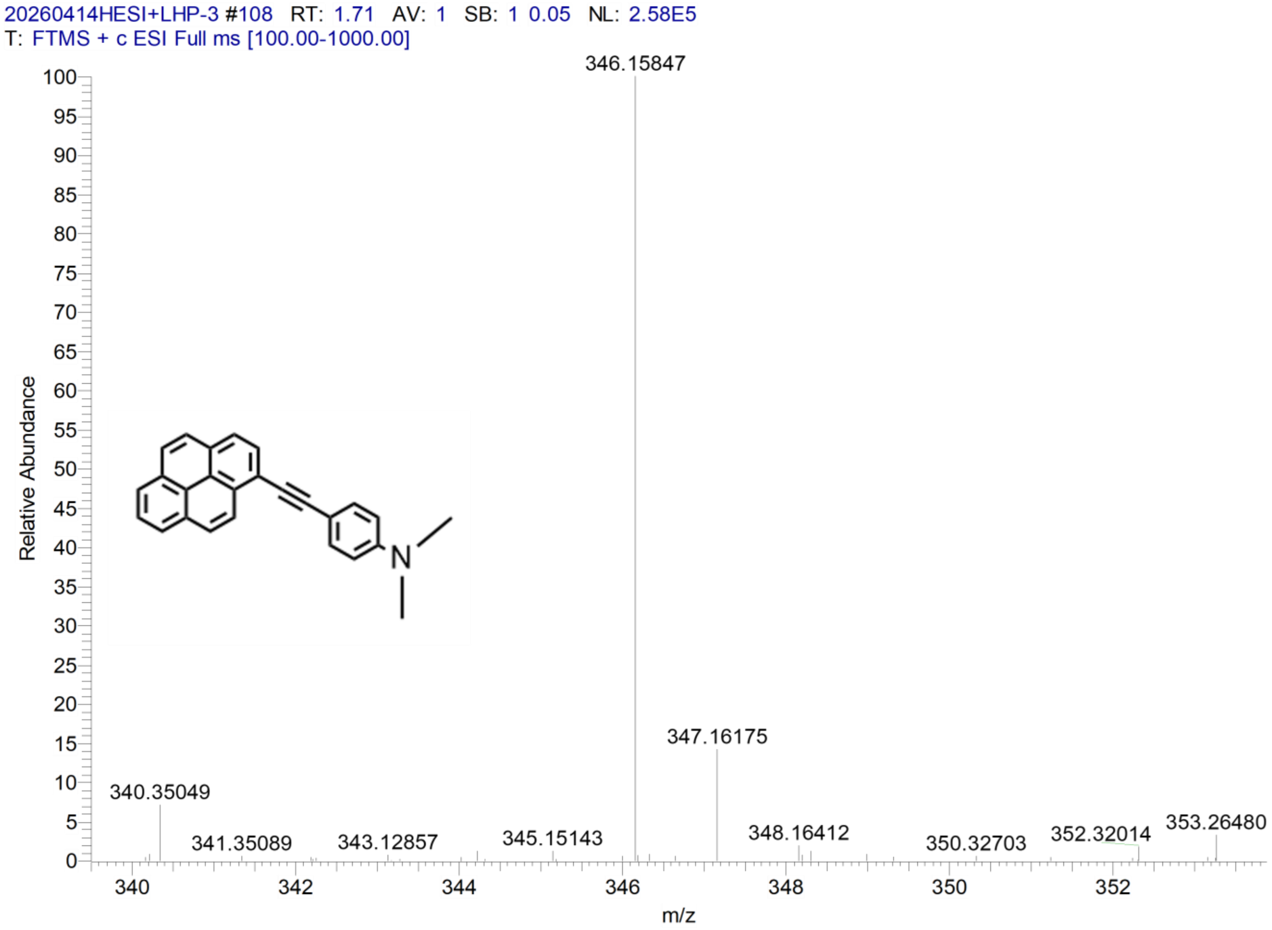

Figure S33. ESI-MS spectrum of **1-N(Me)$_2$-Py,** m/z calcd: 346.1551 [M+H]$^+$, found: 346.15847.

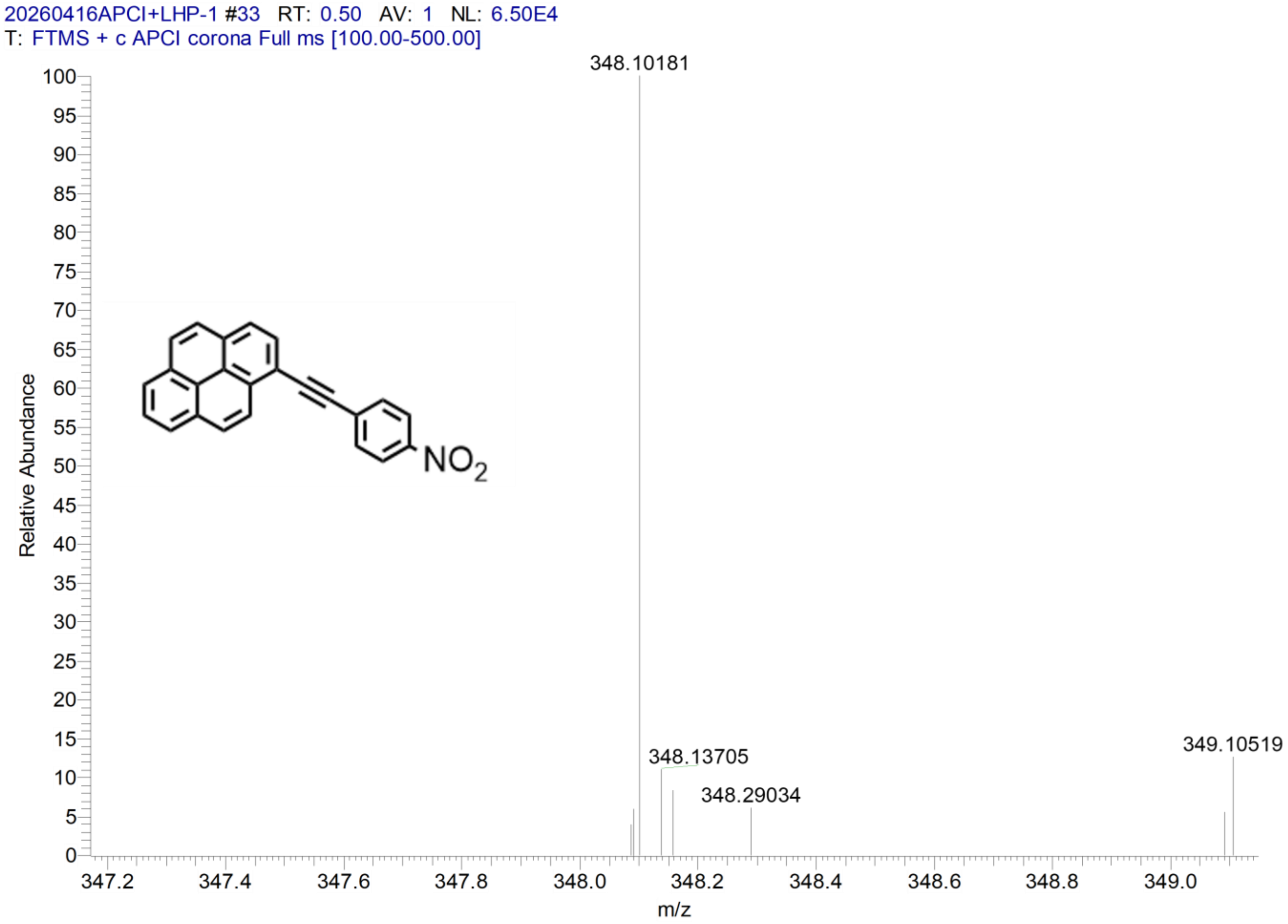

Figure S34. ESI-MS spectrum of **1-NO_2-Py,** m/z calcd: 348.0980 $[M+H]^+$, found: 348.10181.

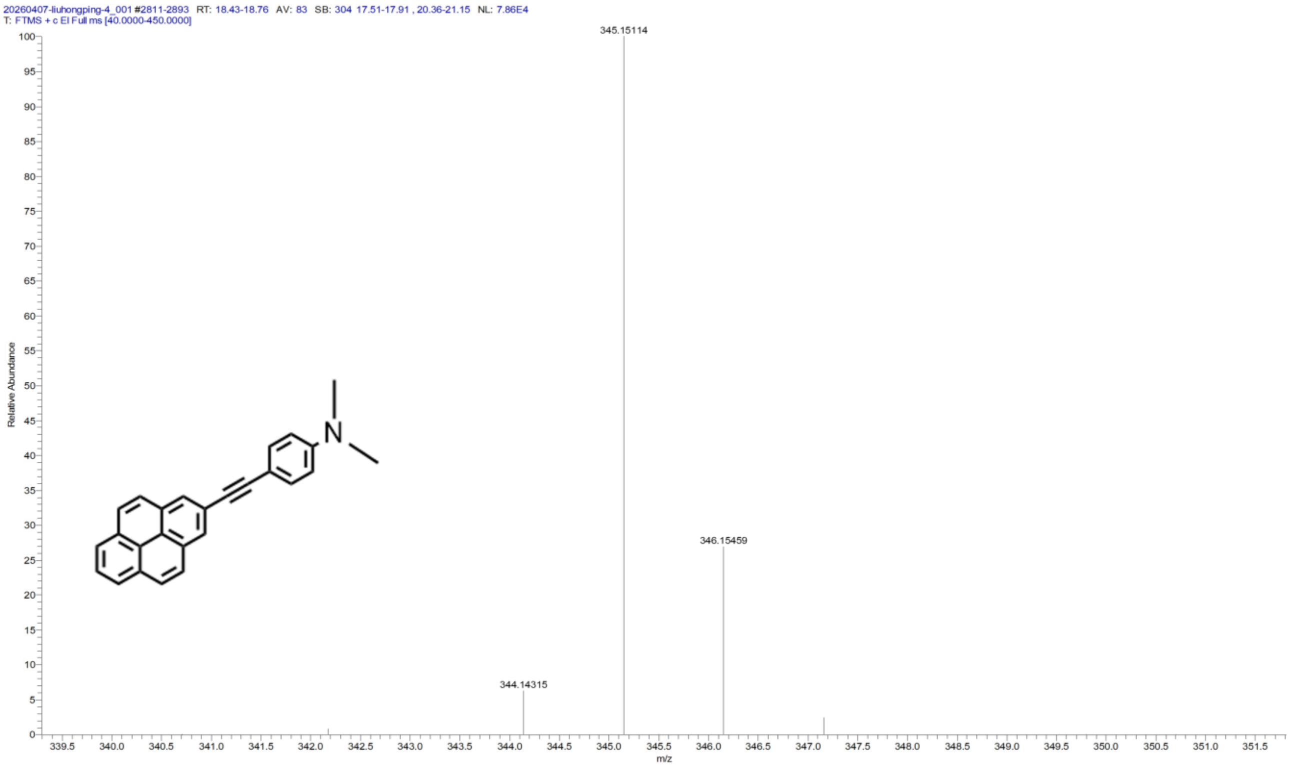

Figure S35. EI-MS spectrum of **2-N(Me)$_2$-Py,** m/z calcd: 345.1517 $[M]^{+\cdot}$, found: 345.15114.

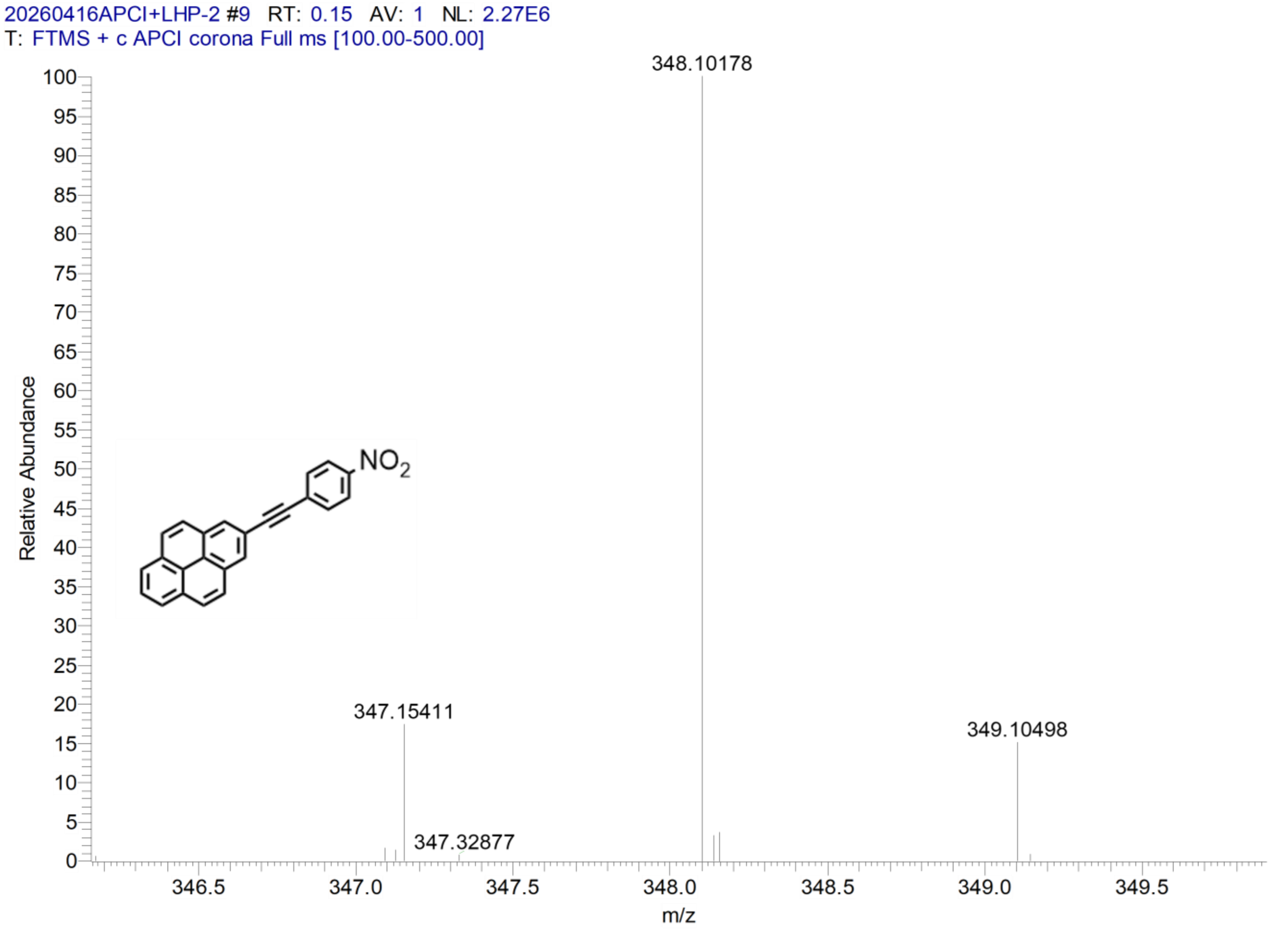

Figure S36. ESI-MS spectrum of **2-NO$_2$-Py,** m/z calcd: 348.0980 [M+H]$^+$, found: 348.10178.

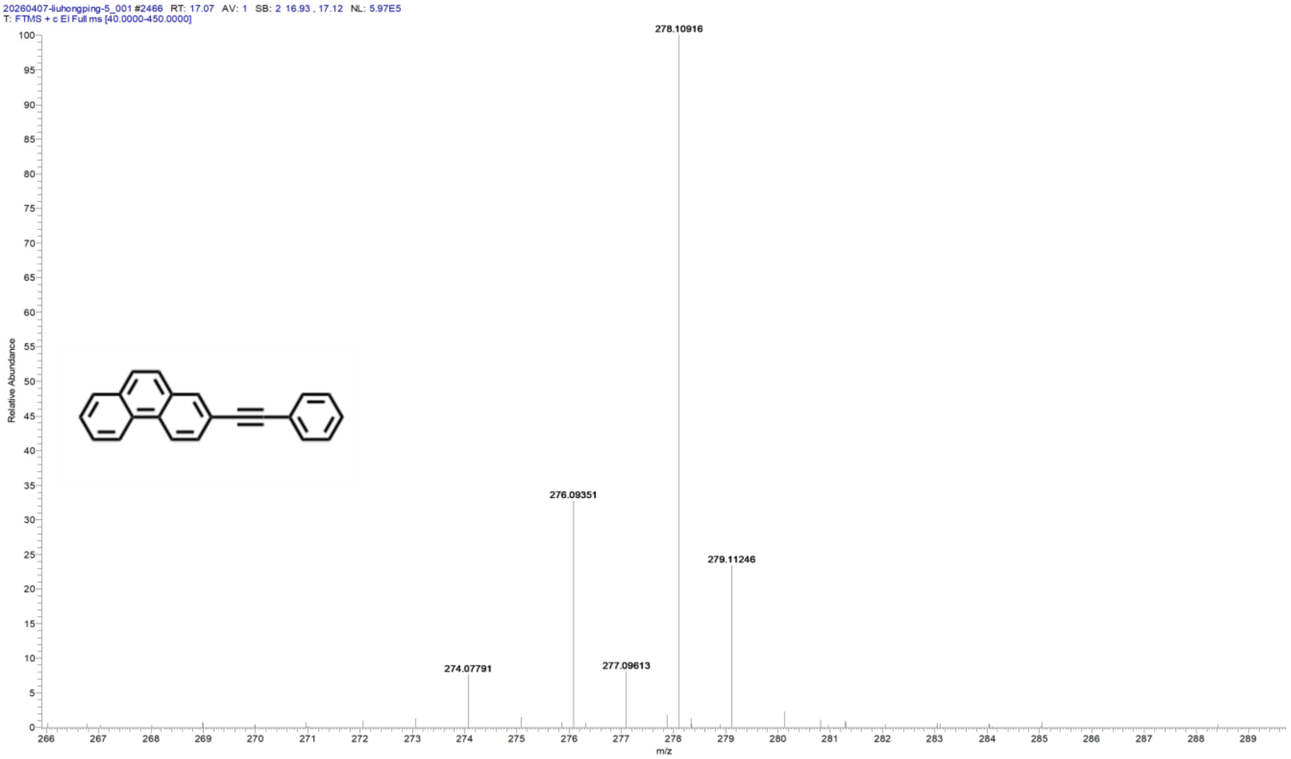

Figure S37. EI-MS spectrum of **2-H-Phen,** m/z calcd: 278.1096 [M]+·, found: 278.10916.

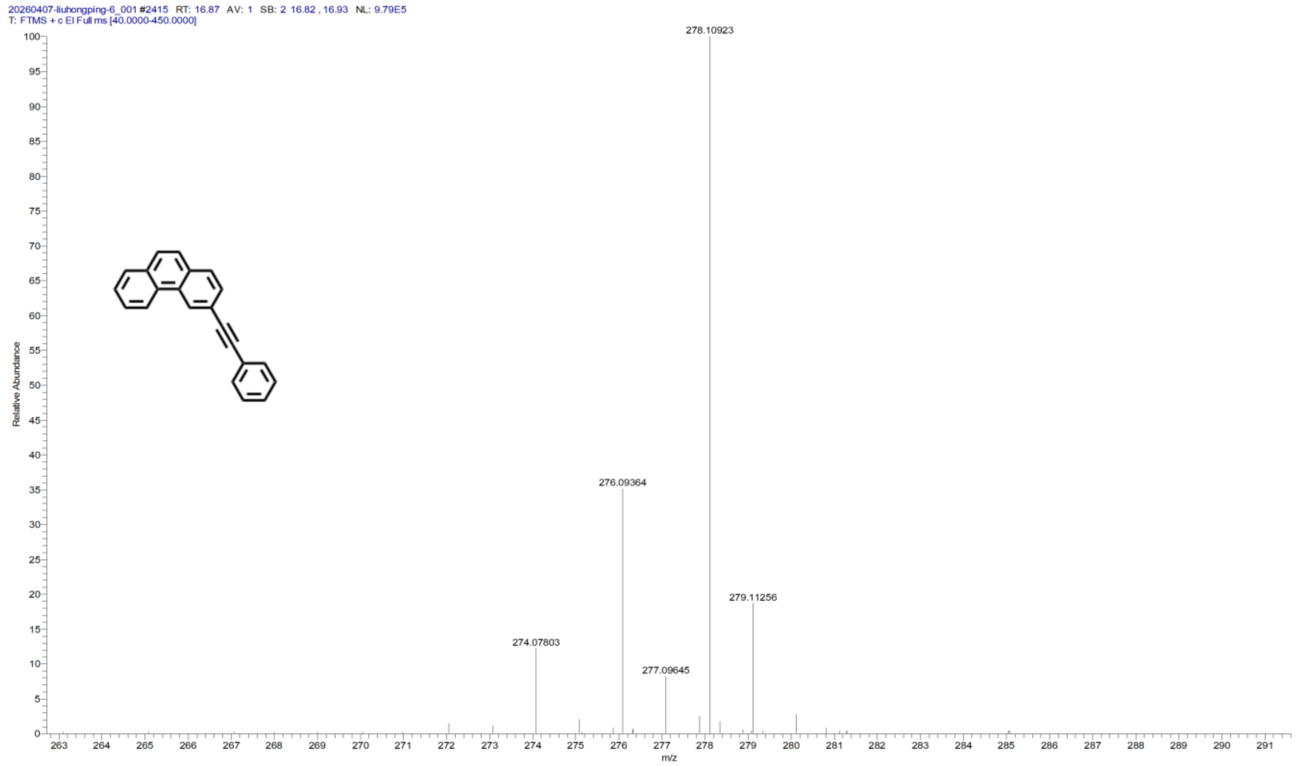

Figure S38. EI-MS spectrum of **3-H-Phen,** m/z calcd: 278.1096 [M]$^{+\cdot}$, found: 278.10923.

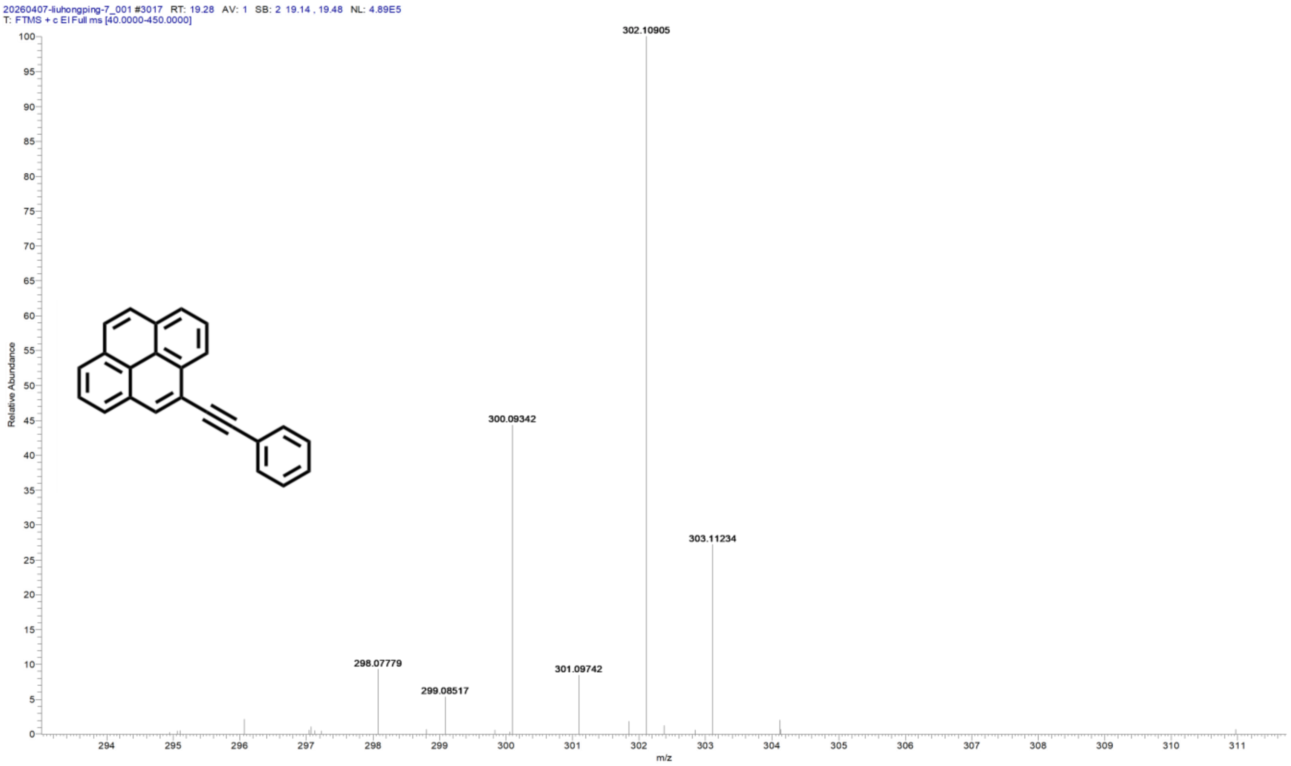

Figure S39. EI-MS spectrum of **4-H-Py,** m/z calcd: 302.1096.1096 [M]$^{+\cdot}$, found: 302.10905.

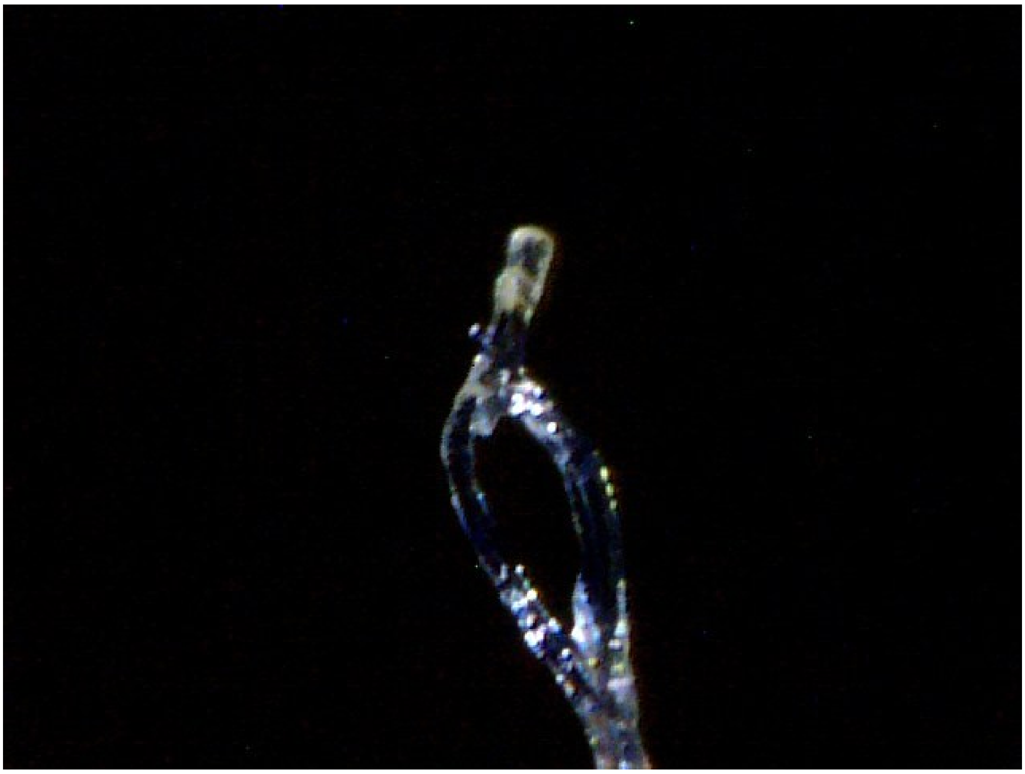

Figure S40. Single crystal sample of **1-H-Py** (obtained from acetonitrile solution).

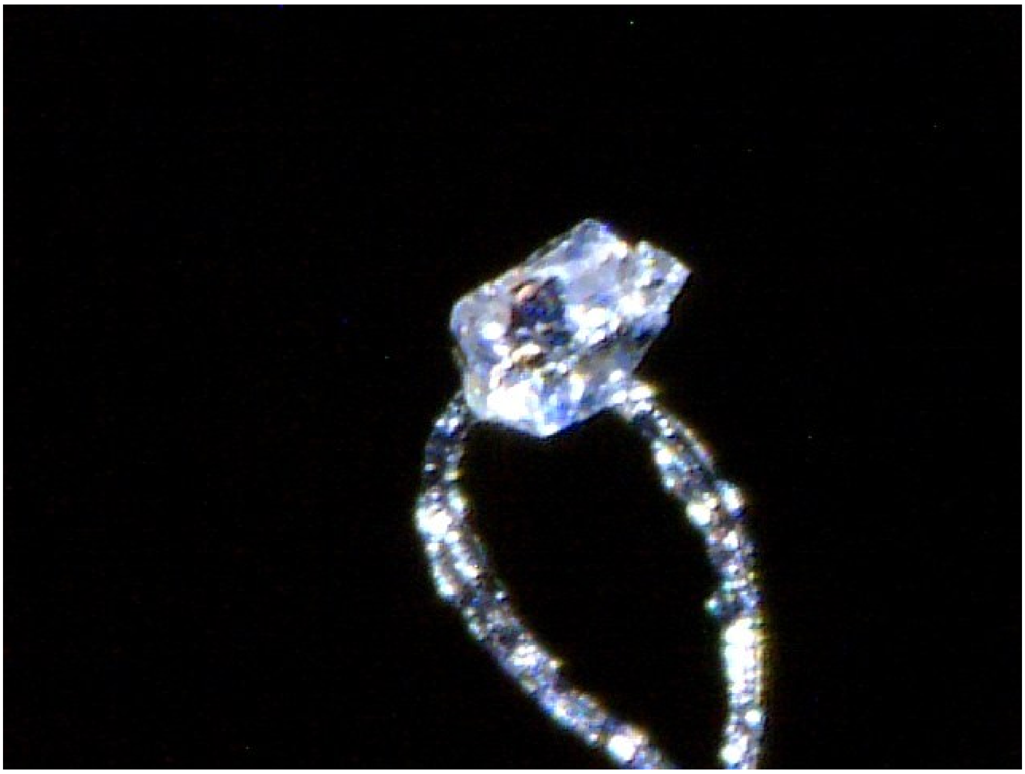

Figure S41. Single crystal sample of **2-H-Py** (obtained from acetonitrile solution).

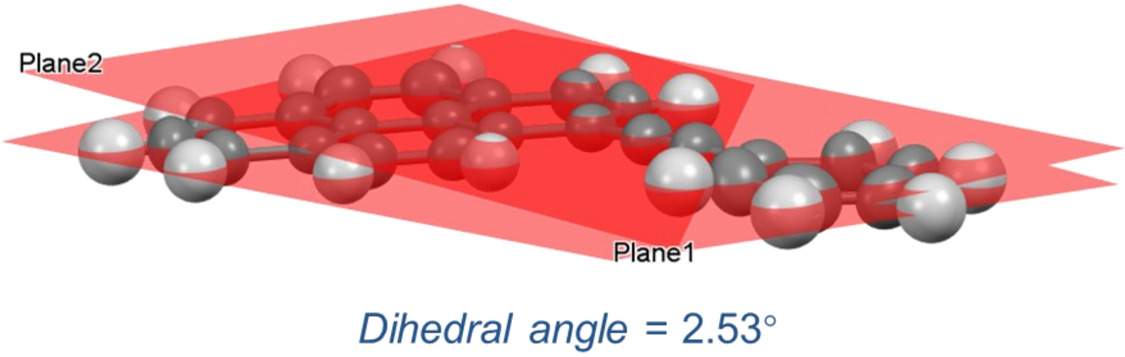

Figure S42. SCXRD structure of **1-H-Py**.

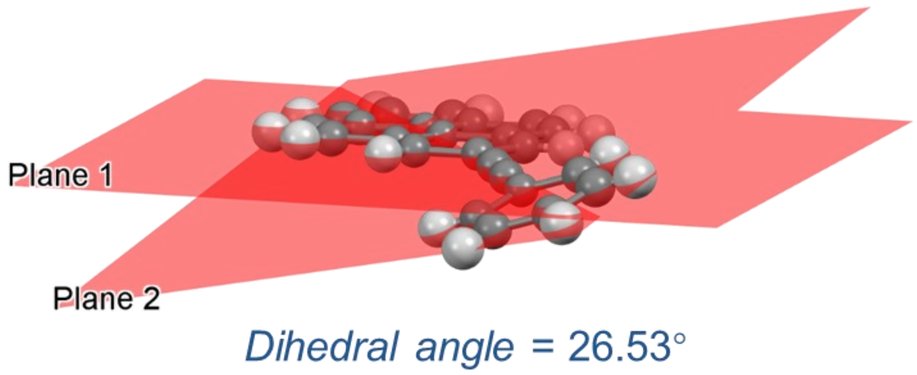

Figure S43. SCXRD structure of **2-H-Py**.

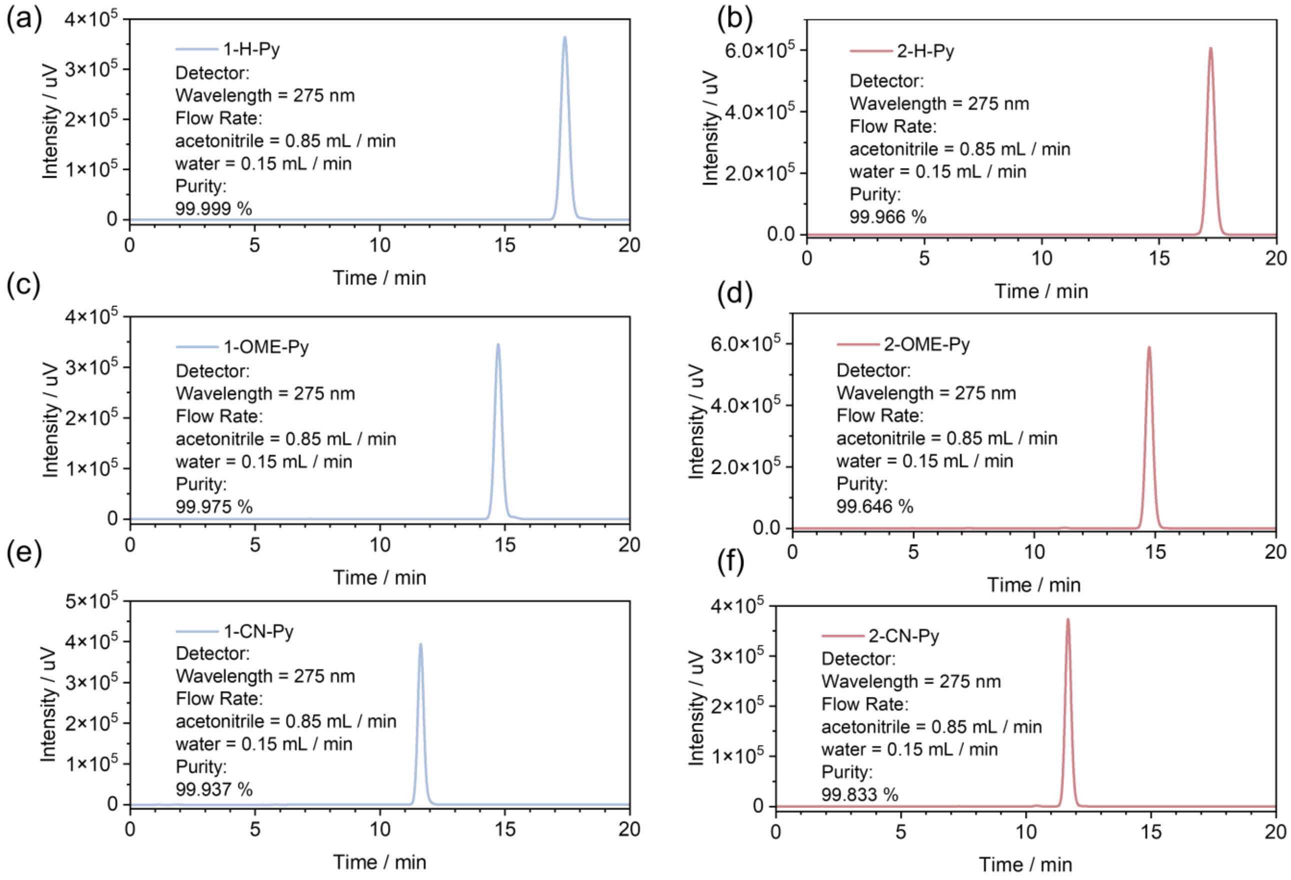

Figure S44. HPLC data of pyrene derivatives studied in this work. (a) **1-H-Py**; (b) **2-H-Py**; (c) **1-OME-Py**; (d) **2-OME-Py**; (e) **1-CN-Py**; (f) **2-CN-Py**.

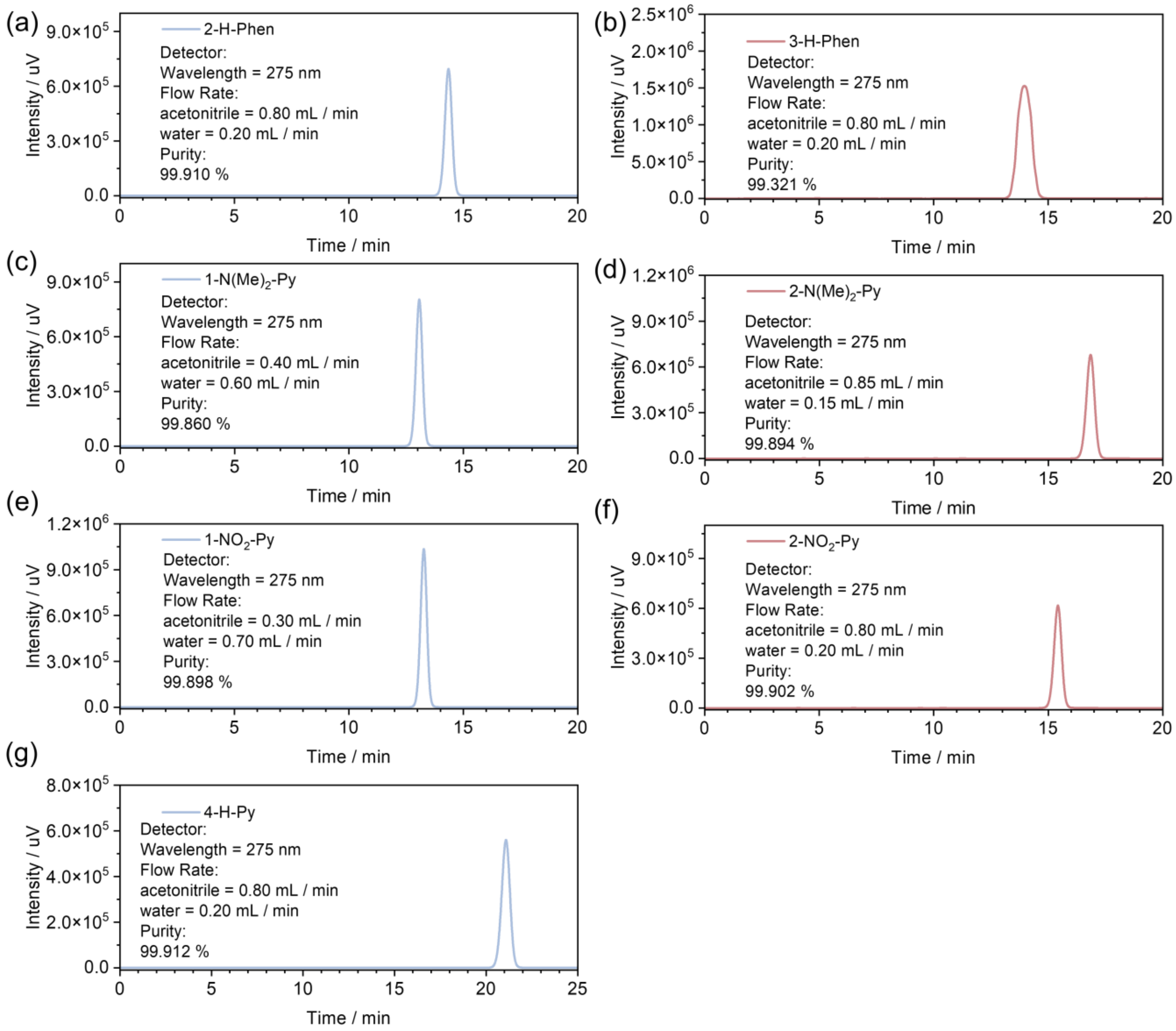

Figure S45. HPLC data of pyrene derivatives studied in this work. (a) **2-H-Phen**; (b) **3-H-Phen**; (c) **1-N(Me)2-Py**; (d) **2-N(Me)2-Py**; (e) **1-NO2-Py**; (f) **2-NO2-Py**. (g) **4-H-Py**.

Part III. Other Supplementary Figures

Theoretical calculations

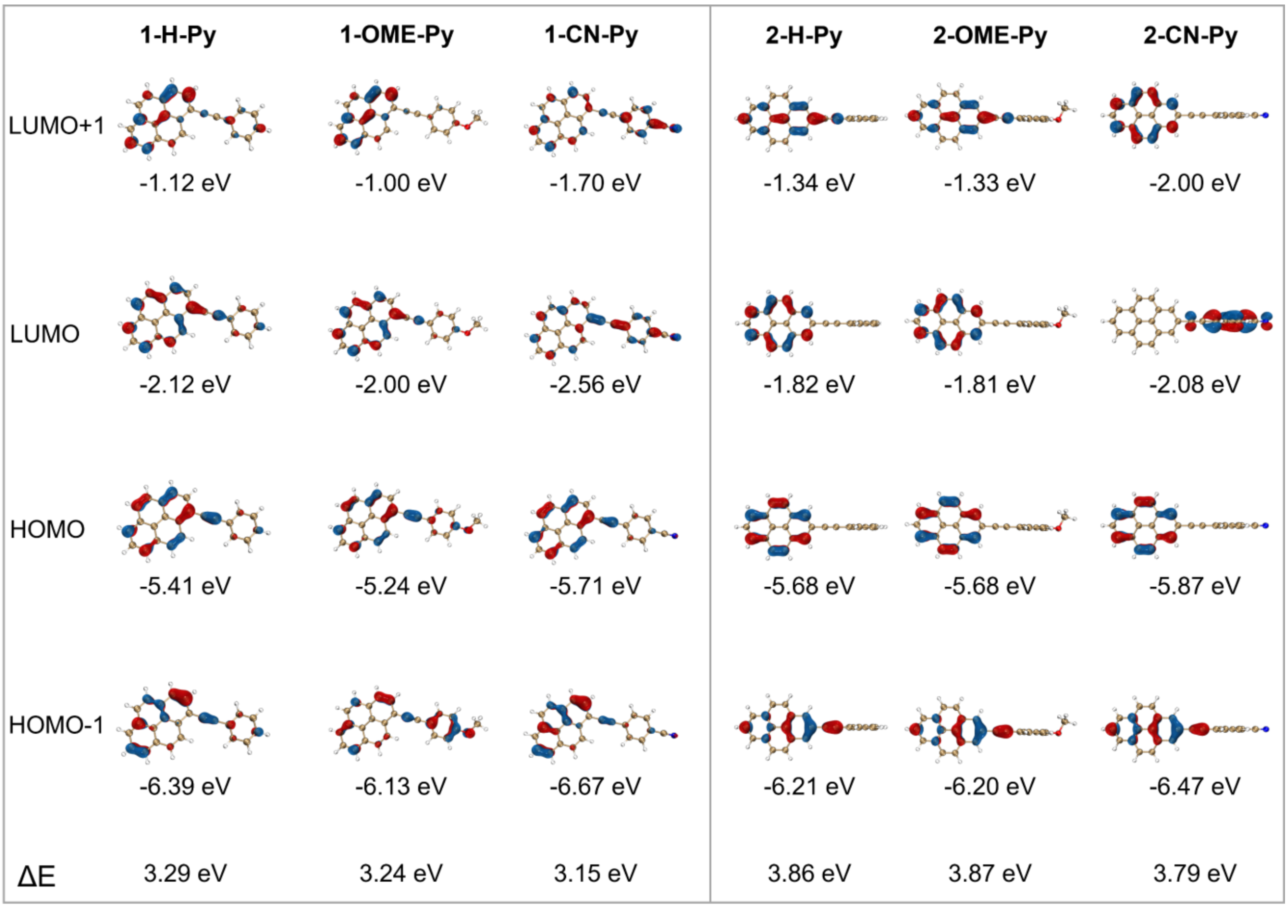

Figure S46. Calculated molecular orbitals and energy levels of 1- and 2-substituted pyrenes.

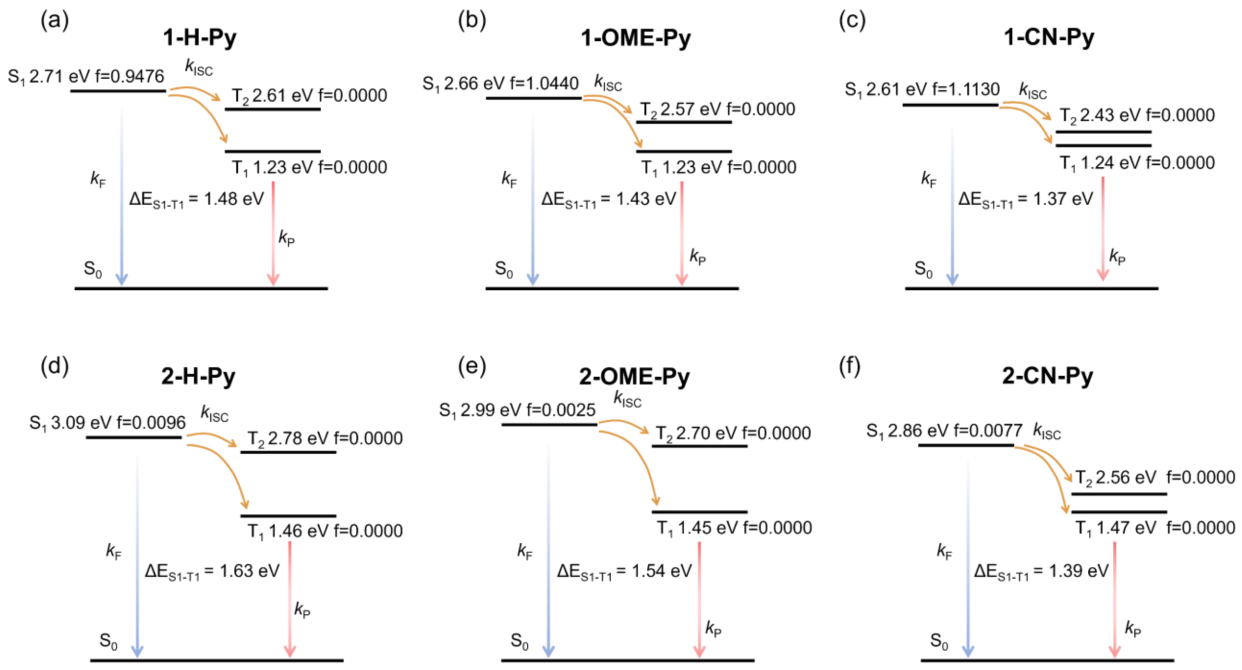

Figure S47. Calculated vertical excitation energies of S_1, T_1, and T_2 states with associated radiative oscillator strengths. (a) **1-H-Py**; (b) **1-OME-Py**; (c) **1-CN-Py**; (d) **2-H-Py**; (e) **2-OME-Py**; (f) **2-CN-Py**. The calculations reveal that 1-substituted pyrenes have lower excited state energies than their 2-substituted analogues due to extend p-conjugation at the anti-nodal site. In addition, Furthermore, symmetry-breaking in 1-substituted pyrenes leads to a significantly stronger $S_1 \rightarrow S_0$ oscillator strength (*f*) compared to 2-substituted pyrenes. Notably, the calculated *f* values for triplet excited states are zero because spin-orbital coupling (SOC) is not included in the standard Gaussian TD-DFT method; experimentally, these triplet radiative transitions are activated via weak SOC.

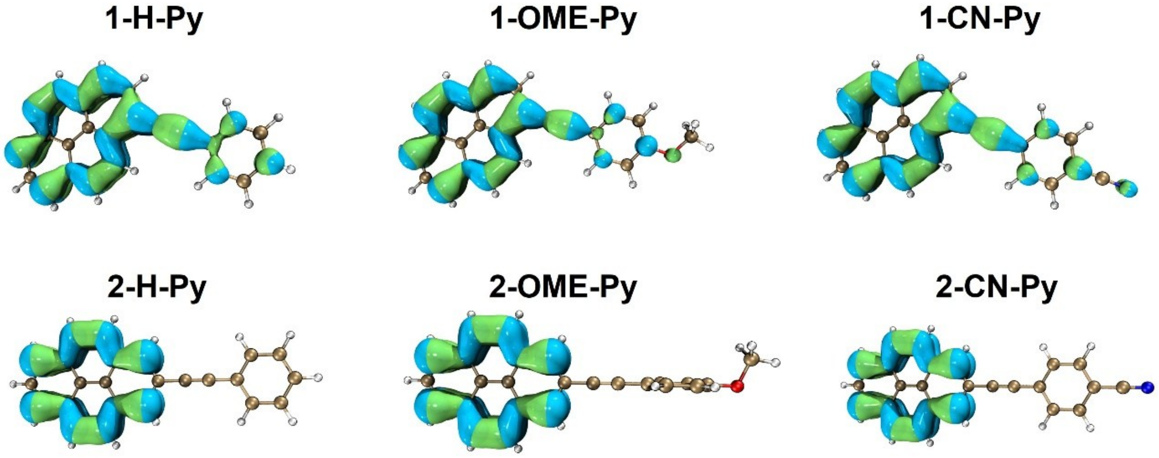

Figure S48. Hole–electron analysis of 1- and 2-substituted pyrenes in the T_1 state at ωB97XD/def2TZVP level (isovalue: 0.001). Blue and green surfaces denote hole and electron, respectively.

Spectral data in solution

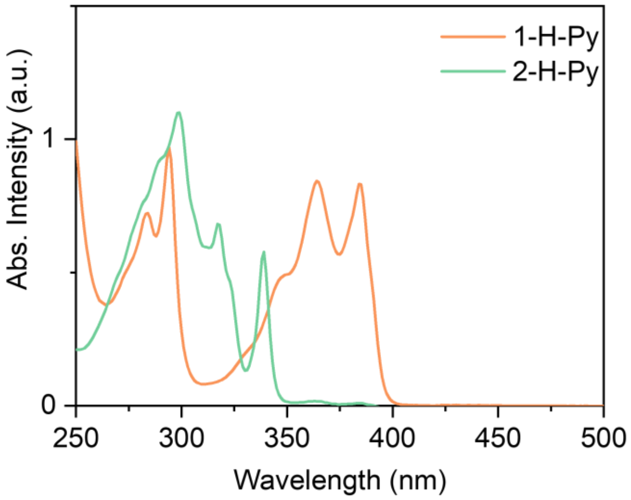

Figure S49. UV-vis absorption spectra of **1-H-Py** and **2-H-Py** in DCM (c = 1 × 10^{-5} M). Compared to **2-H-Py**, **1-H-Py** shows a red-shifted absorption profile with additional bands at 365 and 385 nm. This is because the anti-nodal substitution in **1-H-Py** breaks molecular symmetry and extends the FMO distribution.

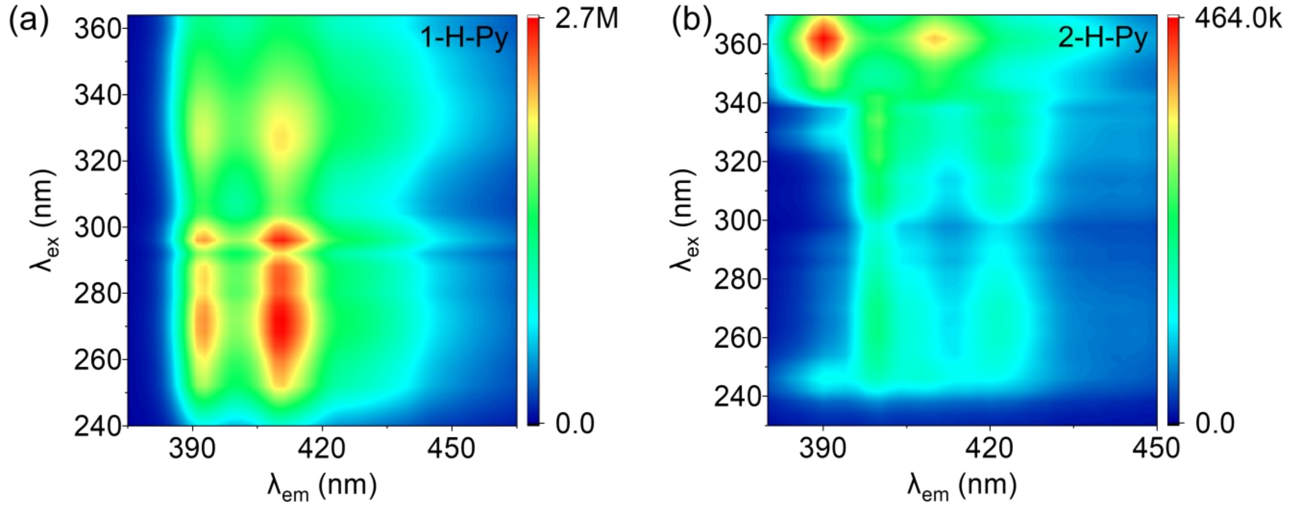

Figure S50. Steady-state excitation–emission maps of (a) **1-H-Py** and (b) **2-H-Py** in 2-MeTHF solution at 298 K (c = 1 × 10^{-5} M). **1-H-Py** exhibits multiple distinct excitation bands and a slightly red-shifted excitation profile compared to **2-H-Py**. This occurs because the anti-nodal substitution in **1-H-Py** breaks molecular symmetry and delocalizes the FMOs, which lowers the excitation energy and generates additional allowed singlet excitation pathways.

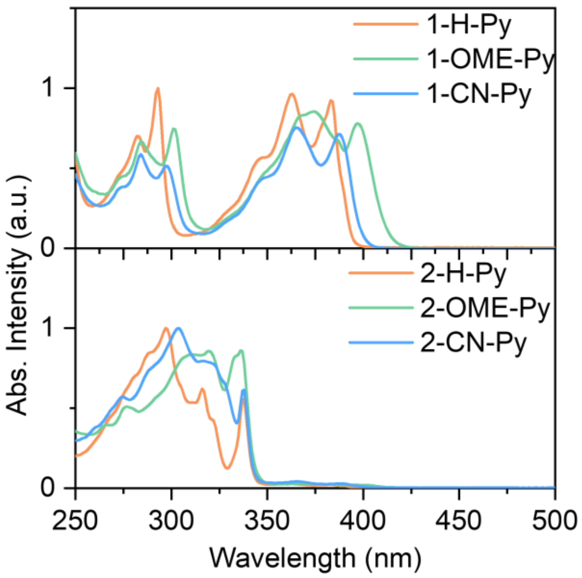

Figure S51. (a) UV–vis absorption of pyrene derivatives in 2-MeTHF recorded at 298 K (c = 1 × 10^{-5} mol/L).

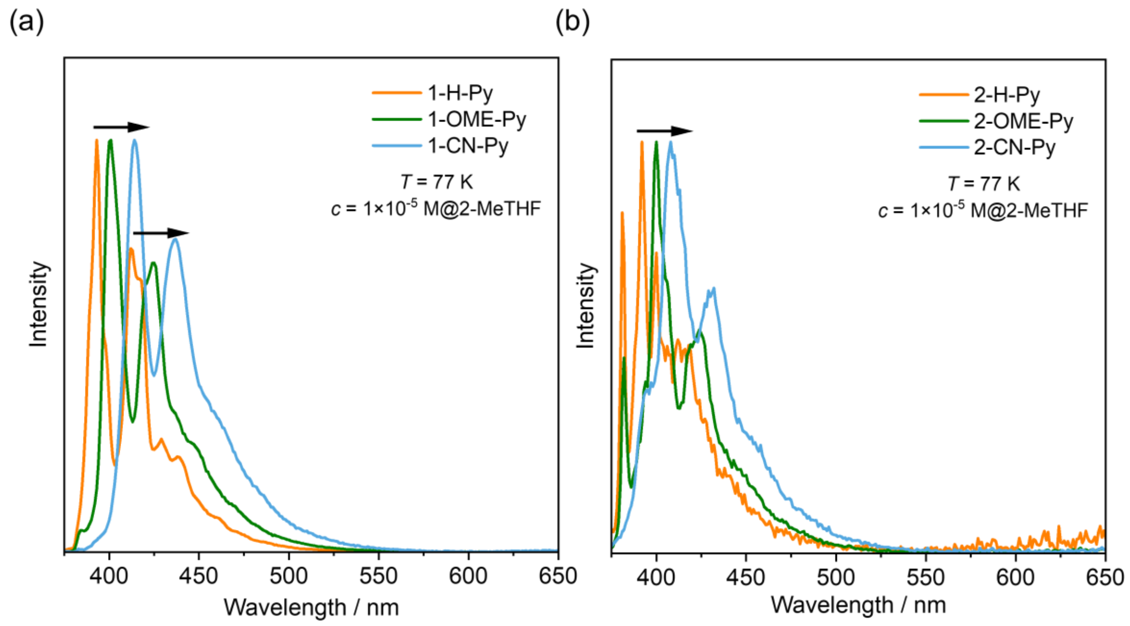

Figure S52. (a-b) Steady-state PL spectra of pyrene derivatives in 2-MeTHF recorded at 77 K (c = 1 × 10^{-5} mol/L). (a) **1-H-Py**, **1-OME-Py** and **1-CN-Py**; (b) **2-H-Py**, **2-OME-Py** and **2-CN-Py**.

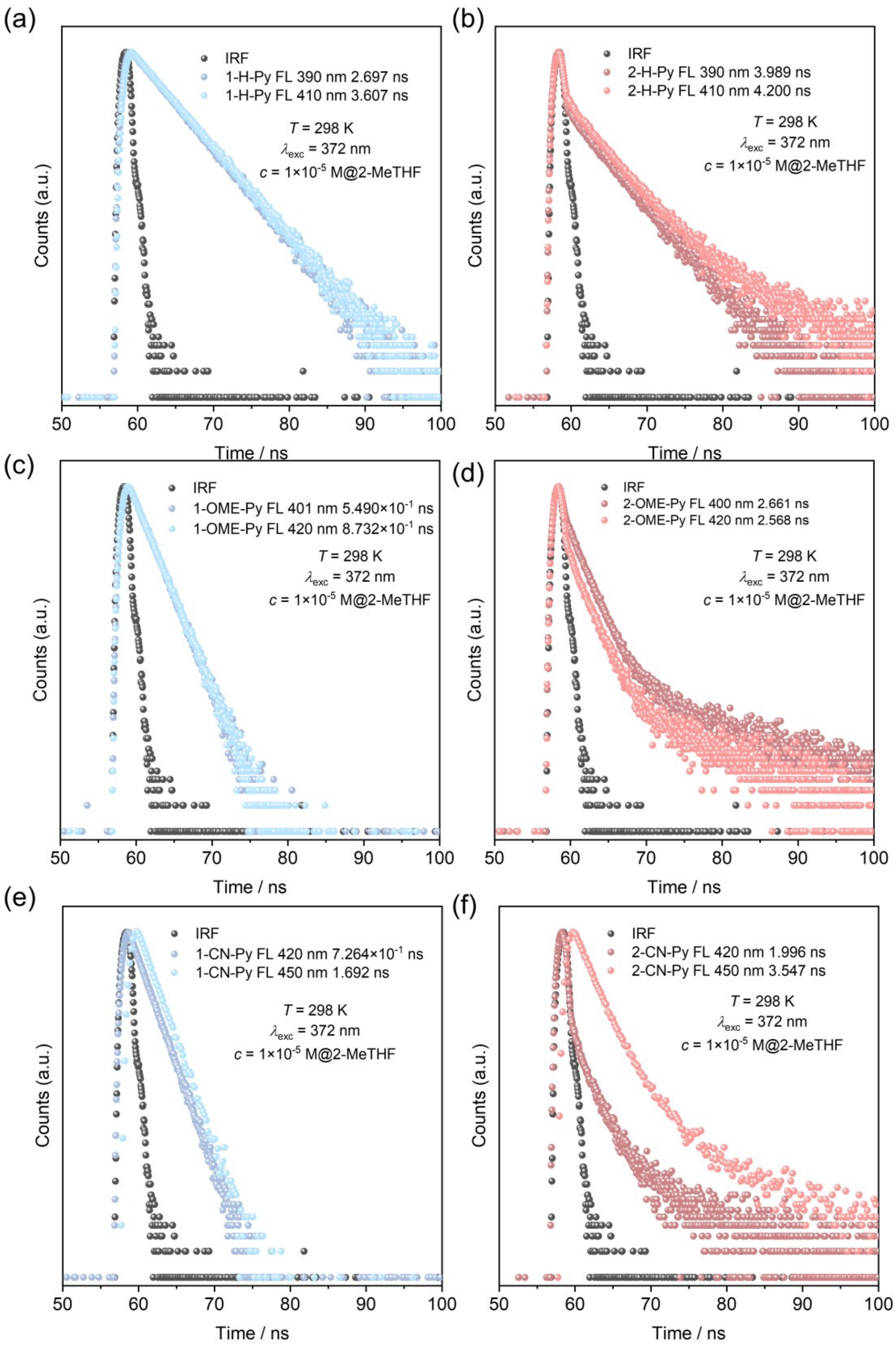

Figure S53. Time-resolved fluorescence decay profiles of pyrene derivatives recorded at associated emission peaks in 2-MeTHF at 298 K (λ_{exc} = 372 nm; c = 1 × 10^{-5} mol/L). (a) **1-H-Py**; (b) **2-H-Py**; (c) **1-OME-Py**; (d) **2-OME-Py**; (e) **1-CN-Py**; (f) **2-CN-Py**.

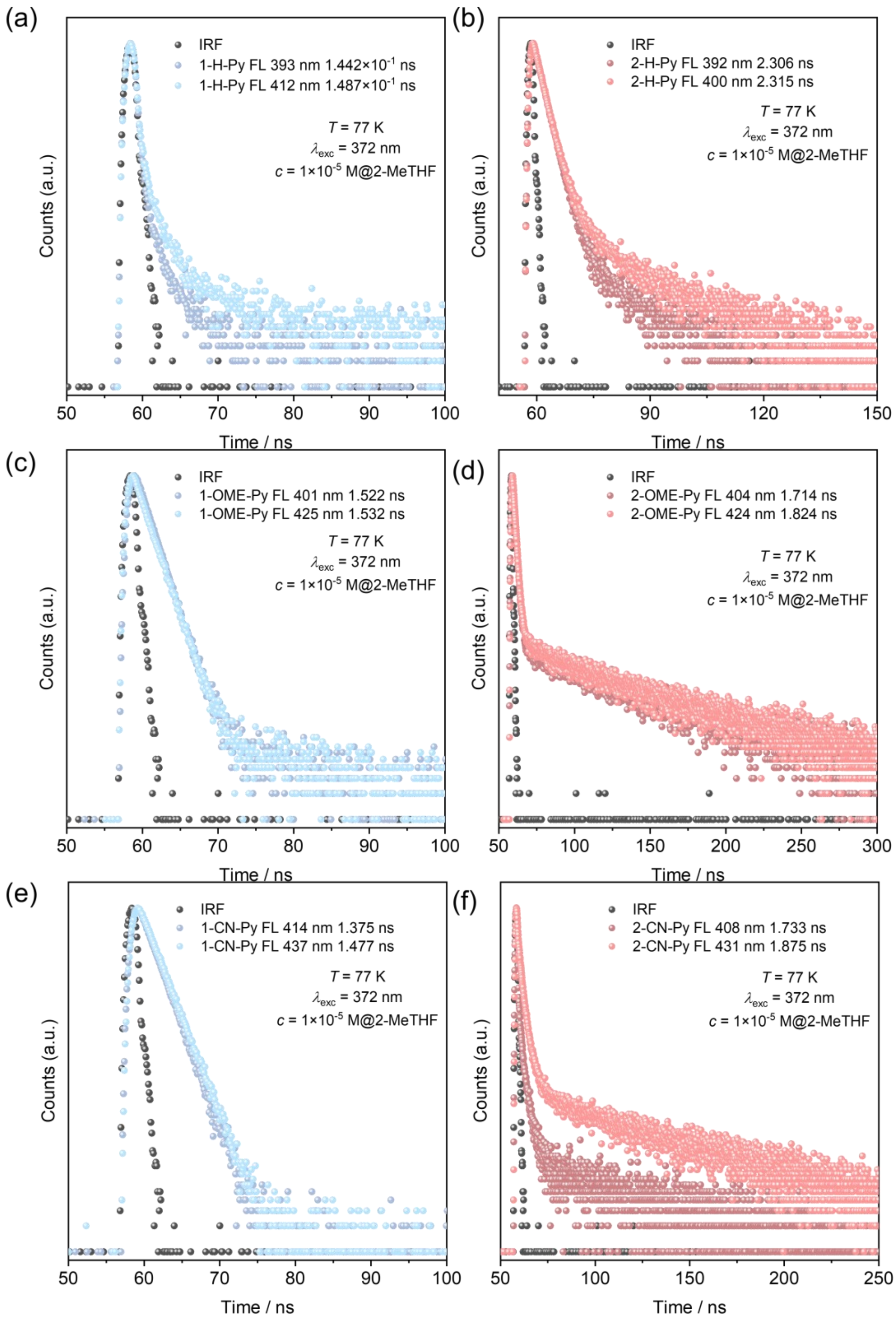

Figure S54. Time-resolved decay curves and lifetime values of pyrene derivatives recorded at associated emission peaks in 2-MeTHF at 77 K (λ_{exc} = 372 nm; c = 1 × 10^{-5} mol/L). (a) **1-H-Py**; (b) **2-H-Py**; (c) **1-OME-Py**; (d) **2-OME-Py**; (e) **1-CN-Py**; (f) **2-CN-Py**.

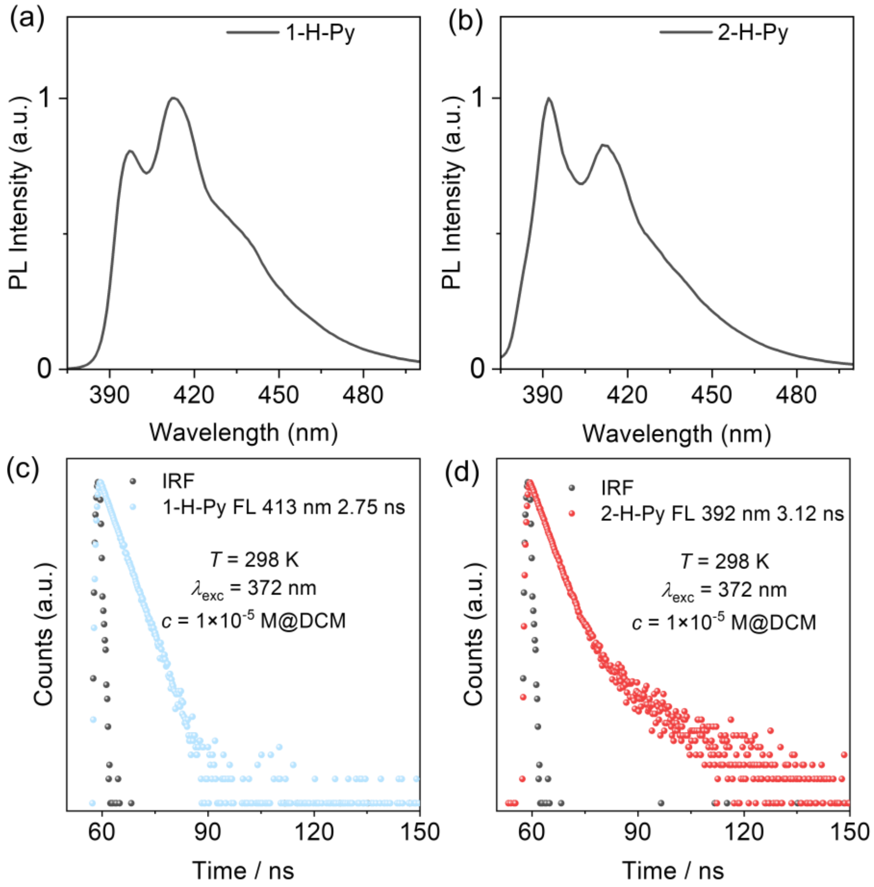

Figure S55. Steady-state PL spectra of (a) **1-H-Py** and (b) **2-H-Py** in DCM at 298 K (λ_{exc} = 350 nm. Time-resolved fluorescence decay profiles of (c) **1-H-Py** and (d) **2-H-Py** in DCM at 298 K (λ_{exc} = 372 nm). c = 1 × 10^{-5} mol/L.

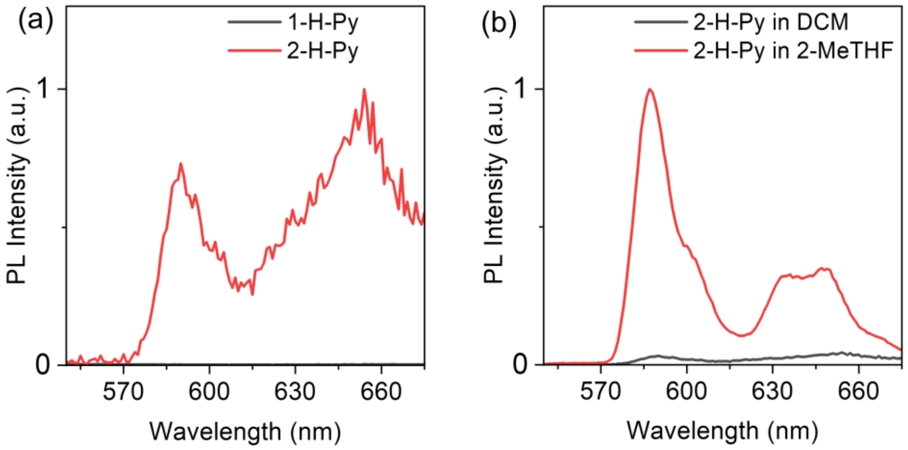

Figure S56. (a) Delayed PL spectra of **1-H-Py** and **2-H-Py** recorded in DCM at 77 K. (b) Delayed PL spectra of **2-H-Py** recorded in different solvents at 77 K. Gated time: 5–100 ms; λ_{exc} = 298 nm; c = 1 × 10^{-5} mol/L.

As expected, **1-H-Py** exhibits no detectable phosphorescence in DCM at 77 K, whereas **2-H-Py** displays pronounced phosphorescence centered at 592 and 655 nm (**Figure S56a**). Notably, the phosphorescence intensity of **2-H-Py** at 77 K is significantly weaker in DCM than in 2-MeTHF. This is because highly crystalline DCM at 77 K excludes the chromophores from the solvent lattice, leading to aggregation-caused quenching (ACQ). In contrast, the glassy 2-MeTHF matrix uniformly confines and isolates the individual chromophores, preventing ACQ and facilitating obvious phosphorescence (**Figure S56b**).

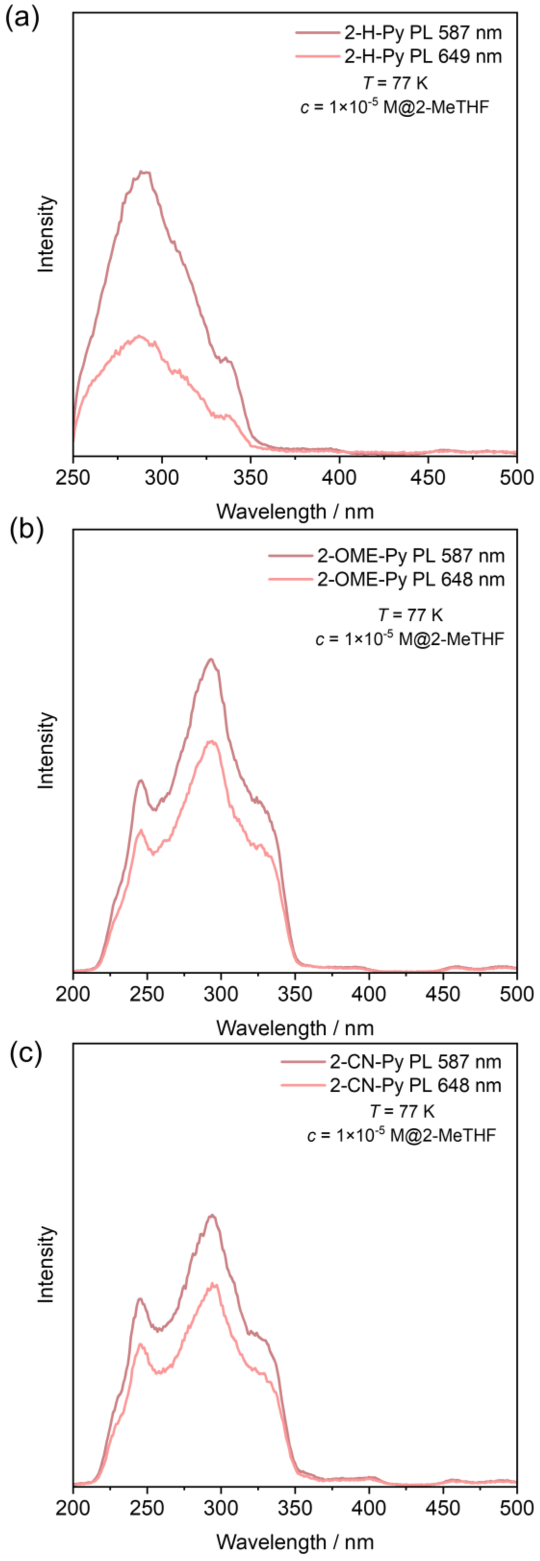

Figure S57. Time-gated excitation spectra (gated time = 5 ms) of 2-substituted pyrene derivatives recorded at emission maxima (indicated in the figure) in 2-MeTHF at 298 K (c = 1×10^{-5} mol/L). (a) **2-H-Py**; (b) **2-OME-Py**; (c) **2-CN-Py**.

Spectral data in PMMA

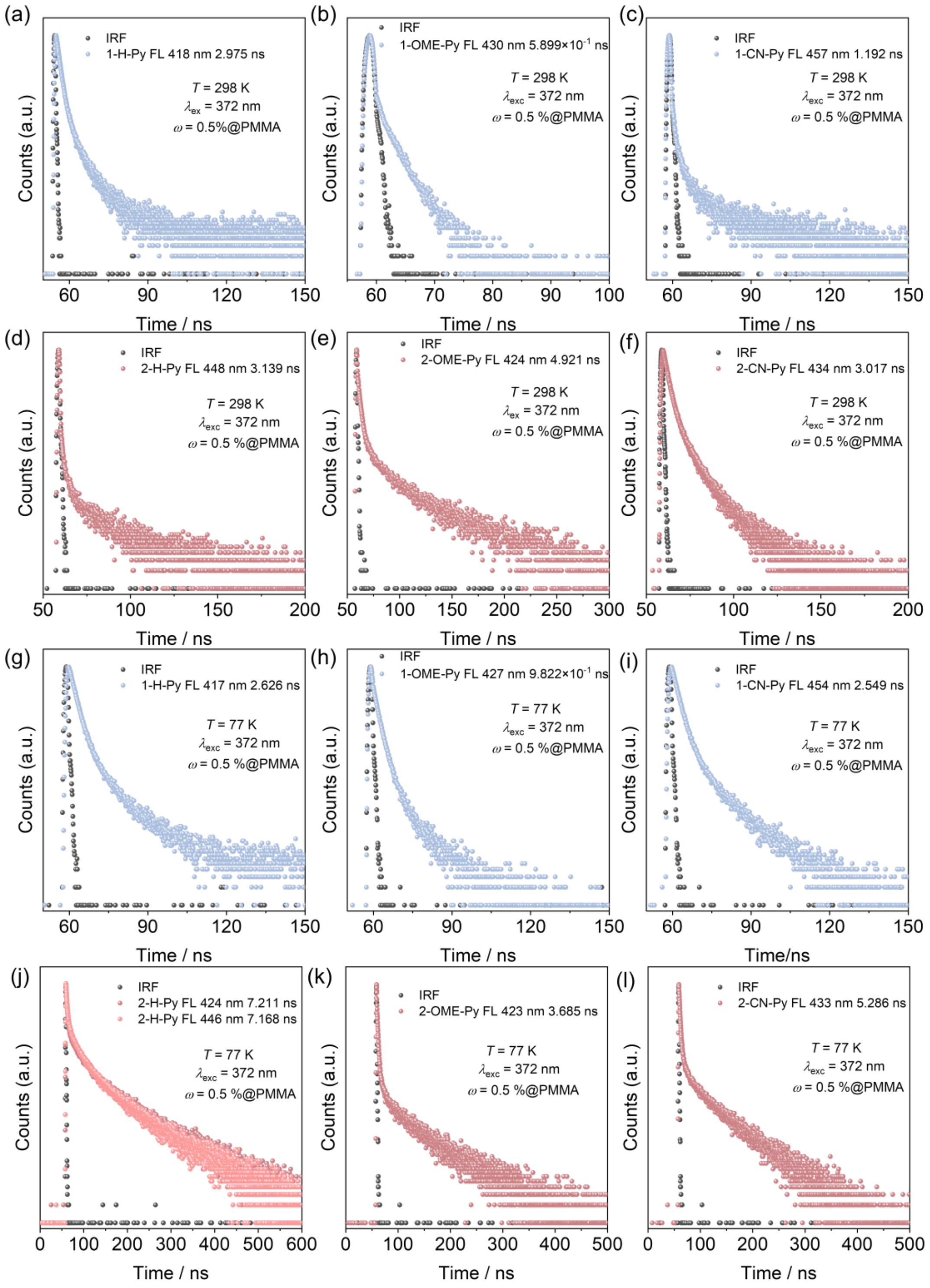

Figure S58. (a-f) Time-resolved decay curves and lifetime values of 0.5 wt% pyrene derivatives in PMMA films recorded at emission peaks at 298 K (λ_{exc} = 372 nm). (g-l) Time-resolved decay curves and lifetime values of 0.5 wt% pyrene derivatives in PMMA films recorded at emission peaks at 77 K (λ_{exc} = 372 nm).

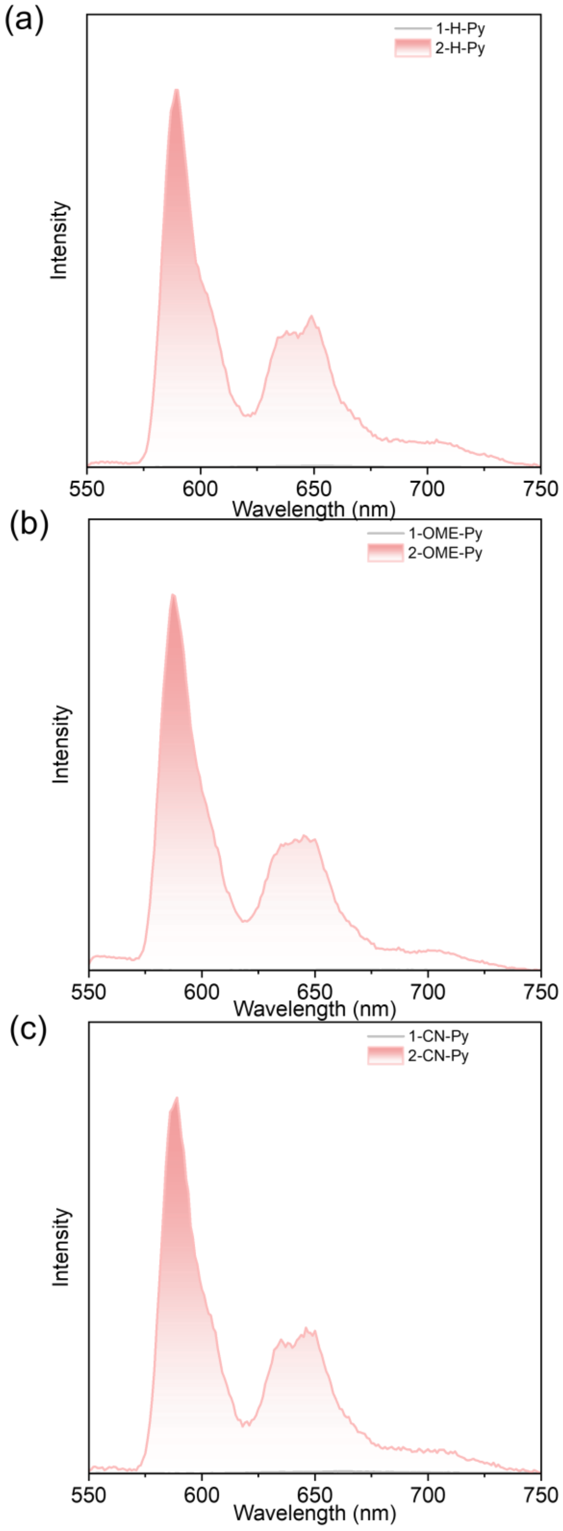

Figure S59. Delayed PL spectra of **2-H-Py**, **2-OME-Py**, and **2-CN-Py** in PMMA at 77 K (gated time: 5 ms; λ_{exc} = 298 nm); doping concentration: 0.5 wt%. Note that no observable phosphorescence was recorded in 1-substituted pyrenes (a) **1-H-Py** and **2-H-Py**; (b) **1-OME-Py** and **2-OME-Py**; (c) **1-CN-Py** and **2-CN-Py**.

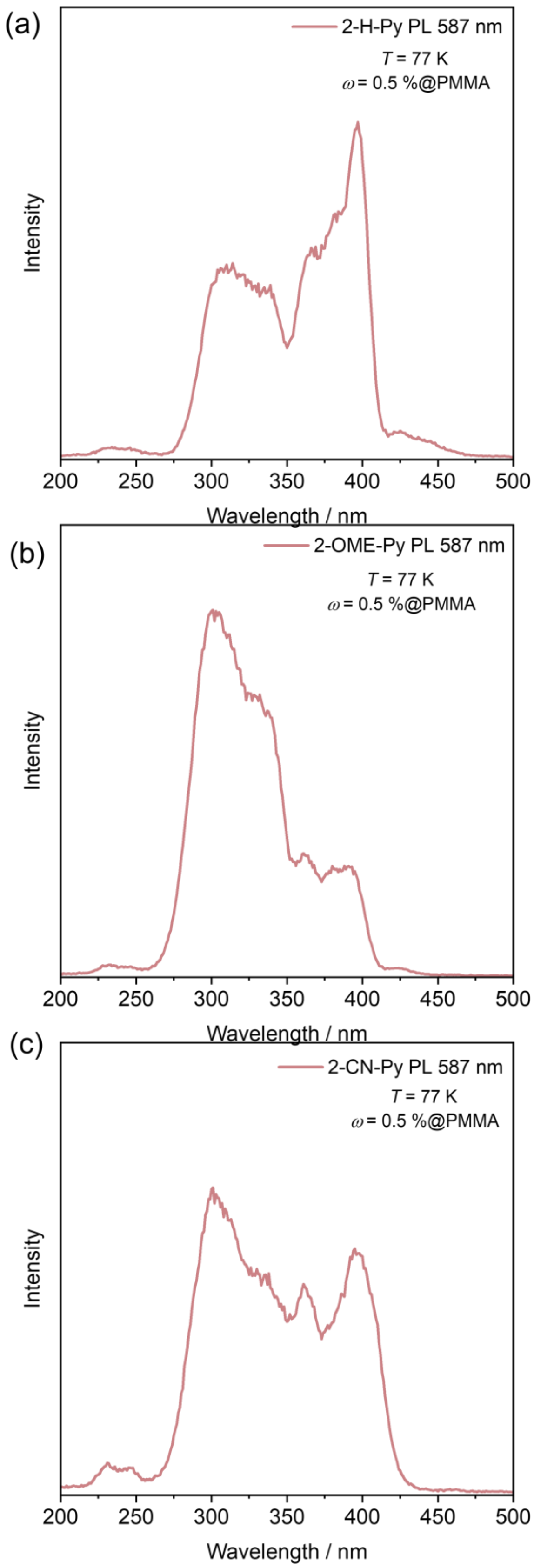

Figure S60. Time-gated excitation spectra (gated time: 5 ms) of 0.5 wt% 2-substituted pyrene derivatives in PMMA recorded at emission maxima (indicated in the figure) at 77 K. (a) **2-H-Py**; (b) **2-OME-Py**; (c) **2-CN-Py**.

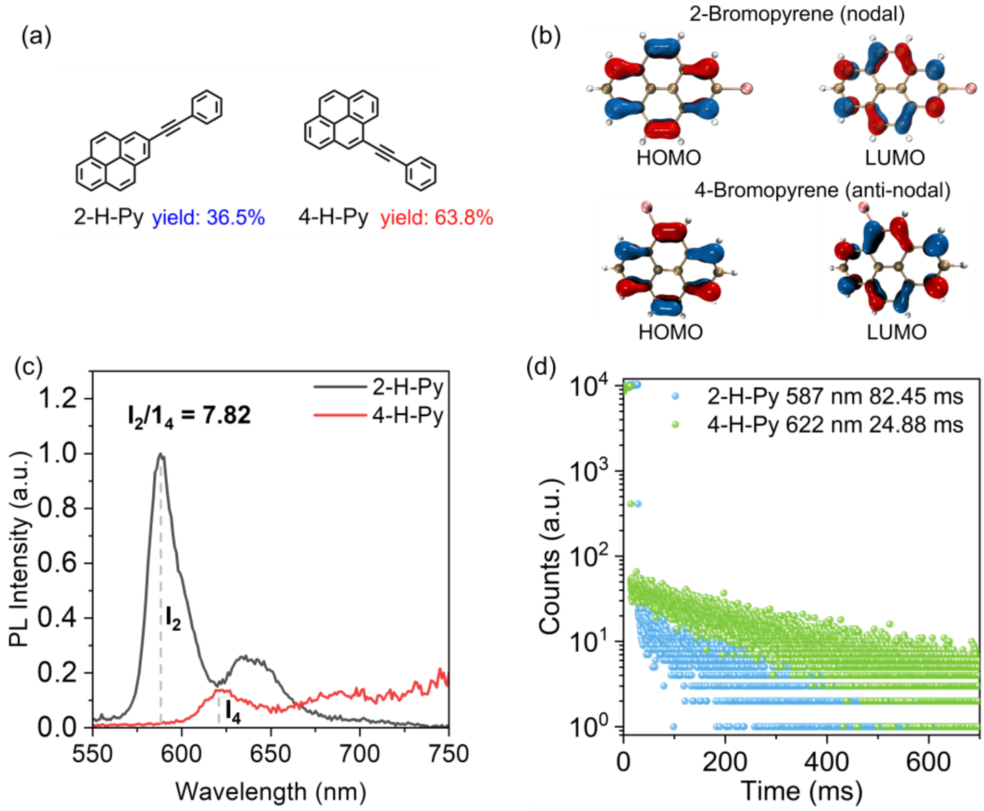

Figure S61. (a) Chemical structures of **2-H-Py** and **4-H-Py** with coupling yields shown below. (b) Calculated FMO distributions of 2-Bromopyrene and 4-Bromopyrene at optimized S_0 geometries. (c) Delayed PL spectra of **2-H-Py** and **4-H-Py** in PMMA recorded at 298 K (doping ratio: 0.5 wt%; λ_{exc} = 298 nm; I_2: phosphorescence intensity of **2-H-Py**; I_4: phosphorescence intensity of **4-H-Py**). (d) Time-resolved phosphorescence decay profiles of **2-H-Py** and **4-H-Py** in PMMA at 298 K (doping ratio: 0.5 wt%; λ_{exc} = 285 nm).

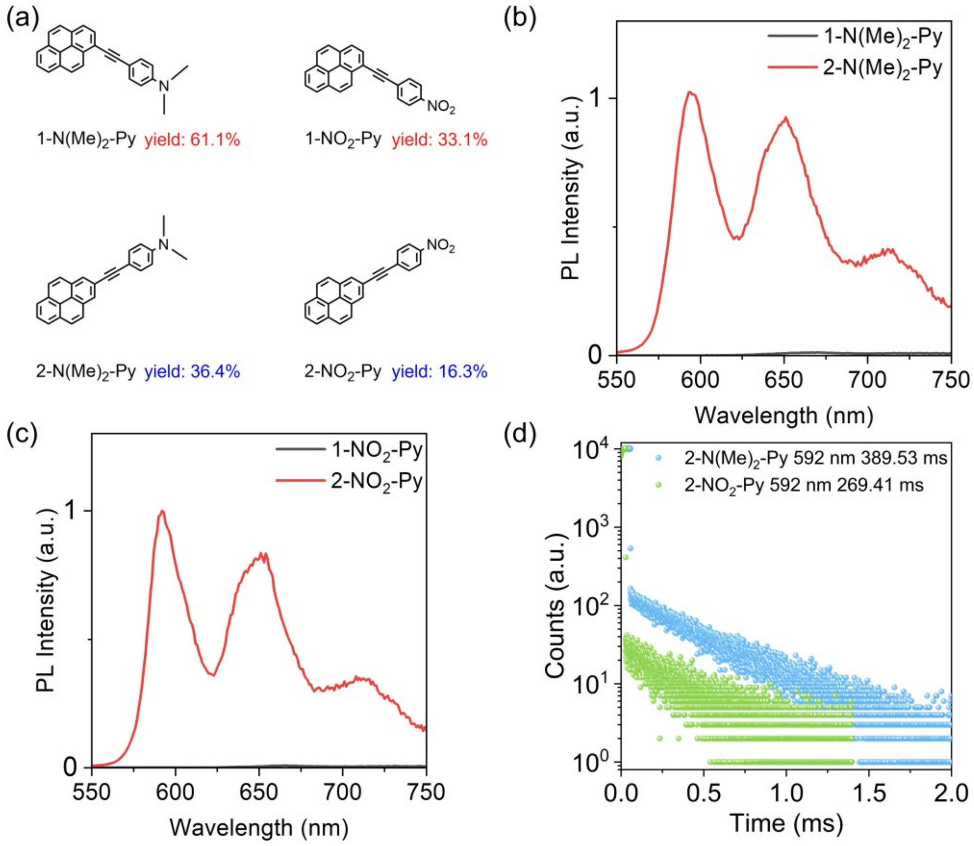

Figure S62. (a) Chemical structures of **1-N(Me)2-Py**, **2-N(Me)2-Py**, **1-NO2-Py**, and **2-NO2-Py**, with coupling yields shown below. (b) Delayed PL spectra of **1-N(Me)2-Py** and **2-N(Me)2-Py** in PMMA recorded at 298 K (doping ratio: 0.5 wt%; gated time: 5–100 ms; λ_{exc} = 298 nm). (c) Delayed PL spectra of **1-NO2-Py**, and **2-NO2-Py** in PMMA recorded at 298 K (doping ratio: 0.5 wt%; gated time: 5–100 ms; λ_{exc} = 298 nm). (d) Time-resolved phosphorescence decay profiles of **2-N(Me)2-Py** and **2-NO2-Py** in PMMA at 298 K (doping ratio: 0.5 wt%; λ_{exc} = 285 nm).

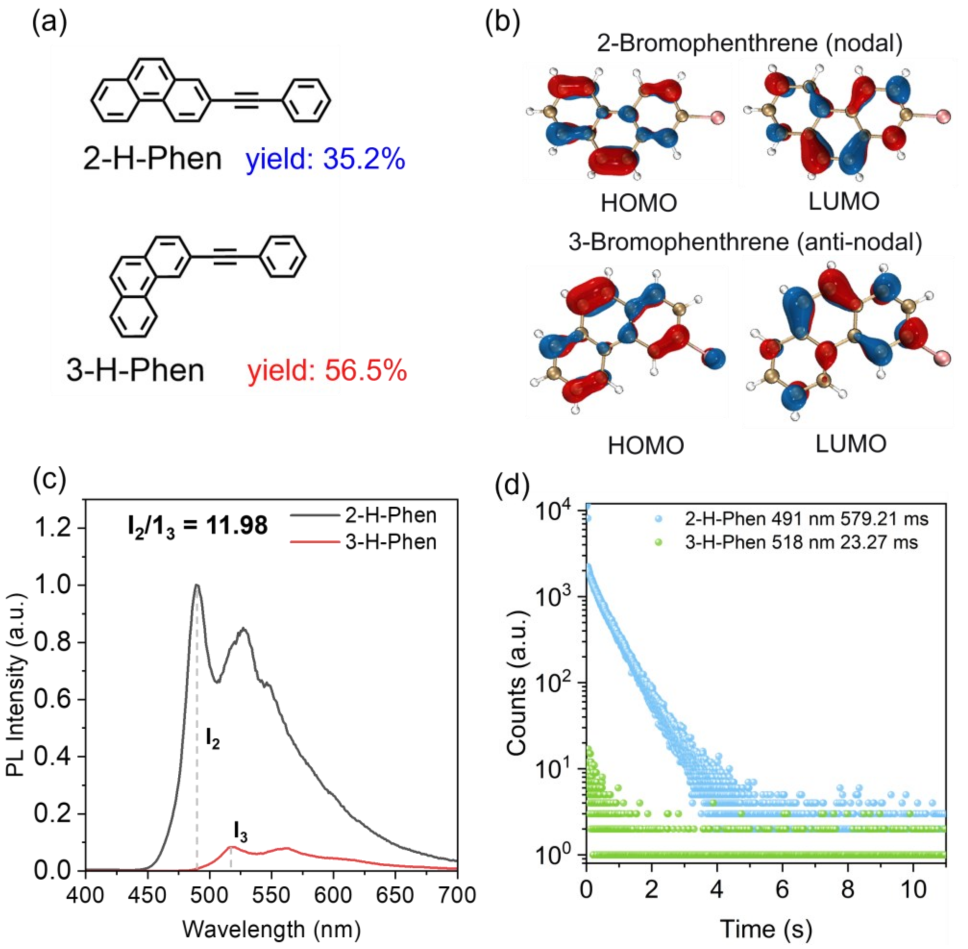

Figure S63. (a) Chemical structures of **2-H-Phen** and **3-H-Phen** with coupling yields shown below. (b) Calculated FMO distributions of 2-Bromophenthrene and 3-Bromophenthrene at optimized S_0 geometries. (c) Delayed PL spectra of **2-H-Phen** and **3-H-Phen** in PMMA recorded at 298 K (doping ratio: 0.5 wt%; λ_{exc} = 298 nm; I_2: phosphorescence intensity of **2-H-Phen**; I_3: phosphorescence intensity of **3-H-Phen**). (d) Time-resolved phosphorescence decay profiles of **2-H-Phen** and **3-H-Phen** in PMMA at 298 K (doping ratio: 0.5 wt%; λ_{exc} = 285 nm).

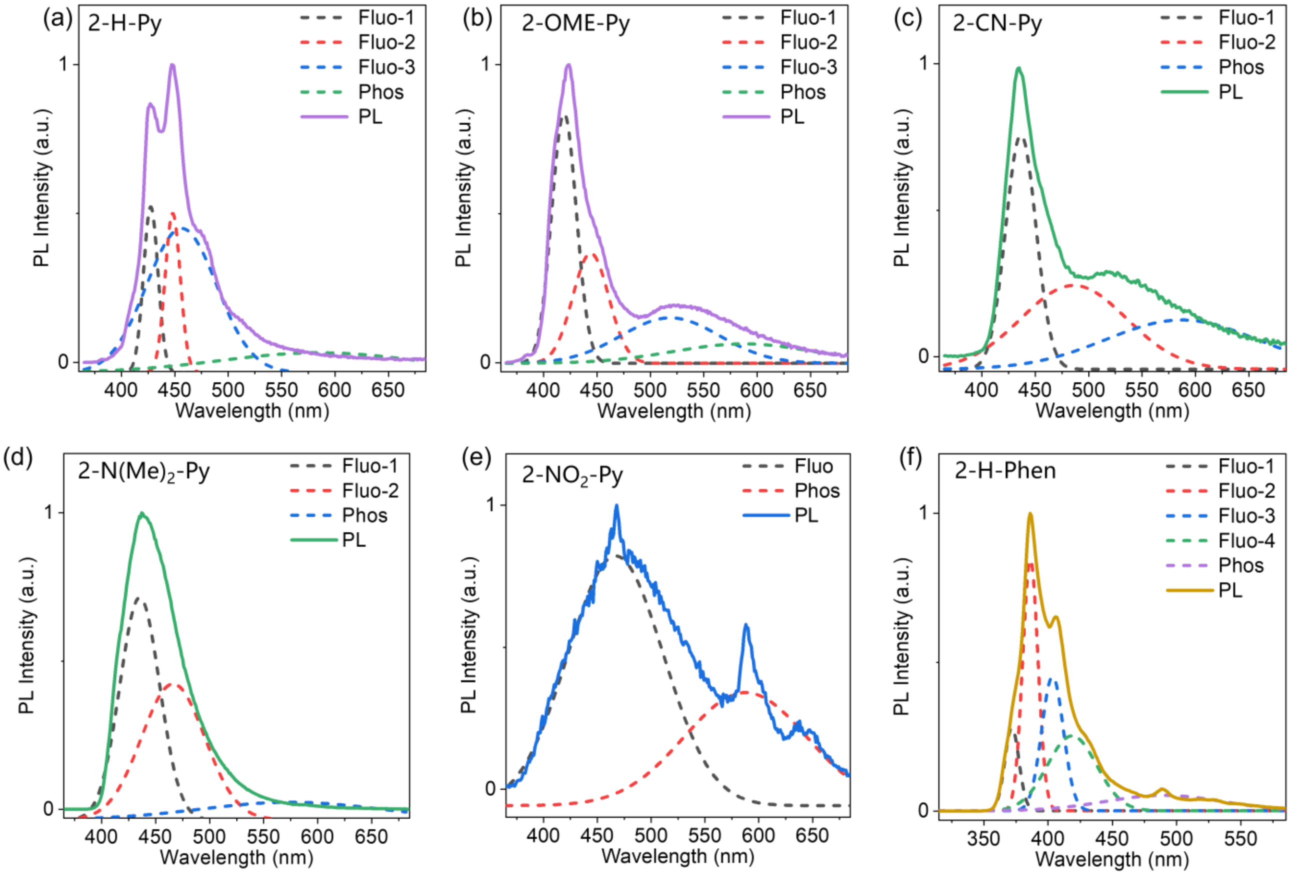

Figure S64. Gaussian deconvolution results of the steady-state PL spectra of (a) **2-H-Py**, (b) **2-OME-Py**, (c) **2-CN-Py**, (d) **2-N(ME)$_2$-Py**, (e) **2-NO$_2$-Py**, and (f) **2-H-Phen** in PMMA at 298 K (doping ratio: 0.5 wt%). Fluo and Phos represent the fluorescence and phosphorescence emissions, respectively.

Ultrafast spectroscopy

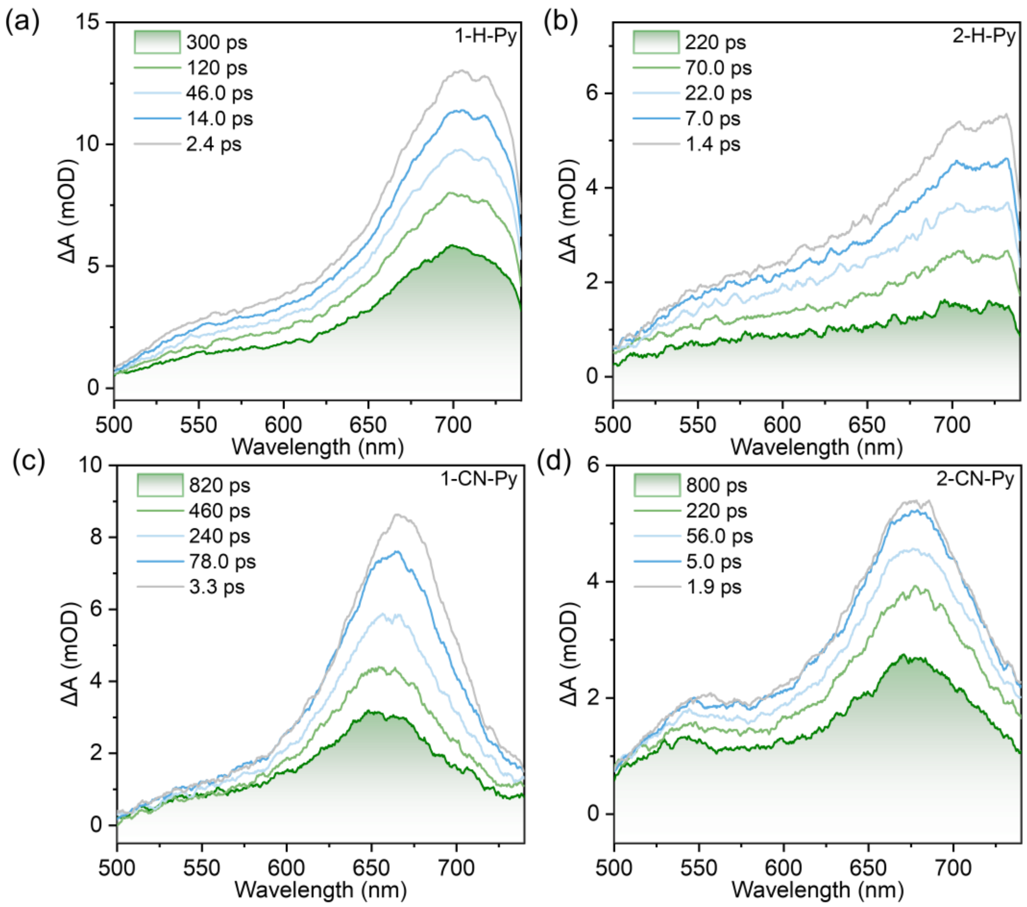

Figure S65. fs-TA spectra of **1-H-Py**, **2-H-Py**, **1-CN-Py**, and **2-CN-Py** in PMMA; doping concentration: 0.5 wt%. (a) **1-H-Py**; (b) **2-H-Py**; (c) **1-CN-Py**; (d) **2-CN-Py**. λ_{exc} = 400 nm.

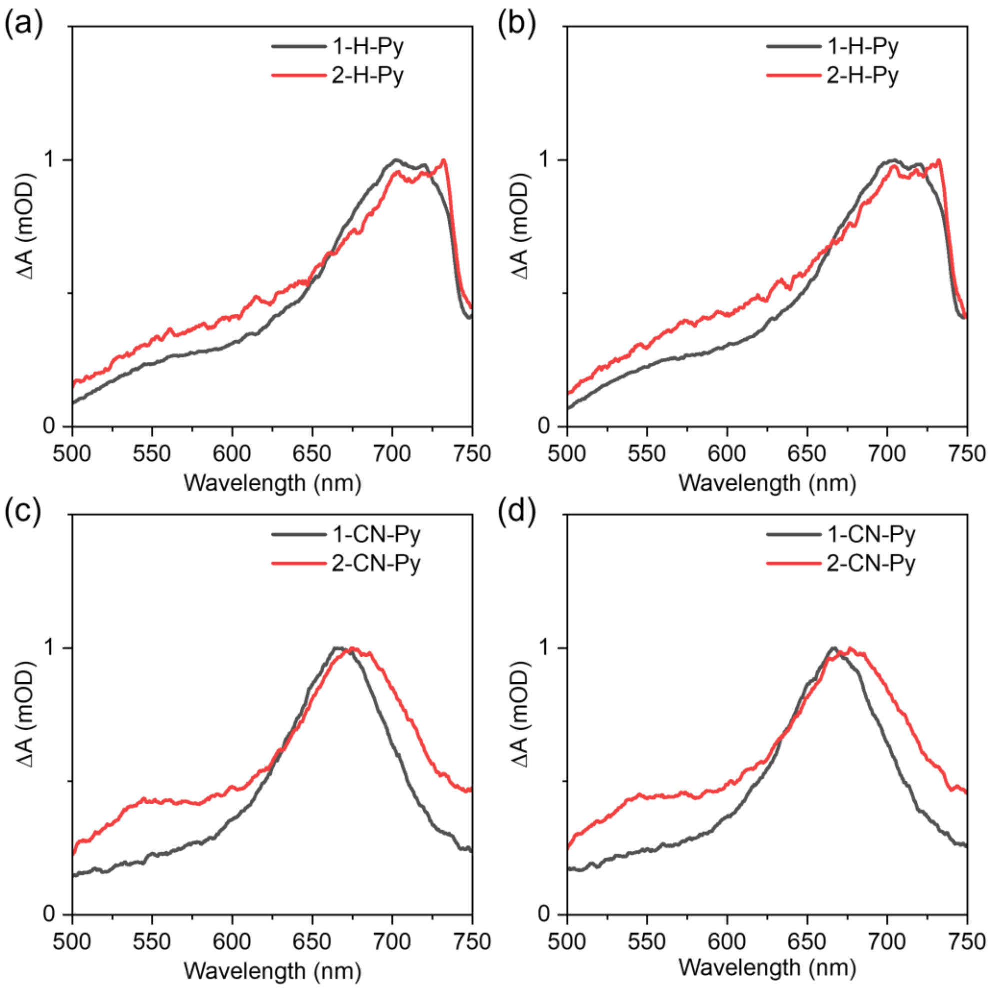

Figure S66. (a) fs-TA intensity maps of **1-H-Py** and **2-H-Py** recorded at 0.5 ns (FWHM: 96 nm for **1-H-Py**, 112 nm for **2-H-Py**). (b) fs-TA intensity maps of **1-H-Py** and **2-H-Py** recorded at 1 ns (FWHM: 95 nm for **1-H-Py**, 116 nm for **2-H-Py**). (c) fs-TA intensity maps of **1-CN-Py** and **2-CN-Py** at 0.5 ns (FWHM: 90 nm for **1-CN-Py**, 124 nm for **2-CN-Py**). (d) fs-TA intensity maps of **1-CN-Py** and **2-CN-Py** at 1 ns (FWHM: 91 nm for **1-CN-Py**, 135 nm for **2-CN-Py**).

Part IV. Other Supplementary Tables

Table S1. Summary of steady-state PL maxima (λ_{PL}) of pyrene derivatives in different solvents recorded at 298 K.

Pyrene derivatives	λ_{PL} (nm)
**1-H-Py** in 2-MeTHF	390
**1-H-Py** in DMF	393
**1-H-Py** in DMSO	395
**2-H-Py** in 2-MeTHF	390
**2-H-Py** in DMF	394
**2-H-Py** in DMSO	401

Concentration: $c = 1 \times 10^{-5}$ mol/L.

Table S2. Summary of steady-state PL maxima (λ_{PL}) and associated lifetimes (τ_{PL}) of pyrene derivatives in 2-MeTHF recorded at 298 and 77 K.

**Pyrene derivatives at 298 K**	**λ_F (nm)**	**τ_{PL} (ns)**
**1-H-Py**	390	2.70
**1-OME-Py**	401	0.55
**1-CN-Py**	420	0.73
**2-H-Py**	390	3.99
**2-OME-Py**	400	2.66
**2-CN-Py**	416	2.00
**Pyrene derivatives at 77 K**	**λ_F (nm)**	**τ_{PL} (ns)**
**1-H-Py**	393	0.14
**1-OME-Py**	401	1.52
**1-CN-Py**	414	1.38
**2-H-Py**	392	2.31
**2-OME-Py**	404	1.71
**2-CN-Py**	408	1.73

Concentration: $c = 1 \times 10^{-5}$ mol/L.

Table S3. The overall PLQYs of the compounds included in this study in 2-MeTHF at 77 K ($c = 1 \times 10^{-5}$ M).

Compound	Φ_{PL} (%)
**1-H-Py**	98.9
**1-OME-Py**	95.5
**1-CN-Py**	93.1
**1-N(Me)$_2$-Py**	86.3
**1-NO$_2$-Py**	49.7
**2-H-Py**	85.5
**2-OME-Py**	33.3
**2-CN-Py**	32.4
**2-N(Me)$_2$-Py**	63.4
**2-NO$_2$-Py**	7.2
**4-H-Py**	92.3
**2-H-Phen**	7.7
**3-H-Phen**	18.9

Table S4. Summary of phosphorescence emission maxima (λ_{Ph}) and associated lifetimes (τ_{Ph}) of pyrene derivatives in 2-MeTHF recorded at 77 K.

Pyrene derivatives	λ_{Ph} (nm)	τ_{Ph} (s) at 587 nm	τ_{Ph} (s) at 649 nm
**2-H-Py**	587	0.54 s	0.48 s
**2-OME-Py**	587	0.69 s	0.63 s
**2-CN-Py**	587	0.74 s	0.68 s

Concentration: c = 1 × 10^{-5} mol/L.

Table S5. Summary of steady-state PL maxima (λ_{PL}) and associated lifetimes (τ_{PL}) of pyrene derivatives doped in PMMA (doping ratio: 0.5 wt%) recorded at 298 K.

Pyrene derivatives	λ_{PL} (nm)	τ_{PL} (ns)
**1-H-Py**	418	2.98
**1-OME-Py**	430	0.59
**1-CN-Py**	457	1.19
**2-H-Py**	448	3.14
**2-OME-Py**	424	4.92
**2-CN-Py**	434	3.02

Table S6. Summary of steady-state PL maxima (λ_{PL}) and associated lifetimes (τ_{PL}) of pyrene derivatives doped in PMMA (doping ratio: 0.5 wt%) recorded at 77 K.

Pyrene derivatives	λ_{PL} (nm)	τ_{PL} (ns)
**1-H-Py**	417	2.63
**1-OME-Py**	427	0.98
**1-CN-Py**	454	2.55
**2-H-Py**	446	7.17
**2-OME-Py**	423	3.69
**2-CN-Py**	433	5.29

Table S7. Summary of phosphorescence emission maxima (λ_{Ph}) and associated lifetimes (τ_{Ph}) of pyrene derivatives doped in PMMA (doping ratio: 0.5 wt%) recorded at 298 K.

Pyrene derivatives	λ_{Ph} (nm)	τ_{Ph} (ms)
**2-H-Py**	588	82.45
**2-OME-Py**	590	74.70
**2-CN-Py**	591	99.98

Table S8. Summary of phosphorescence emission maxima (λ_{Ph}) and associated lifetimes (τ_{Ph}) of pyrene derivatives doped in PMMA (doping ratio: 0.5 wt%) recorded at 77 K.

Pyrene derivatives	λ_{Ph} (nm)	τ_{Ph} (s)
**2-H-Py**	590	0.44
**2-OME-Py**	587	0.29
**2-CN-Py**	589	0.56

Table S9. The estimated rate constants and PLQYs of RTP compounds in PMMA recorded at 298 K (doping ratio: 0.5 wt%).

Compound	Φ_{PL} (%)	Φ_f (%)	Φ_{ph} (%)	τ_f (ns)	k_r^S (10^7 s^{-1})	k_{isc} (10^7 s^{-1})	τ_{ph} (ms)	k_r^T (s^{-1})	k_{nr}^T (s^{-1})
**2-H-Py**	12.3	10.1	2.2	3.14	3.22	28.63	82.45	0.30	11.83
**2-OME-Py**	21.9	18.6	3.3	4.92	3.78	16.54	74.7	0.54	12.84
**2-CN-Py**	39.1	27.3	11.8	3.02	9.04	24.07	99.98	1.62	8.38
**2-N(Me)$_2$-Py**	21.5	18.5	3	49.63	0.37	1.64	389.53	0.09	2.47
**2-NO$_2$-Py**	1.9	1.2	0.7	6.21	0.19	15.91	269.41	0.03	3.69
**2-H-Phen**	23.3	19.2	4.1	50.99	0.38	1.58	579.21	0.09	1.64

The Φ_f and Φ_{ph} values were estimated by applying Gaussian spectral deconvolution to the total emission spectra, using the relative integrated areas of the separated peaks (**Figure S64**). Given the large S_1–S_0 energy gap (blue fluorescence) and the fast singlet radiative decay, we assume the singlet nonradiative decay is negligible ($k_{nr}^S \approx 0$) in accordance with the energy gap law. This allows the estimation of the remaining rate constants using the following equations.

$\Phi_f = k_r^S/(k_r^S + k_{ISC})$ (1)

$\tau_f = 1/(k_r^S + k_{ISC})$ (2)

$\Phi_{ISC} = k_{ISC}/(k_r^S + k_{ISC}) = k_{ISC} \times \tau_f$ (3)

$\Phi_{ph} = \Phi_{ISC} \times k_r^T/(k_r^T + k_{nr}^T) = \Phi_{ISC} \times k_r^T \times \tau_{ph}$ (4)

From these, the specific rate constants are derived as:

$k_r^S = \Phi_f/\tau_f$ (5)

$k_{ISC} = (1/\tau_f) - k_r^S$ (6)

$k_r^T = \Phi_{ph}/(k_{ISC} \times \tau_f \times \tau_{ph})$ (7)

$k_{nr}^T = (1/\tau_{ph}) - k_r^T$ (8)

Here, Φ_f, Φ_{ph} and Φ_{ISC} represent fluorescence, phosphorescence, and ISC QYs, respectively; τ_f and τ_{ph} are the fluorescence and phosphorescence lifetimes; k_r^S and k_r^T respresent singlet and triplet radiative decay rates, respectively; k_{nr}^S and k_{nr}^T represent singlet and triplet nonradiative decay rates, respectively.
